# Abatacept versus hydroxychloroquine for prevention of rheumatoid arthritis in individuals with palindromic rheumatism: a randomized open-label trial

**DOI:** 10.1038/s41591-026-04395-6

**Published:** 2026-05-14

**Authors:** Raimon Sanmarti, Carolina Pérez-García, Francisco J. de-Toro, Georgina Salvador, Alejandro Escudero-Contreras, Andrea Cuervo, Eduard Graell, Delia Reina, Eduardo Kanterewicz, Hèctor Corominas, Irati Urionaguena-Onaindia, Maria López-Lasanta, Alejandro Olivé, Miquel Sala-Gómez, Beatriz Frade-Sosa, Rosa M. Morlà-Novell, Luciano Polino, Juan Antonio Meraz-Ostiz, Natividad Oreiro, Francisco J. Blanco, Inma Pérez-Nadales, Rafaela Ortega-Castro, Noemi Busquets-Pérez, Antonio Domingo Gómez-Centeno, Oscar Camacho, José R. Rodríguez-Cros, Ana Milena Millan-Arciniegas, José Francisco García-Llorente, Helena Borrell, Águeda Prior-Español, Sonia Castell-Quiñones, Ana Cruceta, Eva Bonfill, Gemma Domenech-Gómez, Andreu Roca-Fàbregas, José Ríos, Lola Tobalina-Maestre, María José Gómara, Isabel Haro

**Affiliations:** 1 Rheumatology, Hospital Clinic de Barcelona, Barcelona, Spain; Institut d’Investigacions Biomèdiques August Pi i Sunyer (IDIBAPS), Barcelona, Spain; 2 Rheumatology, Hospital del Mar, Barcelona, Spain; 3 Rheumatology, Complexo Hospitalario Universitario de A Coruña, Instituto de Investigación Biomédica de A Coruña (INIBIC), Universidad de A Coruña (UDC), A Coruña, Spain; 4 Rheumatology, Hospital Universitari Mútua Terrassa, Terrassa, Spain; 5 Rheumatology, Reina Sofía University Hospital, IMIBIC, UCO, Córdoba, Spain; 6 Rheumatology, Hospital General de Granollers, Granollers, Spain; 7 Rheumatology, Parc Taulí Hospital Universitari, I3PT, Universitat Autonoma de Barcelona, Sabadell, Spain; 8 Rheumatology, Complex Hospitalari Universitari Moises Broggi, Sant Joan Despí, Spain; 9 Rheumatology, Hospital Universitari de Vic, Vic, Spain; 10 Rheumatology, Hospital de la Santa Creu i Sant Pau, Barcelona, Spain; 11 Rheumatology, Hospital Universitario Galdakao-Usansolo, Bizkaia, Spain; 12 Rheumatology, Hospital Vall d´Hebron, Barcelona, Spain; 13 Rheumatology, Hospital Universitari Germans Trias i Pujol, Badalona, Spain; 14 Rheumatology, Hospital de Figueres, Figueres, Spain; 15 Clinical Research Support Unit (CTU), Core facility. HCB-IDIBAPS. Hospital Clínic, Barcelona, Spain; 16 Medical Statistics Core Facility, Fundació de Recerca Clínic Barcelona (FRCB)- Institut d’investigacions Biomèdiques August Pi i Sunyer (IDIBAPS), Barcelona, Spain; 17 Department of Clinical Pharmacology, Hospital Clinic and Medical Statistics Core Facility, Institut d’Investigacions Biomèdiques August Pi i Sunyer (FCRB-IDIBAPS), Barcelona, Spain. Biostatistics Unit, School of Medicine, Universitat Autònoma de Barcelona, Barcelona, Spain; 18 Biological Chemistry, Unit of Synthesis and Biomedical Applications of Peptides, Institute of Advanced Chemistry of Catalonia- Consejo Superior de Investigaciones Científicas (IQAC-CSIC), Barcelona, Spain

**Keywords:** Translational research, Rheumatic diseases

## Abstract

A substantial proportion of individuals with palindromic rheumatism develop rheumatoid arthritis (RA). This randomized, open-label, multicenter trial aimed to assess whether 2 years of treatment with abatacept (*n* = 34; 125 mg subcutaneous injections weekly during the first year and every 2 weeks during the second year) compared with oral hydroxychloroquine (*n* = 36; 5 mg kg^−^^1^ per day) could reduce the frequency of RA development in individuals with palindromic rheumatism positive for rheumatoid factor and/or anticitrullinated protein antibody. The primary outcome was the development of persistent arthritis that fulfilled the 2010 RA classification criteria of the American College of Rheumatology and the European Alliance of Associations for Rheumatology, as evaluated by the participant clinicians during the 24 months of follow-up. Secondary outcomes included the frequency, intensity and duration of joint attacks, the proportion of patients in remission and the frequency of adverse events. In the primary analysis, in the modified full analysis set with failure imputation, 7 (20.6%) of the 34 participants treated with abatacept and 18 (50.0%) of the 36 participants treated with hydroxychloroquine developed RA during the 24 months of follow-up (*P* = 0.010; risk difference 29.4%, 95% confidence interval 8.2 to 50.7), meeting the primary endpoint. Using the available-data-only approach, the corresponding figures were 3 (10.0%) of 30 individuals and 10 (35.7%) of 28 individuals, respectively (*P* = 0.019). Compared with participants treated with hydroxychloroquine, participants treated with abatacept had a significantly longer time to progression to RA (hazard ratio 0.27, 95% confidence interval 0.07 to 0.96; log-rank test *P* = 0.0299). Abatacept was also associated with a reduced intensity of joint attacks and a higher frequency of symptom remission; however, there were no differences in the frequency of attacks between the two study drugs. No relevant differences in the evolution of antimodified peptide and/or protein antibody titers were observed between the two treatment arms. Both drugs were well tolerated. In patients with seropositive palindromic rheumatism, compared with hydroxychloroquine, abatacept given for 2 years reduced the risk of progression to RA and improved symptoms. ClinicalTrials.gov identifier NCT03669367 and EudraCT no. 2017-004543-20.

## Abstract (web summary)

Subcutaneous injections of abatacept were superior to oral hydroxychloroquine in preventing progression to persistent arthritis in individuals with palindromic rheumatism.

## Main

Palindromic rheumatism (PR) is a clinical syndrome characterized by sudden, recurrent and usually short-lasting attacks of pain and swelling around the joints without residual joint damage^1,2^. A high proportion of individuals with PR develop RA (30–60% in most series)^1^, and a variable but high proportion (39–68%) present an autoantibody profile similar to that observed in individuals with RA, with increased levels of rheumatoid factor (RF) and/or anticitrullinated protein antibody (ACPA)^2^. This autoantibody profile has also been observed in the preclinical phase of RA and in individuals at risk of developing RA, and it is considered the strongest predictor for developing RA in individuals at risk^3^, including those with PR^4^. Consistent with these findings, it has been suggested that PR could be a form of preclinical RA^1^. If we are able to identify individuals at higher risk of developing RA, a window of opportunity is opened to intervene by addressing modifiable risk factors or initiating pharmacological treatment that could delay or prevent the occurrence of the disease^5^.

Three placebo-controlled trials have shown the efficacy of biologic disease-modifying anti-rheumatic drugs (DMARDs) in delaying the onset of RA with the anti-B cell rituximab^6^ or preventing its onset with the T cell down-modulator abatacept^7,8^ in individuals with arthralgia but without arthritis and at a high risk of the disease defined by the presence of some risk factors, such as the presence of autoantibodies and/or subclinical synovitis detected by imaging techniques.

In individuals with PR, antimalarials, such as hydroxychloroquine and chloroquine, are considered the best-studied therapeutic option, although there are no randomized clinical trials supporting their efficacy and safety^9^. In addition to their beneficial clinical effects in controlling palindromic symptoms, an observational study conducted in individuals with PR showed that those treated with antimalarials demonstrated a significantly longer time to develop RA than those who were not treated with antimalarials^10^. The key role of T cell activation in the preclinical and early phases of RA and the efficacy and safety of its inhibition with abatacept in both early and established RA, with an effect more marked in ACPA-positive patients, together with its effect in preventing the development of RA in individuals at risk of the disease^7,8,11,12^, position abatacept as a suitable alternative for a prevention trial in individuals with PR at risk of RA.

This randomized, open-label, multicenter trial aimed to assess whether 2 years of treatment with abatacept compared to hydroxychloroquine could reduce the frequency of RA development in patients with PR positive for RF and/or ACPA.

## Results

**Fig. 1:**
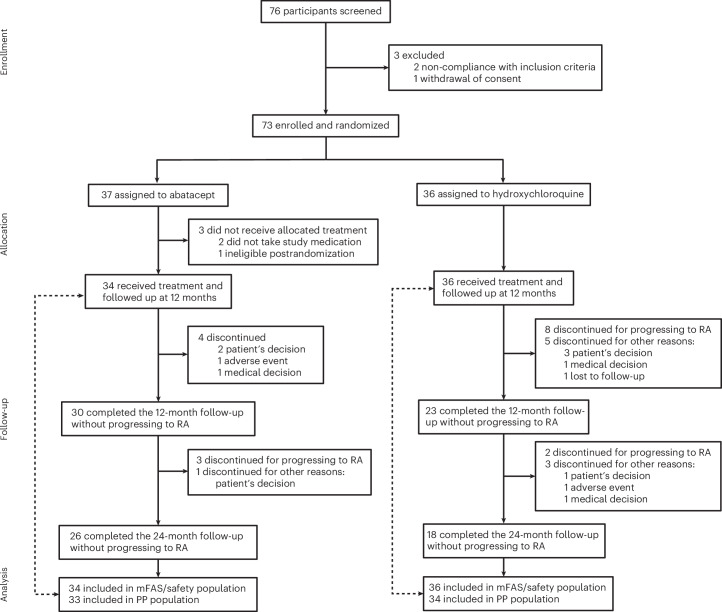
Participant disposition. Number of screened and randomized individuals, number of discontinuations, and number of individuals included in each population for analyses.

Between 3 June 2019 and 12 July 2022, 76 individuals were screened for participation and 73 were randomly assigned to either abatacept (*n* = 37) or hydroxychloroquine (*n* = 36) (Fig. 1). The three screening failures consisted of one individual with radiographic damage (joint erosion), one individual who met the criteria for other rheumatic diseases and one individual who withdrew consent before randomization. Three participants from the abatacept group did not receive the study medication, leading to 70 individuals being included in the modified full analysis set (mFAS) and safety population. Thirty-three individuals in the abatacept group and 34 in the hydroxychloroquine group showed adherence greater than 75%, leading to the inclusion of 67 individuals in the per-protocol (PP) population. Twenty-six (76.5%) of the 34 individuals assigned to abatacept and 18 (50%) of the 36 individuals assigned to hydroxychloroquine included in the mFAS completed the study.

The participants were middle-aged, predominantly female and white European (Table 1). A greater percentage of participants in the hydroxychloroquine group had a duration of attacks ≥72 h within 6 months prior to inclusion (30.8% versus 11.5%) and a higher frequency of ACPA positivity (97.2% versus 82.4%). The remaining characteristics were well balanced between the study groups (Table 1).

**Table 1:** Baseline characteristics of the participants in the mFAS

	Hydroxychloroquine (*n* = 36)	Abatacept (*n* = 34)
Sex		
Male	11 (30.6%)	9 (26.5%)
Female	25 (69.5%)	25 (73.5%)
Age, years; median (P25, P75)	50.5 (41.5, 57)	53.5 (45, 61)
Ethnicity		
White	32 (88.9%)	27 (79.4%)
Hispanic	3 (8.3%)	7 (20.6%)
Other	1 (2.8%)	0 (0%)
Smoking status		
Never	13 (36.1%)	15 (44.1%)
Previous	10 (27.8%)	10 (29.4%)
Current	13 (36.1%)	9 (26.5%)
Body mass index, kg m^−^^2^; median (P25, P75)	25.8 (23.4, 31.3)	25.2 (23.9, 27.2)
Symptom duration, months; median (P25, P75)	8 (6, 11.5)	8 (5, 12)
Number of attacks per month^a^; median (P25, P75)	0.67 (0.33, 1.25)	0.67 (0.33, 0.83)
Duration of attacks^a,b^		
<72 h	137/198 (69.2%)	115/130 (88.5%)
≥72 h	61/198 (30.8%)	15/130 (11.5%)
Intensity of attacks^a,b^, visual analog scale 0–10; median (P25, P75)	*n* = 146; 7 (5, 8)	*n* = 118; 7 (5, 8)
Anti-inflammatory treatment^a^		
Nonsteroidal anti-inflammatory drugs	12 (33.3%)	11 (32,4%)
Oral glucocorticoids	7 (19.4%)	3 (8.8%)
Number of joint involved^a^		
Monoarticular	27 (75.0%)	27 (79.4%)
Oligo-/polyarticular	9 (25.0%)	7 (20.6%)
Joints involved in the attacks^a^		
Wrist	23 (63.9%)	23 (67.6%)
Metacarpophalangeal	25 (69.4%)	17 (50%)
Proximal interphalangeal	9 (25.0%)	10 (29.4%)
Shoulder	14 (38.9%)	14 (41.2%)
Knee	11 (30.6%)	7 (20.6%)
Elbow	5 (13.9%)	4 (11.8%)
Hip	2 (5.6%)	5 (14.7%)
Foot (any location)	13 (36.1%)	7 (20.6%)
Other	2 (5.6%)	3 (8.8%)
Serology		
ACPA positive	35 (97.2%)	28 (82.4%)
RF positive	28 (77.8%)	29 (85.3%)
ACPA and RF positive	27 (75.0%)	23 (67.6%)
C-reactive protein, mg dl^−1^; mean (s.d.)	0.61 (0.55)	0.53 (0.54)
Erythrocyte sedimentation rate, mm h^−^^1^; mean (s.d.)	19.8 (14.4)	23 (18.3)

^a^Within 6 months prior to inclusion.

^b^Calculated over the total number of attacks with information.

The use of allowed anti-inflammatory therapy (that is, colchicine, oral glucocorticoids and nonsteroidal anti-inflammatory drugs) throughout the study period was less frequent among individuals treated with abatacept than among those treated with hydroxychloroquine, except for colchicine, with one patient treated in the abatacept group compared to none in the hydroxychloroquine group (Supplementary Table 1). Regardless of the indication, less participants received oral glucocorticoids among those treated with abatacept (*n* = 7, 20.6%) than among those treated with hydroxychloroquine (*n* = 19, 52.8%) (Supplementary Table 1).

### Occurrence of rheumatoid arthritis

**Fig. 2:**
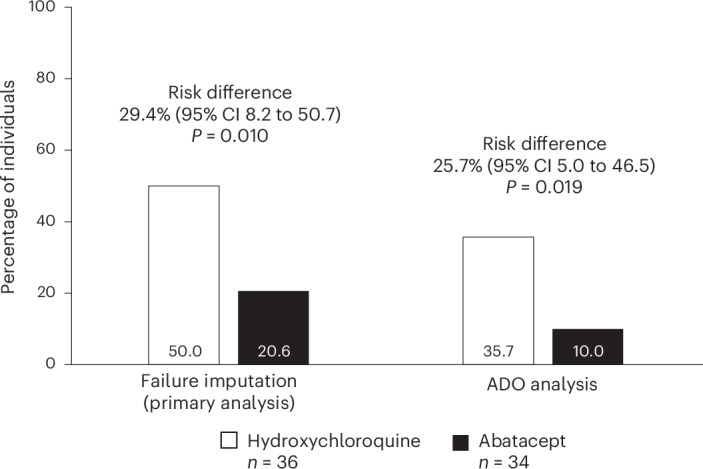
Development of RA in the mFAS population. The number of individuals in the primary analysis (that is, failure imputation) was 36 and 34 in the hydroxychloroquine and abatacept groups, respectively. The number of individuals in the ADO analysis was 28 and 30 in the hydroxychloroquine and abatacept groups, respectively. *P* values for comparisons of abatacept with hydroxychloroquine were calculated using two-sided chi-square test without multiplicity adjustment.

In the primary analysis with failure imputation, 7 (20.6%) of the 34 participants treated with abatacept and 18 (50.0%) of the 36 participants treated with hydroxychloroquine developed RA during the 24 months of follow-up (*P* = 0.010); the risk difference was 29.4% (95% confidence interval (CI) 8.2 to 50.7). The results obtained using the available data only (ADO) approach in the mFAS population were consistent with those of the primary analysis (Fig. 2). A post hoc analysis accounting for the imbalance in the two variables mentioned above (that is, duration of attacks ≥72 h within 6 months prior to inclusion and frequency of ACPA positivity) was performed using logistic regression analysis in two ways: adjusting for each single covariate and adjusting for the two covariates. The results of the sensitivity analysis were consistent with those of the primary analysis (Supplementary Tables 2–5).

During the first 12 months, using failure imputation in the mFAS population, 3 (8.8%) of the 34 participants treated with abatacept and 13 (36.1%) of the 36 participants treated with hydroxychloroquine developed RA (*P* = 0.007), with consistent results when the ADO approach were used (Supplementary Table 6). Abatacept was also significantly superior to hydroxychloroquine in reducing the risk of developing RA during the first 12 months in the PP population (Supplementary Table 6).

**Fig. 3:**
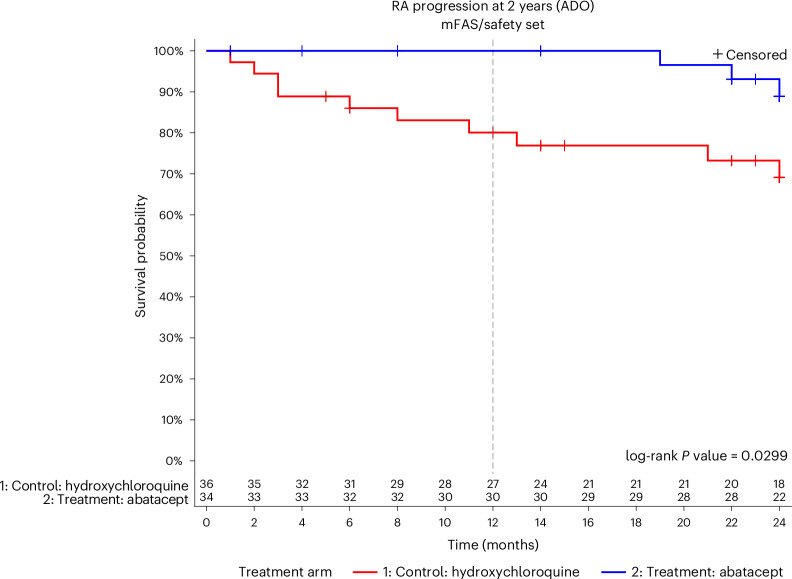
RA-free survival in the mFAS population. *P* values for comparisons of abatacept with hydroxychloroquine were calculated using two-sided log-rank test without multiplicity adjustment. The HR for the comparison derived from a Cox model was 0.27 (95% CI 0.07 to 0.96).

In the time-to-event analysis, abatacept-treated participants showed a significantly longer time to progression to RA than those treated with hydroxychloroquine (hazard ratio (HR) 0.27, 95% CI 0.07 to 0.96; log-rank test *P* = 0.0299) (Fig. 3).

### Effects on symptoms

**Fig. 4:**
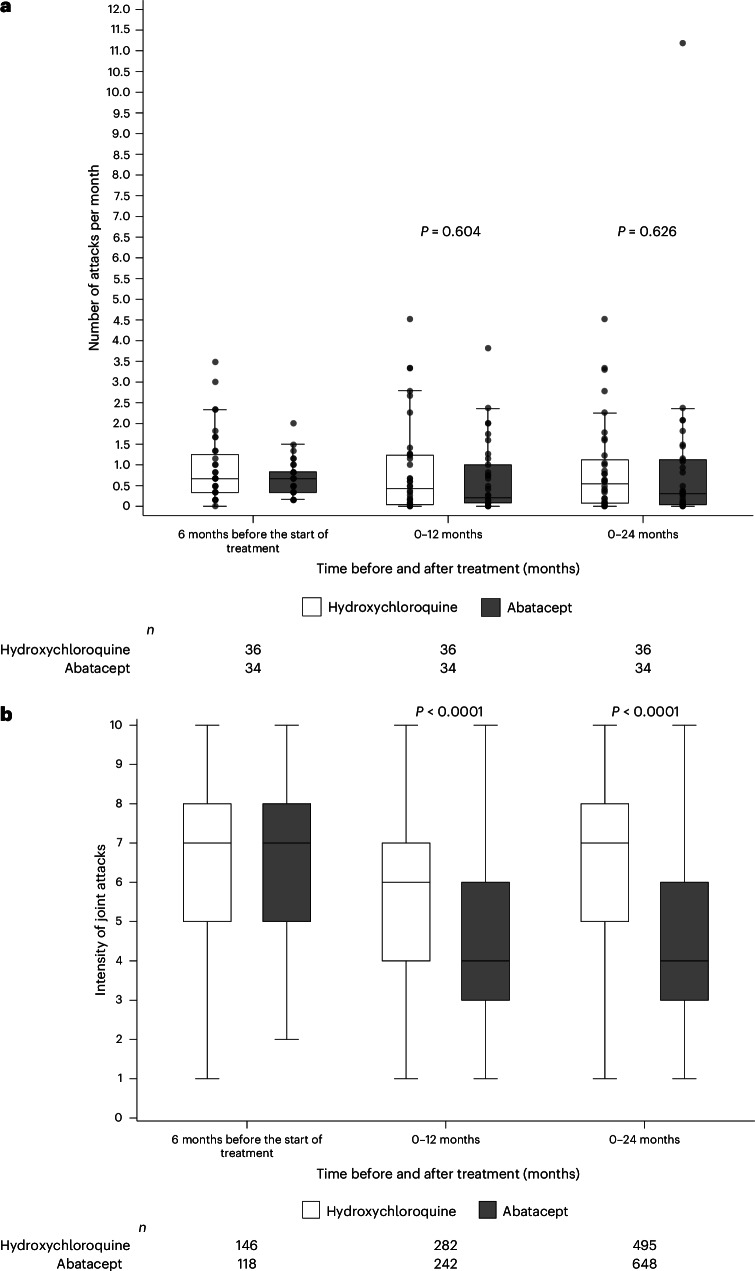
Course of attacks in individuals with PR. **a**, Course of frequency of attack per month in the mFAS population. Box plots with the median (P25, P75), minimum and maximum. There were 36 and 34 individuals in the hydroxychloroquine and abatacept groups, respectively. *P* values for comparisons of abatacept with hydroxychloroquine were calculated using two-sided Mann‒Whitney *U*-test without multiplicity adjustment. **b**, Course of intensity of attacks in the mFAS population. Box plots with the median (P2, P75), minimum and maximum. There were 706 and 793 attacks in the hydroxychloroquine and abatacept groups, respectively. *P* values for comparisons of abatacept with hydroxychloroquine were calculated using two-sided Mann‒Whitney *U*-test without multiplicity adjustment.

The median number of attacks per patient from 6 months prior to randomization was reduced in both study groups, but the differences in the median number of attacks between the abatacept and hydroxychloroquine groups were not statistically significant either during the 24-month study period or during the first 12 months (Fig. 4a). However, during the 24-month period, the proportion of patients in remission in the mFAS population was 19 (55.9%) of the 34 participants treated with abatacept, and 8 (22.9%) of the 36 participants treated with hydroxychloroquine (*P* = 0.007) (Supplementary Table 7).

The median (25th percentile (P25), 75th percentile (P75)) intensity of the attacks was reduced from 7.0 (5.0, 8.0) in the 6 months prior to randomization in both study groups to 4.0 (3.0, 6.0) during the 24-month period in the abatacept group, whereas it remained unchanged in the hydroxychloroquine group (7.0 (5.0, 8.0)). The differences in the intensity of attacks during the 24-month period between the two study groups were statistically significant (Fig. 4b). The duration of attacks was reduced in both study groups throughout the study; the proportion of attacks with a duration of <24 h in the abatacept group was greater than that in the hydroxychloroquine group during the 24-month period (31.2% versus 16.6%) (Supplementary Table 8).

### Effect on autoantibodies

With respect to IgG autoantibodies, there were no significant differences between the two study groups, except for chimeric fibrin/filaggrin homocitrullinated peptide-IgG at month 24, which showed a lower geometric mean (GM) of Δ optical density values in participants treated with abatacept than in those treated with hydroxychloroquine (GM ratio 0.18, 95% CI 0.05 to 0.59; *P* = 0.0058) (Supplementary Table 9).

There were significant differences between the two study groups in the CEP 1-IgA at month 3, which showed a lower GM of Δ optical density values in participants treated with abatacept compared with those treated with hydroxychloroquine (GM ratio 0.72, 95% CI 0.51 to 1.00; *P* = 0.0471), and for the chimeric fibrin/filaggrin homocitrullinated peptide-IgA at 24 months, which showed a lower GM of Δ optical density values in participants treated with abatacept compared with those treated with hydroxychloroquine (GM ratio 0.28, 95% CI 0.08 to 0.93; *P* = 0.0374) (Supplementary Table 10).

Regarding IgM autoantibodies, there were no significant differences between the two study groups in the Δ optical density values at any timepoint throughout the study (Supplementary Table 11).

### Safety evaluations

Overall, 25 (73.5%) of the 34 participants treated with abatacept and 23 (63.9%) of the 36 participants treated with hydroxychloroquine experienced at least one adverse event (Table 2). One participant treated with abatacept experienced a serious adverse event, which consisted of melena requiring hospitalization and was not considered related to the study drug. Two participants treated with hydroxychloroquine experienced five serious adverse events (one individual experienced two episodes of diverticulitis, antral gastritis and a sigmoidectomy surgical procedure, and one individual had a nonfatal myocardial infarction). There were no cases of adverse events leading to death, and there was one case of adverse events leading to discontinuation among abatacept-treated participants consisted of a case of nonserious toxicoderma. The most frequent adverse events reported with either drug are presented in Table 2.

**Table 2:** Adverse events

Variable	Hydroxychloroquine (*n* = 36) occurrences/participants (%)	Abatacept (*n* = 34) occurrences/participants (%)	*P* value
Any adverse events	89/23 (63.9%)	75/25 (73.5%)	0.446
Any severe adverse event	1/1 (2.8%)	1/1 (2.9%)	1.000
Any serious adverse event	5/2 (5.6%)	1/1 (2.9%)	1.000
Any adverse event leading to death	0/0 (0.0%)	0/0 (0.0%)	
Any adverse event leading to discontinuation	0/0 (0.0%)	1/1 (2.9%)	0.486
Most frequent (≥5% in either group) adverse event by System Order Class			
Infections and infestations	14/12 (33.3%)	23/18 (52.9%)	0.147
Musculoskeletal and connective tissue disorders	24/12 (33.3%)	9/5 (14.7%)	0.096
Gastrointestinal disorders	11/7 (19.4%)	4/4 (11.8%)	0.515
Nervous system disorders	11/7 (19.4%)	3/3 (8.8%)	0.308
Respiratory; thoracic and mediastinal disorders	2/2 (5.6%)	7/5 (14.7%)	0.253
General disorders and administration site conditions	4/4 (11.1%)	0/0 (0.0%)	0.115
Injury; poisoning and procedural complications	1/1 (2.8%)	4/4 (11.8%)	0.192
Skin and subcutaneous tissue disorders	6/4 (11.1%)	5/3 (8.8%)	1.000
Immune system disorders	2/1 (2.8%)	3/3 (8.8%)	0.350
Psychiatric disorders	3/3 (8.3%)	1/1 (2.9%)	0.615
Metabolism and nutrition disorders	3/2 (5.6%)	2/2 (5.9%)	1.000
Reproductive system and breast disorders	0/0 (0.0%)	2/2 (5.9%)	0.232
Vascular disorders	0/0 (0.0%)	3/2 (5.9%)	0.232
Cardiac disorders	2/2 (5.6%)	0/0 (0.0%)	0.493
Eye disorders	3/2 (5.6%)	4/4 (1.8%)	0.422

*P* values for comparisons of abatacept with hydroxychloroquine were calculated using two-sided Fisher’s exact test without multiplicity adjustment.

With respect to biochemistry and hematological parameters, the estimated treatment differences for the mean changes from baseline at the end of the study were statistically significant for total cholesterol (*P* = 0.003) and lymphocytes (*P* < 0.001), which increased with abatacept treatment and decreased with hydroxychloroquine (Supplementary Table 12).

## Discussion

The results of this open-label, randomized clinical trial revealed that, compared with hydroxychloroquine, abatacept reduced the risk of developing RA in patients with PR at risk of disease occurrence. This benefit appeared to extend to a greater improvement in PR symptoms among abatacept-treated individuals. Both drugs were well tolerated over a period of 2 years.

As we know of no other randomized clinical trials in patients with PR, we cannot compare our results with those of other trials of individuals with PR. Two retrospective studies have reported the risk of occurrence of RA in individuals with PR treated with antimalarials, mostly chloroquine^10,13^. Youssef et al.^13^, with a mean follow-up of 3.6 years, reported that 12 (23.5%) of 51 individuals treated with antimalarials developed persistent arthritis, and Gonzalez-Lopez et al.^10^ reported that 15 (24.2%) of 62 treated individuals developed RA. Despite the different designs, diagnostic criteria and definitions of the outcomes, and given that we used an enriched population, these figures are similar to our findings with hydroxychloroquine in the ADO population. Compared with no treatment, Gonzalez-Lopez et al.^10^ reported that treatment with antimalarials significantly prolonged the time to the occurrence of RA (HR 0.24, 95% CI 0.09 to 0.61), supporting to some extent the selection of our comparator. Most recently, beneficial effects have been observed with methotrexate in two observational studies (in most patients administered in combination with hydroxychloroquine) in individuals with PR^14,15^. To our knowledge, only one retrospective study has been performed with a biological drug, rituximab, in patients with refractory PR, mostly in combination with DMARDs, with good results in terms of symptom control, and none of the 33 individuals progressed to RA during a mean follow-up of 24 months^16^.

Our results for abatacept in individuals with PR are supported by the results of this biologic DMARD in placebo-controlled randomized trials in other populations of individuals with a prearthritis state at risk of developing RA. The APIPPRA study included individuals with inflammatory joint pain who were positive for ACPA or RF and reported a significantly longer time to the occurrence of RA with abatacept than with placebo for 24 months^8^. Throughout the first 12 months of active treatment, the incidence of RA was 6% with abatacept and 29% with placebo^8^. The ARIAA trial recruited patients with arthralgia, ACPA positivity and signs of subclinical inflammation on magnetic resonance imaging^7^. During the 6 months of active treatment, 4 (8%) of the 49 participants treated with abatacept and 17 (35%) of the 49 patients in the placebo group were diagnosed with RA; abatacept prolonged the duration to develop RA, with an HR of 0.14 (95% CI 0.04 to 0.47)^7^. Overall, the risk difference between abatacept and placebo was greater than 20% in these trials, which is a clinically relevant effect size consistent with our results in individuals with PR.

Importantly, in our study, although participants received a reduced dose during the second year, they were on active treatment for the 24-month study period, whereas in the APIPPRA trial participants were on active treatment for 1 year, and in the ARIAA trial they were on active treatment for 6 months, followed in both trials with a drug-free period of an 12 additional months. The cumulative incidence of individuals diagnosed with RA increased from the active treatment to the follow-up period with abatacept from 6% to 25% in the APIPPRA trial^8^ and from 8% to 35% in the ARIAA trial^7^. In our study, the cumulative incidence of individuals diagnosed with RA among abatacept-treated individuals increased from 0% during the first 12 months to 10% during the subsequent 12 months (ADO analysis). These findings suggest that maintaining abatacept treatment for a longer period could further reduce the risk of developing RA. Whether half or full doses of abatacept should be used, abatacept should be maintained for a longer time or other strategies should be used requires further exploration. With respect to maintenance treatment, abatacept results in individuals with RA indicate that long-term efficacy is maintained with no major safety issues^17,18^. However, these data are from a population with an overt, chronic and disabling disease, such as RA, which affects individual well-being and quality of life. By contrast, individuals with PR usually have episodic and short-lasting flares and, therefore, may be less willing to receive long-term preventive treatment. A qualitative study conducted on first-degree relatives of individuals with RA revealed that these individuals commonly have negative perceptions about taking preventive medication to reduce their risk of RA, especially because of concerns about the side effects of medications^19^. In this regard, it is important to note that, in our trial, the benefits of abatacept appeared to extend to short-term outcomes, such as greater improvement in the severity of flares and a higher frequency of remission than hydroxychloroquine. Moreover, abatacept was well tolerated. These data suggest that abatacept has an adequate risk–benefit profile that would allow its use for the preventive management of RA in individuals with PR. However, 64% (ADO analysis) of participants in the hydroxychloroquine group were not diagnosed with RA during the 2-year study period, indicating the need for better risk prediction models that may help to improve the selection of individuals with PR at risk of developing RA to reduce overexposure to pharmacological treatments^3,20^.

In contrast to abatacept, methotrexate in individuals with clinically suspected arthralgia and subclinical inflammation detected by magnetic resonance imaging^21^, a single dose of rituximab in a similar population but positive for ACPA and RF^6^, and hydroxychloroquine in a population with high levels of ACPA but without inflammatory arthralgia^22^ have not been shown to prevent the development of RA. The difference between abatacept and other DMARDs may lie in their different mechanisms of action. Abatacept (CTL4-Fc) inhibits T cell activation by binding to the costimulatory molecules CD80 and CD86 expressed on antigen-presenting cells (monocytes and B cells), leading to the inhibition of immune adaptive responses, including B cell differentiation and antibody production^23^. The preclinical phase of RA is characterized by activation and maturation of the adaptive immune response, as well as by the presence of autoantibodies whose complexity is enriched by a variety of recognized epitopes (autoantigens) and isotypes^24^. In individuals with RA, abatacept plus methotrexate reduces the titers of all isotypes of ACPAs together with a decrease in the recognition of citrullinated epitopes, suggesting a modulation of the ACPA response by abatacept; this impaired response may reduce the likelihood for RA progression in individuals at risk, such as those with PR and inflammatory arthralgia^25^. In our exploratory analysis of the effects of abatacept on monotherapy on antimodified peptide/protein antibody response in PR, we did not observe relevant differences in comparison with hydroxychloroquine. Interestingly, we observed that, in individuals with PR who did not develop RA in the long term, this maturation of the B cell response to citrullinated or carbamylated antigens was more restricted than in individuals with established RA^26,27^.

Recently, studies applying multiomic methodologies in people at risk of RA who progress to RA have shed light on the immunobiology of this transition stage^28^. ACPA^+^ individuals at risk of RA were characterized by progressive systemic inflammation and dysregulation of B and T cell subset populations during the preclinical phase, which was associated with RA progression; the authors also compared their results with those from clinical trials in which RA patients were treated with abatacept or a tumor necrosis factor inhibitor^28^. The genetic signatures of naive and central memory CD4 T cells found during progression to clinical RA were significantly modulated by treatment in abatacept responders, but not with tumor necrosis factor inhibitor^28^. Furthermore, abatacept-induced changes were observed in genes previously implicated in RA-like diseases (*CD8A*, *CD99*, *CDK4*, *CXCR3*, *FLT3LG*, *GZMM*, *LTB* and *TNFRSF14*) and in those related to the T helper 17 cell pathway^28^. Importantly, the progression to RA signatures of naive and central memory CD4 T cells was 20 times more likely to be reversed with abatacept treatment compared to tumor necrosis factor inhibitor^28^. These data suggest that T cell mechanisms relevant to clinical RA may contribute to disease pathogenesis in the preclinical phase and may provide mechanistic evidence supporting the role of abatacept in delaying the onset of clinical RA^29^.

The lack of blinding is the main limitation of our study. Although blinding is a necessary methodological safeguard of a clinical trial, for some authors blinding could have less of an impact on trials using an active comparator and those aimed at determining an intention-to-treat effect^30^, such as in our trial. However, overestimation of the treatment effect and/or underestimation of treatment harm owing to the lack of blinding could be present in this trial. No central adjudication of the clinical endpoint was performed, which would have led to a more consistent evaluation and allowed a blind evaluation. To some extent, the use of an active comparator instead of a placebo could be considered a limitation because of the higher internal validity of placebo-controlled trials. However, in addition to ethical and feasibility issues, the use of placebos does not inform real clinical practice^31^, and PR is an orphan indication for which running a randomized clinical trial is difficult. The small sample size led to imprecision in the estimates, limiting the strength of the evidence provided by our study. On the other hand, the lack of a follow-up period without active treatment precluded the evaluation of whether the beneficial effects of abatacept persisted after treatment withdrawal. A 5-year follow-up of the individuals included in this trial is currently underway to provide valuable information on whether the beneficial effects of abatacept persist over the long term. We did not include a sensitive evaluation of joints by imaging techniques such as ultrasound or magnetic resonance imaging, which would have provided a more complete picture of the clinical course of the participants throughout the study. In addition, due to this lack of imaging assessment, we did not evaluate the presence of subclinical synovitis, which is a potential prognostic factor. However, the scant literature on this topic provides inconsistent results on whether the presence of subclinical tenosynovitis and/or synovitis is a risk factor for RA progression in PR^32,33^. Finally, the lack of information on patient-reported outcomes is an issue for better delineating the risk‒benefit analysis of this intervention.

In conclusion, we showed that, among patients with early-onset PR who are seropositive for autoantibodies, treatment with abatacept reduced the risk of developing RA compared with treatment with hydroxychloroquine and was also more effective at controlling clinical symptoms, with good tolerability. Despite the limitations of our study, abatacept could be considered in clinical practice when patients with seropositive PR are managed, after a careful discussion of risks and benefits, and individual values and preferences are incorporated into our recommendation.

## Methods

The PALABA trial (effects of abatacept on the progression to RA in patients with palindromic rheumatism) was a randomized, open-label trial conducted at 14 rheumatology centers in Spain. The protocol was approved by the ethics committee of the Hospital Clínic Barcelona (Barcelona, Spain; Reference HCB/2018/0768) and the Spanish Agency of Medicines (Reference 2018 MUH/CLIN/EC). This study was conducted in accordance with the principles of the Declaration of Helsinki and Good Clinical Practices. All participants provided written informed consent before inclusion in the study. The trial was registered at ClinicalTrials.gov (NCT03669367) and EudraCT (no. 2017-004543-20). The study protocol is available as Supplementary Information.

### Participants

We included adults aged ≥18 years who were diagnosed with PR according to the modified criteria of Guerne and Weissman^34^, who had a disease duration of ≥3 months but <3 years and who were positive for RF and/or ACPA. The modification of the criteria for PR consisted of considering at least a 3-month history instead of a 6-month history of recurrent episodes (see Supplementary Box 1 for the original criteria). Individuals were excluded if they had persistent arthritis (involvement in one or more joints lasting for more than 1 week), met the criteria for other rheumatic diseases, had evidence of radiographic damage, had previously used conventional synthetic or biologic DMARDs, were receiving glucocorticoids within 1 month before study entry, had any contraindication to the study drugs, were pregnant or wished to become pregnant, or were not willing to take appropriate contraceptive measures during the study.

### Randomization and masking

Randomization codes were assigned centrally using electronic Case Report Forms at the time of patient inclusion. These randomized codes were produced by PROC PLAN of the SAS, with a 1:1 ratio of assignment between both arms, in blocks multiple of two elements and stratified by center. The center was included as a logistic stratification factor in the randomization procedure but was not included as a covariate in the main analysis following regulatory recommendations regarding the use of covariates^35^.

The participants, clinicians and outcome assessors were not blinded to the treatment assignment.

### Procedures

Participants were randomized to abatacept (125 mg subcutaneous injections weekly) or oral hydroxychloroquine (5 mg kg^−^^1^ per day) for the first 12 months of treatment; subsequently, abatacept was administered at a dosage of 125 mg every 2 weeks, and hydroxychloroquine was maintained at the same dosage. The reason for maintaining the dose for 2 years was that the progression to RA in individuals with PR is higher during the first 2–3 years^36^ after the onset of symptoms. Because in the early phase of PR the inflammatory burden is low, following one of the strategies in patients with RA for optimizing biologic therapies once remission is achieved, we decided to reduce the dose of abatacept for the second year of treatment (that is, administration every 2 weeks). Systemic glucocorticoids at an equivalent dosage higher than 7.5 mg per day of prednisone and other DMARDs were not permitted during the study. Patients who met the criteria for RA at any time throughout the study discontinued the protocol and were treated according to the clinician’s criteria.

Participants were seen at screening, baseline and every 3 months thereafter until the end of the study or at the time of treatment discontinuation or early withdrawal. Concomitant medication, general physical examinations, characteristics of the attacks as elicited from the anamnesis (that is, number, duration, intensity as evaluated using a visual analog scale, presence and perceived inflammatory joint signs, and joints involved), joint assessments and assessments of the American College of Rheumatology/European League Against Rheumatism criteria for RA were recorded at each study visit; although it was not explicitly stated in the question, it was understood that ‘intensity’ essentially referred to the degree of pain and/or functional impairment experienced by the patients. Radiographs of the hands and feet were completed at screening, except if they had been performed within the previous 6 months. Blood tests for evaluating blood counts, serum chemistry, C-reactive protein levels and erythrocyte sedimentation rates were performed at screening, at 6 months and every 6 months thereafter. Serum biomarkers were tested at baseline and at 3, 12 and 24 months.

For evaluating the effect of study treatments on AMPAs, we determined by means of appropriate enzyme-linked immunosorbent assay methods seven antigens (Supplementary Methods) with different post-translational modifications: (1) peptides bearing one post-translational modification that included citrullinated peptides derived from vimentin, α-enolase and fibrin and filaggrin (chimeric fibrin and filaggrin citrullinated peptide), and homocitrullinated peptide (chimeric fibrin and filaggrin homocitrullinated peptide); (2) peptides bearing multiple post-translational modifications that included chimeric fibrin and filaggrin citrullinated and homocitrullinated peptide and chimeric fibrin and filaggrin citrullinated and homocitrullinated and acetylated peptide; and (3) the protein antigen carbamylated fetal calf serum. We used a peptide with an unmodified basal structure, chimeric fibrin and filaggrin peptide, as a control peptide. For each antigen specificity, we determined three isotypes: IgG, IgA and IgM. The effects of abatacept and hydroxychloroquine on autoantibody levels, specificities and isotypes were evaluated at baseline and at 3, 12 and 24 months of follow-up for each patient included in the study.

### Outcomes

The primary outcome was the development of persistent arthritis (that is, lasting more than 1 week in the same joint) that fulfilled the 2010 RA classification criteria of the American College of Rheumatology/European League Against Rheumatism as evaluated by the participant clinicians during the 24 months of follow-up^37^.

Secondary outcomes included the frequency, intensity and duration of joint attacks and the proportion of patients in remission, defined as no or a single palindromic attack during at least four consecutive visits (that is, 12 months). We also evaluated the frequency of adverse events. As an exploratory outcome, we evaluated the effect of treatment on AMPA titers.

### Statistical analysis

The sample size was calculated to test for progression to RA at any time over 24 months, and the two treatment arms were compared using the chi-square test. Assuming a 2-year progression rate of at least 42% in the control group^36^ and 11% in the experimental group, a sample size of 35 patients per group (total 70) was necessary to reach a power of 80% at a nominal two-sided alpha level of 0.05, according to the nQuery validated software (nQuery version 9)^38^. No multiplicity adjustment was implemented. There is only one variable and timepoint defined as a primary endpoint; thus, there is only one confirmatory *P* value in the trial, and the rest of the *P* values are considered descriptive at their nominal value.

Primary efficacy analysis was performed using the mFAS, defined as all randomized patients who received at least one dose of the study medication. For the primary outcome, a PP analysis was also performed, which included patients from the mFAS population without major protocol deviations that might impact the main study assessments. Safety analyses were performed in the mFAS population. Missing data on progression to RA were imputed as treatment failure (failure imputation) for primary analysis, and sensitivity analysis was performed using the ADO approach.

Categorical variables are described with absolute and relative frequencies, whereas quantitative variables are described with the mean (s.d.) or the median (P25, P75) if not normally distributed.

For the primary outcome, we conducted a chi-square or Fisher’s exact test, depending on the test requirements. As a supportive analysis, the time to occurrence of RA was estimated using the Kaplan‒Meier method, comparisons were performed with the log-rank test, and HRs (95% CI) were derived from a Cox model. Data were censored at the time of the event, the last documented visit or the end of the follow-up period, whichever occurred first.

For secondary efficacy outcomes, the following strategies were used: the chi-square test or Fisher’s exact test for categorical variables, the dependent or independent *t*-test for continuous Gaussian-distributed variables and the Mann‒Whitney *U*-test for ordinal and non-Gaussian continuous data.

Continuous safety variables were analyzed using mixed models for repeated measurements, including a baseline value as a covariate, treatment effect and treatment by visit interaction. The variance‒covariance matrix employed was unstructured. The treatment effect was estimated using adjusted means (least squares mean), s.e.m. and 95% CI. Differences between treatments were estimated on the basis of the differences in the least squares mean, s.e.m. and 95% CI. The participants with at least one adverse event, globally, and by System Organ Class were compared using Fisher’s exact test.

For the analysis of the effect on AMPAs, each antigen–antibody pair was represented as a continuous variable, the Δ optical density. The Δ optical density was calculated by subtracting the optical density of each antigen–antibody pair without post-translational modifications from the corresponding antigen–antibody pair with post-translational modifications. Δ optical density measurements for each antigen–antibody pair at 0, 3, 12 and 24 months were analyzed using a parametric approach (see details below) on previously log-transformed data (GM). Between-group and within-group comparisons were evaluated using the GM ratio and geometric mean fold rise. The parametric approach consisted of a restricted maximum likelihood-based repeated measures approach (mixed models for repeated measurements). Analyses included the fixed, categorical effects for treatment, visit and the treatment-by-visit interactions. A common unstructured (co)variance structure was used to model within-patient errors. If the initial model failed to converge, the following correlation structures were tested in subsequent order until model convergence was achieved: autoregressive model of order 1 (AR[1]), Toeplitz or compound symmetry. The Kenward‒Roger approximation was used to estimate the denominator degrees of freedom^39^. GM, GM ratio and geometric mean fold rise estimates for each treatment are presented with the associated two-sided 95% CI.

We did not prespecify any subgroup analyses, including sex or gender. To our knowledge, no data suggest that clinical presentation of PR or treatment outcomes differ according to sex or gender. Therefore, sex and gender were not considered in the study design.

Statistical analysis was performed using SAS (version 9.4; SAS Institute Inc.). All the statistical tests were two-sided and were assessed at a significance level of 0.05.

### Reporting summary

Further information on research design is available in the Nature Portfolio Reporting Summary linked to this article.

## Online content

Any methods, additional references, Nature Portfolio reporting summaries, source data, extended data, supplementary information, acknowledgements, peer review information; details of author contributions and competing interests; and statements of data and code availability are available at 10.1038/s41591-026-04395-6.

## Supplementary information

Supplementary Information Supplementary Methods, Supplementary Tables 1–12 and Supplementary Box 1.

Reporting Summary

Peer Review File

Study Protocol

## Data Availability

The datasets generated and/or analyzed during this clinical study are not publicly available due to restrictions related to data. Anonymized datasets may be provided by the corresponding author upon reasonable request with a response expected within 4 weeks. This request must include a methodologically proposal and will be evaluated in accordance with General Data Protection Regulation or equivalent local legislation, ethical approvals, institutional policies and data sharing agreements.

## Code availability

The code generated during this clinical study is not publicly available due to restrictions related to data.
