# Supplementary material for: Abatacept versus hydroxychloroquine for prevention of rheumatoid arthritis in individuals with palindromic rheumatism: a randomized open-label trial
Source: Nat Med. 2026 May 14;32(7):2610–8. doi: 10.1038/s41591-026-04395-6 (PMC13375639; doi:10.1038/s41591-026-04395-6)
Supplement: Supplementary file 1 — Supplementary Methods, Supplementary Tables 1–12 and Supplementary Box 1. [file 41591_2026_4395_MOESM1_ESM.pdf]

nature medicine

Supplementary information https://doi.org/10.1038/s41591-026-04395-6

Abatacept versus hydroxychloroquine for prevention of rheumatoid arthritis in individuals with palindromic rheumatism: a randomized open-label trial

In the format provided by the authors and unedited

Supplementary information

Content

Supplementary Methods for Evaluating Anti-Modified Peptide/protein Antibodies (AMPAs) 2

Supplementary table 1. Use of anti-inflammatory therapy throughout the study period (mFAS population) 6

Supplementary table 2. Univariate logistic regression analysis of the association between treatment and occurrence of rheumatoid arthritis in individuals with palindromic rheumatism.... 7

Supplementary table 3. Multivariate logistic regression analysis of the association between treatment and occurrence of rheumatoid arthritis in individuals with palindromic rheumatism adjusting for ACPA positivity at baseline 8

Supplementary table 4. Multivariate logistic regression analysis of the association between treatment and occurrence of rheumatoid arthritis in individuals with palindromic rheumatism adjusting for the maximum duration of the flares at baseline 9

Supplementary table 5 Multivariate logistic regression analysis of the association between treatment and occurrence of rheumatoid arthritis in individuals with palindromic rheumatism adjusting for ACPA positivity and the maximum duration of the flares at baseline 10

Supplementary table 6 Proportion of patients with RA progression at month 24. Descriptive table and treatment comparisons. Imputed data and ADO approaches. mFAS & PP population sets....11

Supplementary table 7 Proportion of patients in remission. Descriptive table and treatment comparisons. mFAS/safety set. 12

Supplementary table 8 Course of duration of attacks. 13

Supplementary table 9. Effect of abatacept and hidroxychloroquine on IgG-AMPA autoantibodies in individuals with palindromic rheumatism. 14

Supplementary table 10. Effect of abatacept and hidroxychlorquine on IgA-AMPA autoantibodies in individuals with palindromic rheumatism. 16

Supplementary table 11. Effect of abatacept and hidroxychlorquine on IgM-AMPA autoantibodies in patients with palindromic rheumatism. 18

Supplementary table 12 Routine blood test. Absolute values. Inferential analysis (MMRM model). Values adjusted by baseline. 20

Supplementary box 1. Palindromic rheumatism criteria of Guerne and Weisman. 22

Supplementary Methods for Evaluating Anti-Modified Peptide/protein Antibodies (AMPAs)

MATERIALS

Peptide antigens

Peptides bearing one post-translational modification:

1. Citrullinated peptides derived from vimentin (**Vim-P55**), α-enolase (**CEP-1**) and fibrin/filaggrin (*Chimeric Fibrin Filaggrin Citrullinated Peptide*, **CFFCP**).
2. Homocitrulinated peptide (*Chimeric Fibrin Filaggrin Homocitrullinated Peptide*, **CFFHP**)

Peptides bearing multiple post-translational modifications:

3. Chimeric Fibrin Filaggrin Citrullinated Homocitrullinated Peptide (**CFFCHP**)
4. Chimeric Fibrin Filaggrin Citrullinated Homocitrullinated Acetylated Peptide (**CFFCHAP**)

Control peptide:

5. Chimeric Fibrin Filaggrin Peptide (**CFFP**)

Vim-P55: STSRSLYASSPGGVYATCitSSAVCitLCitS

CEP-1: CKIHACitEIFDSCitGNPTVEC

CFFCP: Biotinyl-PEG_2-HSTKRGHARSRPVCitG-HQCHQESTCitGRSRGRCGRSGS

CFFHP: Biotinyl-PEG_2-HSThCitRGHAhCitSRPVRG-HQCHQESThCitGRSRGRCGRSGS

CFFCHP: Biotinyl-PEG_2-HSTKRGHAhCitSRPVCitG-HQCHQESTCitGRSRGRCGRSGS

CFFCHAP: Biotinyl-PEG_2-HSTK(Ac)RGHAhCitSRPVCitG-HQCHQESTCitGRSRGRCGRSGS

CFFP: Biotinyl-PEG_2-HSTKRGHARSRPVRG-HQCHQESTRGRSRGRCGRSGS

Figure 1. Primary structure of peptide antigens. **Cit**: citrulline; **hCit**: homocitrulline; **K(Ac)**: acetyl-lysine

Protein antígen

Carbamylated fetal calf serum (**FCS-CarP**)

METHODOLOGY

Solid-Phase Peptide Synthesis

Peptides were synthesized by Solid-Phase Peptide Synthesis (SPPS) as C-terminal carboxamides on a Novasyn TGR resin (0.2 meq/g, *Novabiochem*) and following a 9-fluorenylmethoxycarbonyl/*tert*-Butyl (Fmoc/t-But) strategy. Couplings were performed by 2-(1H-7-azabenzotriazole-1-yl)-1,1,3,3-tetramethyluronium hexafluorophosphate (HATU, *Genscript*) and diisopropylethylamine (DIEA, *Merck*) activation, with three-fold molar excesses of amino acids (*Novabiochem*). The Fmoc deprotection step was performed twice with 20% piperidine (*Merck*) in dimethylformamide (DMF, *Scharlau*) for 10 min. For chimeric peptides, a fraction of each peptidyl-resin was biotinylated at the N-terminus. The biotynilation step was completed by means of the addition of N-biotinyl-NH-$(PEG)_2$-COOH (4 equivalents) dissolved in a minimal volume of DMF and in the presence of a phosphonium salt (benzotriazole-1-yloxytris(pirrolidino) phosphonium hexafluorophosphate PyBOP, 4 equivalents) (*Merck*), as well as 1-hydroxybenzotriazole (HOBt, 4 equivalents) (*Merck*) and DIEA (8 equivalents). The reaction was left overnight at room temperature. The peptides with and without biotin were cleaved from the resin by means of treatment with 94% trifluoroacetic acid (TFA, *Scharlau*) in the presence of scavengers (2.5% v/v H_2O, 2.5% v/v triisopropylsilane, 1% v/v 2-mercaptoethanol) for 5 h). The TFA was evaporated under N_2 flow. Diethyl ether (*Panreac*) was added to precipitate the crude peptides, which were isolated by centrifugation. The solids were dissolved in 10% acetic acid (AcOH, *Scharlau)* in water and lyophilized. Cyclization of peptides by forming a disulfide bridge was performed in solution. Peptides were dissolved in AcOH/H_2O (1:1, 1 mg/mL) under N_2, then hydrochloric acid (HCl, 1 M, 0.1 mL/mg) followed by iodine (I_2, 20 equiv/Acm) were added. After 4 h, I_2 was quenched by the addition of 1 M of ascorbic acid dropwise until the mixture became colourless. The mixture was then concentrated and evaporated under reduced pressure to approximately one third of the original volume. Linear and cyclic peptides were purified by HPLC on a semi-preparative scale on an Agilent Technologies 1260 Infinity chromatograph using an Agilent ZORBAX SB-C_{18} (semi-preparative RP, 9.4 × 250 mm, particle size 5 µm) (*Agilent Technologies*) at a flow rate of 2.5 mL/min and a detection wavelength of 220 nm. Pure peptides were characterized by UPLC-MS on a Waters ACQUITY UPLC (*Waters Corporation*) using the column ACQUITY UPLC BEH C_{18} (RP, 2.1 × 100 mm, particle size 1.7 µm) equipped with an UV-Vis detector and an electrospray ionization mass spectrometry (ESI-MS) Waters LCT Premier XE (*Micromass Waters*).

Protein carbamylation

FCS was carbamylated by incubating a 4mg/ml concentration with 1M of KCNO (or with 1M of KCl for the control) for 15 hours at 37ºC. After incubation, the samples were desalted by centrifugation (Amicon Ultra-0.5 centrifugal filter units, *Merck*). Carbamylation efficiency was

assessed by amino acid analysis of the hydrolysed samples in a Biochrom 30 amino acid analyser (*Biokrom, UK*) using L-Norleucine as the internal standard. The conversion of Lys to homocitrulline was determined as the fraction of the total amount of amino acids.

ELISA assays

Three different methodologies were used depending on the antigens tested:

1. Vim-P55 and CEP-1

Cabrera-Villalba et al. *Arthritis Research & Therapy* (2017) 19:141

Peptide sequences were coupled covalently to ELISA microplates (Nunc Immobilizer Amino, *Thermo Scientific*). All reagents used were form *Merck*.
Peptides were diluted to 10 µg/mL in 0.05 M carbonate/bicarbonate (pH 9.6) buffer; 100 µL of peptide solution was added to each well of microplates and incubated overnight at 4°C. Each plate contained control wells that included all reagents except the serum sample in order to estimate the background reading and control wells that included all reagents except the peptide to evaluate nonspecific reactions of sera. For blank controls, wells were coupled with 2 µg bovine serum albumin (BSA)/well. After incubation, the plates were blocked with 2% BSA in 0.05 M carbonate/bicarbonate (pH 9.6) buffer for 1 h at room temperature. Sera were diluted 50-fold in RIA buffer (1% BSA, 350 mM NaCl, 10 mM Tris-HCl, pH 7.6, 1% vol/vol Triton X-100, 0.5% wt/vol Na-deoxycholate, 0.1% SDS) supplemented with 10% fetal bovine serum; 100 µL/well were added and incubated for 1.5 h at room temperature. After washing six times with phosphate-buffered saline (PBS)/0.05% Tween-20, 100 µL/well of the anti-human secondary antibodies conjugated to peroxidase at different dilutions in RIA buffer (IgG, *DAKO* 1:1000, IgA, *Jackson ImmunoResearch* 1:2000, and IgM, *Jackson ImmunoResearch* 1:40000) were added. After incubation for 1 h at room temperature, the plates were washed six times with PBS/0.05% Tween-20 and bound antibodies were detected with o-phenylenediamine dihydrochloride (OPD; *Sigma Chemical Company*) and 0.8 µL/mL 30% hydrogen peroxide. The plates were incubated at room temperature for 30 min. The reaction was stopped with 50 µL of 2 N H_2SO_4 per well and absorbance values were measured at a wavelength of 492 nm. All sera were tested in duplicate.

2. Biotinylated peptides (CFFCP, CFFHP, CFFCHP and CFFCHAP)

García-Moreno, C et al. *International Journal of Molecular Sciences* (2021), 22: 13290

Nunc MaxiSorp microtiter plates *(Thermo Fisher Scientific*) were incubated with Neutravidin protein diluted in PBS (0.5 µg/well) overnight at 4ºC and thereafter, 1 h at 37ºC. After washing the plates, the biotinyl-peptides were diluted at 1 µg/mL in PBS and 100µL of the peptide solution was added to each well. The plates were incubated for 1 h at 37ºC. Subsequently, the plates were

blocked with 2% BSA in PBS with 0.05% Tween-20 for 30 min at 37ºC. Then, the plates were washed 3 times.

Sera were diluted 250-fold in RIA buffer supplemented with 10% fetal bovine serum and 100 µL of the dilution was added to each well. The plates were incubated for 1 h at 37ºC and then overnight at 4ºC. Afterwards, each plate was washed 3 times with PBS/0.05% Tween-20 and 100 µL of anti-human secondary antibody conjugated to peroxidase was added to each well and incubated for 1 h at 37ºC.

Three different isotypes were tested. Anti-human IgG, anti-human IgM and anti-human serum IgA secondary antibodies (*Jackson ImmunoResearch*) were diluted in RIA buffer at 1:4000; 1:2000 and 1:10000, respectively. After washing the plates, detection of bound antibodies was carried out using SigmaFast (*Sigma-Aldrich, St. Louis, MO, USA*), with *o*-phenylenediamine dihydrochloride (OPD) as a substrate. Reaction was stopped with 50 µL of 2N H_2SO_4 and plates were read at 492 nm. All sera were tested in duplicate.

Reactivity to the non-citrullinated, non-homocitrullinated and non-acetylated chimeric peptides (unmodified basal structure) was subtracted from the reactivity to the citrullinated, homocitrullinated and acetylated peptides to ensure the reactivity shown was specific to the post-translational modifications.

3. Carbamylated protein (FCS-CarP)

Castellanos-Moreira, R. et al Annals of the Rheumatic Diseases (2020), 79:587.

Anti-FCS were determined by ELISA. All samples were assayed in separate plates (Nunc MaxiSorp) coated with FCS and non-modified protein as antigens overnight at a concentration of 10 µg/mL of carbonate-bicarbonate buffer (0.1 M pH 9.6). After washing the plates, the wells were blocked with 1% BSA in PBS with 0.05% Tween-20 for 6 hours at 4°C. A volume of 50 µL of the diluted serum samples (1:50 in PBS-1% BSA-0.05% Tween) was added to each well and the plates were incubated overnight at 4°C. Afterwards, the plates were washed 3 times with PBS/0.05% Tween-20 and 50 µL of anti-human secondary antibody conjugated to alkaline phosphatase (*Jackson ImmunoResearch*) was added to each well and incubated for 3h and 30min at 4°C. Anti-human IgG, anti-human IgA and anti-human IgM secondary antibodies were diluted in PBS-1% BSA-0.05% Tween at 1:5000; 1:1000 and 1:5000 dilution, respectively. After washing the plates, detection of bound antibodies was carried out using Sigma Fast (*Sigma-Aldrich*), with p-nitrophenyl phosphate as substrate. Reaction was stopped with 50 µL of NaOH 3N and plates were read at 405nm. All the sera were tested in duplicate. Reactivity of the non-modified protein was substrated from the reactivity to carbamylated protein to ensure the reactivity was specific to the homocitrulline modification.

Supplementary table 1. Use of anti-inflammatory therapy throughout the study period (mFAS population)

Treatment	Abatacept (N=34)	Hydroxychloroquine (N=36)
Colchicine, n (%)	1 (2.9%)	0 (0.0%)
Oral glucocorticoids, n (%)	7 (20.6%)	19 (52.8%)
NSAIDs, n (%)	21 (61.8%)	25 (69.4%)

NSAIDs, nonsteroidal anti-inflammatory drugs

Supplementary table 2. Univariate logistic regression analysis of the association between treatment and occurrence of rheumatoid arthritis in individuals with palindromic rheumatism

Variable	Tested	Odds Ratio Estimate	Lower 95% Confidence Limit for Odds Ratio	Upper 95% Confidence Limit for Odds Ratio	Wald Chi-Square	Pr > Chi-Square
Treatment	Abatacept vs Hydroxychloroquine	0.259	0.09	0.746	6.2621	0.0123

Supplementary table 3. Multivariate logistic regression analysis of the association between treatment and occurrence of rheumatoid arthritis in individuals with palindromic rheumatism adjusting for ACPA positivity at baseline

Variable	Tested	Odds Ratio Estimate	Lower 95% Confidence Limit for Odds Ratio	Upper 95% Confidence Limit for Odds Ratio	Wald Chi-Square	Pr > Chi-Square
Treatment	Abatacept vs Hydroxychloroquine	0.285	0.097	0.839	5.1956	0.0226
ACPA positivity	Yes vs No	2.248	0.234	21.57	0.4929	0.4827

ACPA, Anti-Citrullinated Protein Antibodies

Supplementary table 4. Multivariate logistic regression analysis of the association between treatment and occurrence of rheumatoid arthritis in individuals with palindromic rheumatism adjusting for the maximum duration of the flares at baseline

Variable	Tested	Odds Ratio Estimate	Lower 95% Confidence Limit for Odds Ratio	Upper 95% Confidence Limit for Odds Ratio	Wald Chi-Square	Pr > Chi-Square
Treatment	Abatacept vs Hydroxychloroquine	0.202	0.065	0.628	7.6494	0.0057
Maximum_duration of flares	≥72 hours vs <72 hours	0.296	0.084	1.043	3.5904	0.0581

Supplementary table 5 Multivariate logistic regression analysis of the association between treatment and occurrence of rheumatoid arthritis in individuals with palindromic rheumatism adjusting for ACPA positivity and the maximum duration of the flares at baseline

Variable	Tested	Odds Ratio Estimate	Lower 95% Confidence Limit for Odds Ratio	Upper 95% Confidence Limit for Odds Ratio	Wald Chi-Square	Pr > Chi-Square
Treatment	Abatacept vs Hydroxychloroquine	0.22	0.069	0.706	6.4842	0.0109
ACPA positivity	Yes vs No	1.826	0.187	17.808	0.2687	0.6042
Maximum_duration of flares	≥72 hours vs <72 hours	0.308	0.087	1.096	3.3055	0.069

ACPA, Anti-Citrullinated Protein Antibodies

Supplementary table 6 Proportion of patients with RA progression at month 24. Descriptive table and treatment comparisons. Imputed data and ADO approaches. mFAS & PP population sets.

Population and time/ **Missing data approach**	**Category**	**Hydroxychloroquine (n=36) n (%)**	**Abatacept (n=34) n (%)**	**TOTAL (n=70) n (%)**	**p value**
mFAS at 24 months					
Failure imputation	Yes	18 (50.0%)	7 (20.6%)	25 (35.7%)	0.010*
	No	18 (50.0%)	27 (79.4%)	45 (64.3%)	
	TOTAL	36 (100.0%)	34 (100.0%)	70 (100.0%)	
ADO	Yes	10 (35.7%)	3 (10.0%)	13 (22.4%)	0.019*
	No	18 (64.3%)	27 (90.0%)	45 (77.6%)	
	TOTAL	28 (100.0%)	30 (100.0%)	58 (100.0%)	
PP at 24 months					
Failure imputation	Yes	16 (47.1%)	7 (21.2%)	23 (34.3%)	0.026*
	No	18 (52.9%)	26 (78.8%)	44 (65.7%)	
	TOTAL	34 (100.0%)	33 (100.0%)	67 (100.0%)	
ADO	Yes	10 (35.7%)	3 (10.3%)	13 (22.8%)	0.022*
	No	18 (64.3%)	26 (89.7%)	44 (77.2%)	
	TOTAL	28 (100.0%)	29 (100.0%)	57 (100.0%)	
mFAS at 12 months					
Failure imputation	Yes	13 (36.1%)	3 (8.8%)	16 (22.9%)	0.007*
	No	23 (63.9%)	31 (91.2%)	54 (77.1%)	
	TOTAL	36 (100.0%)	34 (100.0%)	70 (100.0%)	
ADO	Yes	8 (25.8%)	0 (0.0%)	8 (13.1%)	0.005#
	No	23 (74.2%)	31 (100.0%)	54 (87.1%)	
	TOTAL	31 (100.0%)	31 (100.0%)	62 (100.0%)	
PP at 12 months					
Failure imputation	Yes	11 (32.4%)	3 (9.1%)	14 (20.9%)	0.019*
	No	23 (67.6%)	30 (90.9%)	53 (79.1%)	
	TOTAL	34 (100.0%)	33 (100.0%)	67 (100.0%)	
ADO	Yes	8 (25.8%)	0 (0.0%)	8 (13.1%)	0.005#
	No	23 (74.2%)	30 (100.0%)	53 (86.9%)	
	TOTAL	31 (100.0%)	30 (100.0%)	61 (100.0%)	

* Two-sided Chi square test without multiplicity adjustment

Two-sided Fisher's exact test without multiplicity adjustment

ADO, available data only; mFAS, modified full analysis set; PP, per-protocol population.

Supplementary table 7 Proportion of patients in remission. Descriptive table and treatment comparisons. mFAS/safety set.

Variable Time	Category	Hydroxychloroquine (n=36) n (%)	Abatacept (n=34) n (%)	Total (n=70) n (%)	p value*
0-12 months	No	27 (77.1%)	20 (58.8%)	47 (68.1%)	0.126
	Yes	8 (22.9%)	14 (41.2%)	22 (31.9%)	
	Total	35 (100.0%)	34 (100.0%)	69 (100.0%)	
0-24 months	No	27 (77.1%)	15 (44.1%)	42 (60.9%)	0.007
	Yes	8 (22.9%)	19 (55.9%)	27 (39.1%)	
	Total	35 (100.0%)	34 (100.0%)	69 (100.0%)	

*Two-sided Fisher exact test without multiplicity adjustment

mFAS, modified full analysis set

Supplementary table 8 Course of duration of attacks.

Period	Variable	Category	Hydroxy-chloroquine (n=706) n (%)	Abatacept (n=793) n (%)	Total (n=1499) n (%)	p value*
6 months prior to the start of treatment	Duration	<24 hours	21 (10.6%)	12 (9.2%)	33 (10.1%)	-
		>=24 hours - <48 hours	57 (28.8%)	58 (44.6%)	115 (35.1%)	
		>=48 hours - <72 hours	59 (29.8%)	45 (34.6%)	104 (31.7%)	
		>=72 hours - <1 week	61 (30.8%)	15 (11.5%)	76 (23.2%)	
		TOTAL	198 (100.0%)	130 (100.0%)	328 (100.0%)	
0-12 months	Duration	<24 hours	62 (21.4%)	77 (32.1%)	139 (26.2%)	0.0002
		>=24 hours - <48 hours	68 (23.4%)	67 (27.9%)	135 (25.5%)	
		>=48 hours - <72 hours	85 (29.3%)	52 (21.7%)	137 (25.8%)	
		>=72 hours - <1 week	50 (17.2%)	43 (17.9%)	93 (17.5%)	
		>= 1 week	25 (8.6%)	1 (0.4%)	26 (4.9%)	
		TOTAL	290 (100.0%)	240 (100.0%)	530 (100.0%)	
0-24 months	Duration	<24 hours	84 (16.6%)	164 (31.2%)	248 (24.1%)	<.0001
		>=24 hours - <48 hours	169 (33.4%)	151 (28.8%)	320 (31.0%)	
		>=48 hours - <72 hours	138 (27.3%)	108 (20.6%)	246 (23.9%)	
		>=72 hours - <1 week	84 (16.6%)	84 (16.0%)	168 (16.3%)	
		>= 1 week	31 (6.1%)	18 (3.4%)	49 (4.8%)	
		TOTAL	506 (100.0%)	525 (100.0%)	1031 (100.0%)	

*Two-sided Mann-Whitney test without multiplicity adjustment

Supplementary table 9. Effect of abatacept and hidroxychloroquine on IgG-AMPA autoantibodies in individuals with palindromic rheumatism.

Parameters	Visit	Statistics[#]	Hydroxychloroquine (n=36)	Abatacept (n=34)	GM ratio [95%CI]; p-value[$]
CEP 1-IgG	0 months	GM (SE)[95%CI]	0.43 (1.21) [0.29 - 0.63]	0.36 (1.21) [0.25 - 0.53]	-
	3 months	GM (SE)[95%CI]	0.46 (1.22) [0.31 - 0.68]	0.39 (1.22) [0.26 - 0.59]	0.85 [0.49 - 1.5]; 0.5790
		GMFR [95%CI]; p-value	1.07 [0.89 - 1.28]; 0.4832	1.07 [0.9 - 1.29]; 0.4325	
	12 months	GM (SE)[95%CI]	0.44 (1.21) [0.3 - 0.65]	0.35 (1.22) [0.24 - 0.51]	0.79 [0.46 - 1.37]; 0.3968
		GMFR [95%CI]; p-value	1.02 [0.83 - 1.26]; 0.8358	0.95 [0.78 - 1.17]; 0.6504	
	24 months	GM (SE)[95%CI]	0.39 (1.24) [0.26 - 0.61]	0.32 (1.24) [0.21 - 0.49]	0.82 [0.45 - 1.51]; 0.5218
		GMFR [95%CI]; p-value	0.92 [0.7 - 1.21]; 0.5305	0.89 [0.69 - 1.15]; 0.3738	
CFFCHAP-IgG	0 months	GM (SE)[95%CI]	0.19 (1.64) [0.07 - 0.49]	0.1 (1.65) [0.04 - 0.27]	-
	3 months	GM (SE)[95%CI]	0.17 (1.66) [0.06 - 0.46]	0.1 (1.67) [0.04 - 0.28]	0.6 [0.14 - 2.53]; 0.4782
		GMFR [95%CI]; p-value	0.9 [0.64 - 1.27]; 0.5408	0.99 [0.7 - 1.4]; 0.9611	
	12 months	GM (SE)[95%CI]	0.2 (1.72) [0.07 - 0.58]	0.09 (1.72) [0.03 - 0.26]	0.44 [0.09 - 2.04]; 0.2884
		GMFR [95%CI]; p-value	1.06 [0.61 - 1.85]; 0.8262	0.86 [0.5 - 1.47]; 0.5813	
	24 months	GM (SE)[95%CI]	0.16 (1.72) [0.05 - 0.46]	0.07 (1.72) [0.02 - 0.2]	0.42 [0.09 - 1.96]; 0.2653
		GMFR [95%CI]; p-value	0.84 [0.56 - 1.28]; 0.4247	0.66 [0.45 - 0.96]; 0.0326*	
CFFCHP-IgG	0 months	GM (SE)[95%CI]	0.12 (1.78) [0.04 - 0.38]	0.08 (1.79) [0.02 - 0.25]	-
	3 months	GM (SE)[95%CI]	0.11 (1.77) [0.04 - 0.35]	0.08 (1.79) [0.03 - 0.27]	0.76 [0.15 - 3.84]; 0.7325
		GMFR [95%CI]; p-value	0.91 [0.69 - 1.19]; 0.4718	1.07 [0.81 - 1.41]; 0.6099	
	12 months	GM (SE)[95%CI]	0.14 (1.76) [0.04 - 0.43]	0.11 (1.77) [0.04 - 0.34]	0.79 [0.16 - 3.92]; 0.7693
		GMFR [95%CI]; p-value	1.14 [0.64 - 2.05]; 0.6533	1.41 [0.8 - 2.5]; 0.2307	
	24 months	GM (SE)[95%CI]	0.11 (1.78) [0.03 - 0.35]	0.08 (1.79) [0.02 - 0.25]	0.7 [0.14 - 3.6]; 0.6661
		GMFR [95%CI]; p-value	0.91 [0.62 - 1.33]; 0.6268	1 [0.7 - 1.43]; 0.9971	
CFFCP-IgG	0 months	GM (SE)[95%CI]	0.13 (1.76) [0.04 - 0.39]	0.07 (1.77) [0.02 - 0.22]	-
	3 months	GM (SE)[95%CI]	0.09 (1.76) [0.03 - 0.29]	0.09 (1.78) [0.03 - 0.29]	0.97 [0.19 - 4.86]; 0.9656
		GMFR [95%CI]; p-value	0.73 [0.51 - 1.06]; 0.0946	1.31 [0.91 - 1.9]; 0.1471	
	12 months	GM (SE)[95%CI]	0.11 (1.76) [0.03 - 0.33]	0.11 (1.77) [0.04 - 0.35]	1.05 [0.21 - 5.23]; 0.9498
		GMFR [95%CI]; p-value	0.84 [0.52 - 1.36]; 0.4693	1.64 [1.02 - 2.64]; 0.0415*	
	24 months	GM (SE)[95%CI]	0.12 (1.77) [0.04 - 0.37]	0.09 (1.77) [0.03 - 0.29]	0.77 [0.15 - 3.88]; 0.7490
		GMFR [95%CI]; p-value	0.93 [0.56 - 1.54]; 0.7720	1.33 [0.84 - 2.12]; 0.2238	
CFFHP-IgG	0 months	GM (SE)[95%CI]	0.02 (1.56) [0.01 - 0.04]	0.01 (1.57) [0.01 - 0.03]	-
	3 months	GM (SE)[95%CI]	0.03 (1.55) [0.01 - 0.06]	0.01 (1.56) [0 - 0.02]	0.4 [0.11 - 1.38]; 0.1427
		GMFR [95%CI]; p-value	1.45 [0.89 - 2.36]; 0.1371	0.8 [0.49 - 1.32]; 0.3791	
	12 months	GM (SE)[95%CI]	0.02 (1.58) [0.01 - 0.05]	0.01 (1.58) [0 - 0.03]	0.56 [0.15 - 2.02]; 0.3690
		GMFR [95%CI]; p-value	1.16 [0.53 - 2.55]; 0.6996	0.91 [0.42 - 1.96]; 0.8075	
	24 months	GM (SE)[95%CI]	0.03 (1.55) [0.01 - 0.07]	0 (1.53) [0 - 0.01]	0.18 [0.05 - 0.59]; 0.0058*
		GMFR [95%CI]; p-value	1.57 [0.75 - 3.26]; 0.2260	0.38 [0.19 - 0.77]; 0.0077*	
FCS CarP-IgG	0 months	GM (SE)[95%CI]	0.2 (1.5) [0.09 - 0.44]	0.3 (1.5) [0.13 - 0.67]	-
	3 months	GM (SE)[95%CI]	0.16 (1.52) [0.07 - 0.37]	0.28 (1.53) [0.12 - 0.65]	1.72 [0.52 - 5.67]; 0.3651
		GMFR [95%CI]; p-value	0.82 [0.51 - 1.32]; 0.4044	0.93 [0.58 - 1.51]; 0.7684	
	12 months	GM (SE)[95%CI]	0.23 (1.47) [0.11 - 0.49]	0.26 (1.47) [0.12 - 0.56]	1.13 [0.38 - 3.36]; 0.8267
		GMFR [95%CI]; p-value	1.16 [0.7 - 1.91]; 0.5594	0.86 [0.53 - 1.41]; 0.5540	
	24 months	GM (SE)[95%CI]	0.22 (1.47) [0.1 - 0.48]	0.27 (1.46) [0.13 - 0.57]	1.2 [0.41 - 3.54]; 0.7322
		GMFR [95%CI]; p-value	1.13 [0.7 - 1.83]; 0.6124	0.9 [0.58 - 1.4]; 0.6368	
Vim P55-IgG	0 months	GM (SE)[95%CI]	0.27 (1.17) [0.2 - 0.37]	0.29 (1.17) [0.21 - 0.39]	-
	3 months	GM (SE)[95%CI]	0.3 (1.16) [0.22 - 0.4]	0.26 (1.17) [0.19 - 0.35]	0.85 [0.56 - 1.31]; 0.4604
		GMFR [95%CI]; p-value	1.1 [0.91 - 1.34]; 0.3268	0.89 [0.73 - 1.09]; 0.2529	
	12 months	GM (SE)[95%CI]	0.29 (1.18) [0.21 - 0.4]	0.25 (1.18) [0.18 - 0.35]	0.87 [0.55 - 1.38]; 0.5451
		GMFR [95%CI]; p-value	1.05 [0.86 - 1.29]; 0.5934	0.87 [0.72 - 1.06]; 0.1629	
	24 months	GM (SE)[95%CI]	0.26 (1.17) [0.19 - 0.35]	0.21 (1.17) [0.16 - 0.29]	0.82 [0.53 - 1.28]; 0.3827
		GMFR [95%CI]; p-value	0.95 [0.78 - 1.16]; 0.6156	0.74 [0.62 - 0.9]; 0.0023*	

[#] GM, Geometric mean; GMFR, Geometric mean fold rise, calculated relative to baseline (month 0) for each treatment.

[$] GM ratio between treatment arms, Hydroxychloroquine arm as reference.
* $P < 0.05$ (two-sided test without multiplicity adjustment)
AMPA, Anti-Modified Peptide/protein Antibodies

Autoantibodies included 1) citrullinated peptides derived from vimentin (Vim-P55), α-enolase (CEP-1) and fibrin/filaggrin (Chimeric Fibrin Filaggrin Citrullinated Peptide, CFFCP), and homocitrulinated peptide (Chimeric Fibrin Filaggrin Homocitrullinated Peptide [CFFHP]); 2) peptides bearing multiple posttranslational modifications which included Chimeric Fibrin Filaggrin Citrullinated Homocitrullinated Peptide (CFFCHP) and Chimeric Fibrin Filaggrin Citrullinated Homocitrullinated Acetylated Peptide (CFFCHAP); and 3) the protein antigen Carbamylated fetal calf serum (FCS-CarP).

Supplementary table 10. Effect of abatacept and hidroxychlorquine on IgA-AMPA autoantibodies in individuals with palindromic rheumatism.

Parameters	Visit	Statistics#	Hydroxychloroquine (n=36)	Abatacept (n=34)	GM ratio [95%CI]; p-value$
CEP 1-IgA	0 months	GM (SE)[95%CI]	0.21 (1.13) [0.17 - 0.27]	0.15 (1.13) [0.12 - 0.19]	-
	3 months	GM (SE)[95%CI]	0.2 (1.12) [0.16 - 0.25]	0.14 (1.13) [0.11 - 0.18]	0.72 [0.51 - 1]; 0.0471
		GMFR [95%CI]; p-value	0.94 [0.88 - 1]; 0.0457	0.95 [0.9 - 1.01]; 0.1273	
	12 months	GM (SE)[95%CI]	0.21 (1.13) [0.16 - 0.26]	0.15 (1.14) [0.12 - 0.19]	0.73 [0.51 - 1.04]; 0.0844
		GMFR [95%CI]; p-value	0.97 [0.84 - 1.12]; 0.6739	1.01 [0.88 - 1.15]; 0.9233	
	24 months	GM (SE)[95%CI]	0.21 (1.15) [0.16 - 0.28]	0.17 (1.14) [0.13 - 0.22]	0.81 [0.55 - 1.18]; 0.2670
		GMFR [95%CI]; p-value	0.99 [0.82 - 1.2]; 0.9393	1.14 [0.96 - 1.36]; 0.1428	
CFFCHAP-IgA	0 months	GM (SE)[95%CI]	0.02 (1.55) [0.01 - 0.06]	0.02 (1.55) [0.01 - 0.05]	-
	3 months	GM (SE)[95%CI]	0.01 (1.61) [0.01 - 0.04]	0.01 (1.62) [0 - 0.03]	0.83 [0.21 - 3.18]; 0.7776
		GMFR [95%CI]; p-value	0.59 [0.33 - 1.05]; 0.0715	0.6 [0.34 - 1.09]; 0.0928	
	12 months	GM (SE)[95%CI]	0.02 (1.65) [0.01 - 0.05]	0.01 (1.65) [0 - 0.03]	0.53 [0.13 - 2.2]; 0.3794
		GMFR [95%CI]; p-value	0.79 [0.37 - 1.69]; 0.5346	0.53 [0.25 - 1.11]; 0.0912	
	24 months	GM (SE)[95%CI]	0.02 (1.71) [0.01 - 0.07]	0.01 (1.66) [0 - 0.03]	0.49 [0.11 - 2.15]; 0.3409
		GMFR [95%CI]; p-value	1 [0.36 - 2.75]; 0.9999	0.61 [0.24 - 1.57]; 0.3038	
CFFCHP-IgA	0 months	GM (SE)[95%CI]	0.03 (1.54) [0.01 - 0.07]	0.02 (1.54) [0.01 - 0.04]	-
	3 months	GM (SE)[95%CI]	0.01 (1.62) [0.01 - 0.04]	0.01 (1.63) [0 - 0.03]	0.64 [0.16 - 2.5]; 0.5146
		GMFR [95%CI]; p-value	0.52 [0.31 - 0.9]; 0.0205	0.56 [0.32 - 0.96]; 0.0364	
	12 months	GM (SE)[95%CI]	0.01 (1.64) [0.01 - 0.04]	0.01 (1.65) [0 - 0.02]	0.56 [0.14 - 2.28]; 0.4119
		GMFR [95%CI]; p-value	0.52 [0.26 - 1.06]; 0.0722	0.49 [0.24 - 0.97]; 0.0424	
	24 months	GM (SE)[95%CI]	0.01 (1.75) [0 - 0.04]	0.01 (1.71) [0 - 0.03]	0.71 [0.15 - 3.33]; 0.6578
		GMFR [95%CI]; p-value	0.48 [0.16 - 1.38]; 0.1684	0.56 [0.21 - 1.52]; 0.2504	
CFFCP-IgA	0 months	GM (SE)[95%CI]	0.01 (1.58) [0 - 0.03]	0.01 (1.58) [0 - 0.01]	-
	3 months	GM (SE)[95%CI]	0.01 (1.58) [0 - 0.03]	0.01 (1.6) [0 - 0.02]	0.61 [0.17 - 2.26]; 0.4565
		GMFR [95%CI]; p-value	0.94 [0.52 - 1.72]; 0.8421	1.12 [0.61 - 2.05]; 0.7199	
	12 months	GM (SE)[95%CI]	0.01 (1.62) [0 - 0.03]	0.01 (1.62) [0 - 0.03]	0.86 [0.22 - 3.33]; 0.8206
		GMFR [95%CI]; p-value	1.13 [0.55 - 2.31]; 0.7344	1.87 [0.93 - 3.77]; 0.0782	
	24 months	GM (SE)[95%CI]	0.01 (1.75) [0 - 0.03]	0.01 (1.69) [0 - 0.03]	0.97 [0.21 - 4.44]; 0.9630
		GMFR [95%CI]; p-value	0.85 [0.34 - 2.14]; 0.7241	1.58 [0.68 - 3.67]; 0.2783	
CFFHP-IgA	0 months	GM (SE)[95%CI]	0.03 (1.44) [0.02 - 0.07]	0.01 (1.44) [0 - 0.02]	-
	3 months	GM (SE)[95%CI]	0.02 (1.52) [0.01 - 0.04]	0.01 (1.54) [0 - 0.01]	0.34 [0.1 - 1.12]; 0.0751
		GMFR [95%CI]; p-value	0.47 [0.26 - 0.85]; 0.0130	0.54 [0.3 - 0.98]; 0.0444	
	12 months	GM (SE)[95%CI]	0.02 (1.51) [0.01 - 0.04]	0.01 (1.51) [0 - 0.01]	0.36 [0.11 - 1.14]; 0.0820
		GMFR [95%CI]; p-value	0.49 [0.26 - 0.92]; 0.0274	0.61 [0.33 - 1.12]; 0.1076	
	24 months	GM (SE)[95%CI]	0.02 (1.54) [0.01 - 0.04]	0 (1.52) [0 - 0.01]	0.28 [0.08 - 0.93]; 0.0374
		GMFR [95%CI]; p-value	0.48 [0.24 - 0.96]; 0.0373	0.46 [0.25 - 0.87]; 0.0174	
FCS CarP-IgA	0 months	GM (SE)[95%CI]	0.03 (1.46) [0.02 - 0.07]	0.02 (1.46) [0.01 - 0.04]	-
	3 months	GM (SE)[95%CI]	0.03 (1.47) [0.01 - 0.07]	0.03 (1.48) [0.01 - 0.06]	0.85 [0.28 - 2.55]; 0.7713
		GMFR [95%CI]; p-value	0.93 [0.51 - 1.69]; 0.8095	1.32 [0.73 - 2.39]; 0.3603	
	12 months	GM (SE)[95%CI]	0.03 (1.47) [0.01 - 0.07]	0.03 (1.47) [0.01 - 0.06]	0.99 [0.34 - 2.93]; 0.9893
		GMFR [95%CI]; p-value	0.91 [0.5 - 1.67]; 0.7691	1.51 [0.83 - 2.74]; 0.1725	
	24 months	GM (SE)[95%CI]	0.03 (1.55) [0.01 - 0.08]	0.04 (1.51) [0.02 - 0.08]	1.04 [0.31 - 3.45]; 0.9450
		GMFR [95%CI]; p-value	1.05 [0.49 - 2.24]; 0.8954	1.82 [0.9 - 3.67]; 0.0925	
Vim P55-IgA	0 months	GM (SE)[95%CI]	0.19 (1.11) [0.15 - 0.23]	0.17 (1.11) [0.14 - 0.21]	-
	3 months	GM (SE)[95%CI]	0.17 (1.1) [0.14 - 0.21]	0.16 (1.1) [0.13 - 0.19]	0.92 [0.7 - 1.2]; 0.5239
		GMFR [95%CI]; p-value	0.92 [0.84 - 1]; 0.0450	0.93 [0.86 - 1.02]; 0.1145	
	12 months	GM (SE)[95%CI]	0.17 (1.11) [0.14 - 0.21]	0.17 (1.11) [0.14 - 0.21]	1.02 [0.75 - 1.37]; 0.9073
		GMFR [95%CI]; p-value	0.9 [0.82 - 0.98]; 0.0199	1.02 [0.93 - 1.11]; 0.6985	
	24 months	GM (SE)[95%CI]	0.17 (1.12) [0.14 - 0.22]	0.16 (1.12) [0.13 - 0.2]	0.91 [0.67 - 1.25]; 0.5635
		GMFR [95%CI]; p-value	0.92 [0.82 - 1.04]; 0.1742	0.94 [0.84 - 1.04]; 0.2246	

GM, Geometric mean; GMFR, Geometric mean fold rise, calculated relative to baseline (month 0) for each treatment.

$ GM ratio between treatment arms, Hydroxychloroquine arm as reference.
* P < 0.05 (two-sided test without multiplicity adjustment)

AMPA, Anti-Modified Peptide/protein Antibodies
Autoantibodies included 1) citrullinated peptides derived from vimentin (Vim-P55), α-enolase (CEP-1) and fibrin/filaggrin (Chimeric Fibrin Filaggrin Citrullinated Peptide, CFFCP), and homocitrulinated peptide (Chimeric Fibrin Filaggrin Homocitrullinated Peptide [CFFHP]); 2) peptides bearing multiple posttranslational modifications which included Chimeric Fibrin Filaggrin Citrullinated Homocitrullinated Peptide (CFFCHP) and Chimeric Fibrin Filaggrin Citrullinated Homocitrullinated Acetylated Peptide (CFFCHAP); and 3) the protein antigen Carbamylated fetal calf serum (FCS-CarP).

Supplementary table 11. Effect of abatacept and hidroxychlorquine on IgM-AMPA autoantibodies in patients with palindromic rheumatism.

Parameters	Visit	Statistics[#]	Hydroxychloroquine (n=36)	Abatacept (n=34)	GM ratio [95%CI]; p-value[$]
CEP 1-IgM	0 months	GM (SE)[95%CI]	0.07 (1.1) [0.06 - 0.08]	0.07 (1.11) [0.06 - 0.08]	-
	3 months	GM (SE)[95%CI]	0.06 (1.11) [0.05 - 0.08]	0.07 (1.11) [0.06 - 0.09]	1.1 [0.83 - 1.47]; 0.5050
		GMFR [95%CI]; p-value	0.93 [0.82 - 1.06]; 0.3002	1.03 [0.91 - 1.18]; 0.6080	
	12 months	GM (SE)[95%CI]	0.07 (1.13) [0.05 - 0.09]	0.07 (1.13) [0.06 - 0.09]	1.09 [0.78 - 1.52]; 0.6273
		GMFR [95%CI]; p-value	0.98 [0.79 - 1.22]; 0.8312	1.07 [0.86 - 1.33]; 0.5484	
	24 months	GM (SE)[95%CI]	0.07 (1.13) [0.05 - 0.08]	0.08 (1.12) [0.07 - 0.11]	1.29 [0.92 - 1.81]; 0.1410
		GMFR [95%CI]; p-value	0.94 [0.76 - 1.16]; 0.5483	1.22 [1 - 1.48]; 0.0490	
CFFCHAP-IgM	0 months	GM (SE)[95%CI]	0.05 (1.55) [0.02 - 0.12]	0.04 (1.56) [0.02 - 0.11]	-
	3 months	GM (SE)[95%CI]	0.04 (1.57) [0.01 - 0.09]	0.05 (1.58) [0.02 - 0.13]	1.51 [0.42 - 5.41]; 0.5219
		GMFR [95%CI]; p-value	0.7 [0.41 - 1.19]; 0.1799	1.23 [0.72 - 2.1]; 0.4499	
	12 months	GM (SE)[95%CI]	0.04 (1.62) [0.02 - 0.11]	0.04 (1.63) [0.02 - 0.11]	1.03 [0.26 - 4.05]; 0.9654
		GMFR [95%CI]; p-value	0.81 [0.41 - 1.6]; 0.5333	0.97 [0.49 - 1.92]; 0.9318	
	24 months	GM (SE)[95%CI]	0.07 (1.62) [0.03 - 0.18]	0.05 (1.58) [0.02 - 0.12]	0.69 [0.18 - 2.59]; 0.5740
		GMFR [95%CI]; p-value	1.38 [0.6 - 3.16]; 0.4479	1.11 [0.51 - 2.39]; 0.7964	
CFFCHP-IgM	0 months	GM (SE)[95%CI]	0.02 (1.61) [0.01 - 0.05]	0.02 (1.61) [0.01 - 0.04]	-
	3 months	GM (SE)[95%CI]	0.01 (1.66) [0 - 0.02]	0.02 (1.68) [0.01 - 0.05]	1.91 [0.45 - 8.1]; 0.3742
		GMFR [95%CI]; p-value	0.5 [0.25 - 1]; 0.0493	1.03 [0.51 - 2.1]; 0.9265	
	12 months	GM (SE)[95%CI]	0.02 (1.65) [0.01 - 0.06]	0.01 (1.66) [0.01 - 0.04]	0.63 [0.15 - 2.6]; 0.5131
		GMFR [95%CI]; p-value	1.31 [0.63 - 2.73]; 0.4620	0.9 [0.44 - 1.84]; 0.7640	
	24 months	GM (SE)[95%CI]	0.02 (1.74) [0.01 - 0.06]	0.01 (1.7) [0 - 0.04]	0.59 [0.13 - 2.73]; 0.4937
		GMFR [95%CI]; p-value	1.16 [0.45 - 2.98]; 0.7555	0.75 [0.31 - 1.78]; 0.5034	
CFFCP-IgM	0 months	GM (SE)[95%CI]	0.01 (1.61) [0 - 0.02]	0.01 (1.62) [0 - 0.02]	-
	3 months	GM (SE)[95%CI]	0 (1.62) [0 - 0.01]	0.01 (1.63) [0 - 0.02]	1.75 [0.44 - 6.9]; 0.4191
		GMFR [95%CI]; p-value	0.43 [0.26 - 0.74]; 0.0024	1.23 [0.72 - 2.11]; 0.4379	
	12 months	GM (SE)[95%CI]	0.02 (1.62) [0.01 - 0.04]	0.01 (1.61) [0 - 0.02]	0.43 [0.11 - 1.65]; 0.2129
		GMFR [95%CI]; p-value	1.56 [0.76 - 3.21]; 0.2201	1.08 [0.54 - 2.18]; 0.8251	
	24 months	GM (SE)[95%CI]	0.02 (1.67) [0.01 - 0.05]	0.01 (1.63) [0 - 0.02]	0.5 [0.12 - 2.07]; 0.3355
		GMFR [95%CI]; p-value	1.72 [0.74 - 4.04]; 0.2058	1.4 [0.64 - 3.06]; 0.3865	
CFFHP-IgM	0 months	GM (SE)[95%CI]	0.01 (1.52) [0 - 0.02]	0.01 (1.53) [0 - 0.01]	-
	3 months	GM (SE)[95%CI]	0.01 (1.54) [0 - 0.01]	0.01 (1.55) [0 - 0.02]	1.08 [0.31 - 3.7]; 0.9052
		GMFR [95%CI]; p-value	0.59 [0.35 - 0.99]; 0.0444	1.24 [0.73 - 2.1]; 0.4222	
	12 months	GM (SE)[95%CI]	0.01 (1.53) [0 - 0.02]	0.01 (1.53) [0 - 0.01]	0.56 [0.17 - 1.86]; 0.3390
		GMFR [95%CI]; p-value	0.97 [0.52 - 1.81]; 0.9155	1.06 [0.57 - 1.96]; 0.8498	
	24 months	GM (SE)[95%CI]	0.01 (1.55) [0 - 0.02]	0.01 (1.52) [0 - 0.02]	1.03 [0.31 - 3.49]; 0.9558
		GMFR [95%CI]; p-value	0.86 [0.39 - 1.9]; 0.7079	1.75 [0.83 - 3.66]; 0.1381	
FCS CarP-IgM	0 months	GM (SE)[95%CI]	0.12 (1.38) [0.06 - 0.23]	0.07 (1.38) [0.04 - 0.13]	-
	3 months	GM (SE)[95%CI]	0.09 (1.41) [0.04 - 0.18]	0.06 (1.42) [0.03 - 0.12]	0.7 [0.26 - 1.88]; 0.4737
		GMFR [95%CI]; p-value	0.74 [0.59 - 0.94]; 0.0152	0.89 [0.7 - 1.13]; 0.3306	
	12 months	GM (SE)[95%CI]	0.09 (1.39) [0.05 - 0.18]	0.07 (1.39) [0.04 - 0.14]	0.8 [0.31 - 2.02]; 0.6250
		GMFR [95%CI]; p-value	0.78 [0.55 - 1.1]; 0.1551	1.06 [0.76 - 1.48]; 0.7359	
	24 months	GM (SE)[95%CI]	0.07 (1.44) [0.03 - 0.14]	0.08 (1.42) [0.04 - 0.16]	1.13 [0.41 - 3.11]; 0.8039
		GMFR [95%CI]; p-value	0.58 [0.37 - 0.91]; 0.0195	1.12 [0.75 - 1.69]; 0.5752	
Vim P55-IgM	0 months	GM (SE)[95%CI]	0.07 (1.08) [0.06 - 0.08]	0.07 (1.08) [0.06 - 0.09]	-
	3 months	GM (SE)[95%CI]	0.06 (1.08) [0.05 - 0.07]	0.07 (1.08) [0.06 - 0.08]	1.13 [0.92 - 1.4]; 0.2368
		GMFR [95%CI]; p-value	0.93 [0.86 - 1.01]; 0.0718	0.97 [0.9 - 1.05]; 0.4468	
	12 months	GM (SE)[95%CI]	0.06 (1.08) [0.05 - 0.07]	0.07 (1.08) [0.06 - 0.08]	1.15 [0.92 - 1.44]; 0.2249
		GMFR [95%CI]; p-value	0.93 [0.84 - 1.03]; 0.1870	0.98 [0.89 - 1.09]; 0.7573	
	24 months	GM (SE)[95%CI]	0.06 (1.08) [0.05 - 0.07]	0.07 (1.08) [0.06 - 0.08]	1.14 [0.91 - 1.43]; 0.2660
		GMFR [95%CI]; p-value	0.95 [0.84 - 1.06]; 0.3637	0.99 [0.89 - 1.1]; 0.8415	

[#] GM, Geometric mean; GMFR, Geometric mean fold rise, calculated relative to baseline (month 0) for each treatment.

[$] GM ratio between treatment arms, Hydroxychloroquine arm as reference.
* $P < 0.05$ (two-sided test without multiplicity adjustment)

AMPA, Anti-Modified Peptide/protein Antibodies

Autoantibodies included 1) citrullinated peptides derived from vimentin (Vim-P55), α-enolase (CEP-1) and fibrin/filaggrin (Chimeric Fibrin Filaggrin Citrullinated Peptide, CFFCP), and homocitrulinated peptide (Chimeric Fibrin Filaggrin Homocitrullinated Peptide [CFFHP]); 2) peptides bearing multiple posttranslational modifications which included Chimeric Fibrin Filaggrin Citrullinated Homocitrullinated Peptide (CFFCHP) and Chimeric Fibrin Filaggrin Citrullinated Homocitrullinated Acetylated Peptide (CFFCHAP); and 3) the protein antigen Carbamylated fetal calf serum (FCS-CarP).

Supplementary table 12 Routine blood test. Absolute values. Inferential analysis (MMRM model). Values adjusted by baseline.

Variable Visit	Hydroxychloroquine* n, Mean (SD), [95% CI] at Baseline; Adjusted Mean (SEM) [95% CI] by time	Abatacept* n, Mean (SD), [95% CI] at Baseline; Adjusted Mean (SEM) [95% CI] by time	Treatment differences. Adjusted Mean (SEM) [95% CI]	p value treatment effect
Alkaline phosphatase (U/L).				
Screening	34, 70.61, (15.83) [65.09;76.13]	34, 72.81, (15.50) [67.40;78.22]	-	-
6 months	69.98 (2.96) [64.08;75.89]	74.86 (2.92) [69.03;80.69]	4.88 (4.15) [-3.42;13.18]	0.245
12 months	68.40 (2.09) [64.22;72.57]	75.03 (2.00) [71.04;79.03]	6.64 (2.89) [0.85;12.42]	0.025
18 months	67.97 (1.93) [64.11;71.82]	74.40 (1.80) [70.81;78.00]	6.44 (2.64) [1.16;11.71]	0.018
24 months	71.44 (2.80) [65.84;77.04]	75.03 (2.52) [70.00;80.07]	3.59 (3.77) [-3.95;11.13]	0.345
ALT Alanine aminotransferase (U/L).				
Screening	36, 22.63, (20.23) [15.78;29.47]	33, 18.64, (8.25) [15.71;21.56]	-	-
6 months	19.86 (1.92) [16.03;23.69]	20.08 (1.96) [16.15;24.00]	0.22 (2.75) [-5.28;5.71]	0.938
12 months	20.71 (2.43) [15.85;25.57]	19.53 (2.30) [14.94;24.11]	-1.19 (3.35) [-7.88;5.51]	0.725
18 months	20.80 (1.81) [17.18;24.42]	17.82 (1.75) [14.32;21.32]	-2.98 (2.53) [-8.02;2.07]	0.243
24 months	23.63 (2.67) [18.29;28.96]	20.68 (2.47) [15.75;25.61]	-2.95 (3.64) [-10.2;4.33]	0.422
AST Aspartate aminotransferase (U/L).				
Screening	35, 20.23, (5.49) [18.35;22.12]	32, 20.36, (6.55) [18.00;22.72]	-	-
6 months	21.81 (1.54) [18.73;24.89]	21.01 (1.58) [17.86;24.17]	-0.80 (2.20) [-5.21;3.61]	0.718
12 months	21.98 (1.27) [19.44;24.51]	21.65 (1.25) [19.14;24.16]	-0.32 (1.78) [-3.89;3.24]	0.856
18 months	21.12 (1.02) [19.07;23.17]	20.03 (1.01) [18.00;22.06]	-1.09 (1.44) [-3.98;1.79]	0.451
24 months	23.26 (1.42) [20.42;26.09]	22.09 (1.37) [19.34;24.83]	-1.17 (1.97) [-5.12;2.78]	0.556
Cholesterol total (mg/dL).				
Screening	33, 202.7, (33.42) [190.8;214.5]	32, 200.5, (26.89) [190.8;210.2]	-	-
6 months	187.0 (3.73) [179.5;194.5]	204.7 (3.75) [197.2;212.2]	17.65 (5.30) [7.06;28.25]	0.001
12 months	180.2 (5.00) [170.2;190.2]	196.5 (4.59) [187.4;205.7]	16.38 (6.80) [2.78;29.98]	0.019
18 months	181.6 (4.56) [172.4;190.7]	201.7 (4.13) [193.4;210.0]	20.15 (6.16) [7.83;32.47]	0.002
24 months	189.1 (5.46) [178.2;200.0]	212.2 (5.07) [202.1;222.4]	23.09 (7.45) [8.18;38.00]	0.003
C-Reactive protein (CRP) (mg/dL).				
Screening	36, 0.61, (0.55) [0.42;0.80]	34, 0.53, (0.54) [0.34;0.72]	-	-
6 months	0.50 (0.11) [0.29;0.71]	0.56 (0.11) [0.34;0.78]	0.06 (0.15) [-0.24;0.36]	0.692
12 months	0.85 (0.24) [0.37;1.32]	0.63 (0.22) [0.19;1.07]	-0.22 (0.32) [-0.87;0.43]	0.504
18 months	0.53 (0.21) [0.10;0.96]	0.69 (0.20) [0.29;1.09]	0.16 (0.29) [-0.43;0.75]	0.587
24 months	0.41 (0.14) [0.13;0.69]	0.67 (0.12) [0.42;0.92]	0.26 (0.19) [-0.11;0.63]	0.167
Creatinine (mg/dL).				
Screening	35, 0.76, (0.23) [0.69;0.84]	33, 0.74, (0.17) [0.68;0.80]	-	-
6 months	0.77 (0.02) [0.73;0.81]	0.79 (0.02) [0.75;0.83]	0.02 (0.03) [-0.03;0.08]	0.440
12 months	0.75 (0.02) [0.72;0.78]	0.78 (0.01) [0.75;0.81]	0.03 (0.02) [-0.02;0.07]	0.219
18 months	0.75 (0.02) [0.71;0.78]	0.74 (0.02) [0.71;0.77]	-0.00 (0.02) [-0.05;0.04]	0.857
24 months	0.78 (0.02) [0.74;0.82]	0.78 (0.02) [0.74;0.82]	0.00 (0.03) [-0.05;0.06]	0.941
Erythrocyte Sedimentation Rate (ESR) (mm/hour).				
Screening	36, 19.86, (14.40) [14.99;24.73]	34, 23.03, (18.31) [16.64;29.42]	-	-
6 months	15.25 (1.52) [12.22;18.29]	15.83 (1.55) [12.74;18.92]	0.58 (2.17) [-3.75;4.90]	0.791
12 months	14.50 (2.35) [9.81;19.19]	15.10 (2.16) [10.79;19.41]	0.60 (3.19) [-5.77;6.98]	0.851
18 months	17.12 (2.16) [12.80;21.44]	17.80 (2.04) [13.72;21.88]	0.68 (2.98) [-5.27;6.62]	0.820
24 months	17.46 (3.58) [10.31;24.61]	18.08 (3.25) [11.59;24.57]	0.61 (4.83) [-9.04;10.27]	0.900
Hemoglobin (g/L).				
Screening	36, 136.1, (12.00) [132.0;140.1]	34, 136.8, (14.08) [131.9;141.7]	-	-
6 months	134.7 (1.48) [131.8;137.7]	138.1 (1.51) [135.1;141.1]	3.35 (2.11) [-0.87;7.57]	0.118
12 months	134.7 (1.51) [131.7;137.7]	138.6 (1.43) [135.8;141.5]	3.93 (2.08) [-0.23;8.09]	0.064
18 months	135.5 (1.47) [132.6;138.5]	137.5 (1.41) [134.7;140.4]	2.00 (2.04) [-2.08;6.07]	0.331
24 months	134.7 (1.76) [131.2;138.2]	137.9 (1.63) [134.7;141.2]	3.22 (2.40) [-1.57;8.02]	0.184
Lymphocytes (x10^9/L).				
Screening	36, 1.85, (0.45) [1.70;2.00]	34, 1.87, (0.78) [1.60;2.14]	-	-
6 months	1.79 (0.10) [1.59;2.00]	2.10 (0.11) [1.89;2.31]	0.31 (0.15) [0.01;0.60]	0.041
12 months	1.72 (0.35) [1.02;2.42]	2.57 (0.32) [1.93;3.21]	0.85 (0.47) [-0.09;1.80]	0.077

Variable Visit	Hydroxychloroquine* n, Mean (SD), [95% CI] at Baseline; Adjusted Mean (SEM) [95% CI] by time	Abatacept* n, Mean (SD), [95% CI] at Baseline; Adjusted Mean (SEM) [95% CI] by time	Treatment differences. Adjusted Mean (SEM) [95% CI]	p value treatment effect
18 months	1.64 (0.09) [1.47;1.81]	2.16 (0.08) [1.99;2.32]	0.51 (0.12) [0.28;0.75]	<0.001
24 months	1.61 (0.10) [1.42;1.81]	2.07 (0.09) [1.89;2.25]	0.46 (0.13) [0.20;0.72]	<0.001
Neutrophils (x10^9/L).				
Screening	36, 4.83, (1.89) [4.19;5.47]	34, 4.75, (1.52) [4.22;5.28]	-	-
6 months	4.10 (0.18) [3.75;4.46]	4.40 (0.18) [4.04;4.76]	0.30 (0.25) [-0.21;0.80]	0.247
12 months	4.40 (0.24) [3.91;4.88]	4.47 (0.23) [4.01;4.93]	0.08 (0.33) [-0.59;0.75]	0.819
18 months	4.24 (0.24) [3.76;4.71]	4.45 (0.23) [3.99;4.90]	0.21 (0.33) [-0.45;0.87]	0.525
24 months	4.91 (0.30) [4.31;5.51]	4.51 (0.27) [3.98;5.05]	-0.40 (0.40) [-1.20;0.40]	0.323
Platelet count (x10^9/L).				
Screening	36, 267.5, (49.85) [250.7;284.4]	34, 272.3, (56.91) [252.4;292.2]	-	-
6 months	260.1 (5.74) [248.7;271.6]	259.6 (5.83) [248.0;271.3]	-0.47 (8.18) [-16.8;15.87]	0.954
12 months	274.0 (8.09) [257.9;290.2]	266.5 (7.59) [251.3;281.6]	-7.53 (11.09) [-29.7;14.62]	0.500
18 months	249.2 (7.29) [234.7;263.8]	267.7 (6.91) [253.9;281.5]	18.50 (10.05) [-1.57;38.57]	0.070
24 months	270.2 (7.48) [255.2;285.1]	276.0 (6.96) [262.1;289.9]	5.81 (10.22) [-14.6;26.22]	0.572
Triglycerides (mg/dL).				
Screening	32, 111.0, (68.08) [86.50;135.6]	32, 118.0, (53.82) [98.64;137.4]	-	-
6 months	113.0 (12.84) [87.29;138.7]	130.5 (12.16) [106.1;154.9]	17.50 (17.67) [-17.9;52.89]	0.326
12 months	121.3 (12.18) [96.92;145.7]	113.1 (11.32) [90.41;135.8]	-8.25 (16.63) [-41.6;25.06]	0.622
18 months	93.89 (10.47) [72.92;114.9]	116.7 (9.00) [98.63;134.7]	22.78 (13.81) [-4.88;50.43]	0.105
24 months	99.11 (12.90) [73.27;124.9]	127.9 (11.51) [104.8;150.9]	28.75 (17.28) [-5.86;63.37]	0.102
Uric acid (mg/dL).				
Screening	32, 4.56, (1.18) [4.13;4.98]	33, 4.37, (1.21) [3.94;4.80]	-	-
6 months	4.49 (0.13) [4.22;4.75]	4.60 (0.12) [4.35;4.84]	0.11 (0.18) [-0.25;0.47]	0.551
12 months	4.54 (0.25) [4.03;5.04]	4.11 (0.23) [3.65;4.58]	-0.42 (0.34) [-1.11;0.26]	0.222
18 months	4.50 (0.12) [4.25;4.75]	4.43 (0.11) [4.21;4.65]	-0.07 (0.16) [-0.40;0.26]	0.684
24 months	4.27 (0.14) [3.99;4.56]	4.93 (0.12) [4.69;5.18]	0.66 (0.19) [0.28;1.04]	<.001 (@)
White blood cell count (Leukocytes) (x10^9/L).				
Screening	36, 7.61, (2.13) [6.89;8.33]	34, 7.20, (2.23) [6.43;7.98]	-	-
6 months	6.58 (0.26) [6.07;7.09]	7.29 (0.26) [6.77;7.81]	0.71 (0.37) [-0.02;1.44]	0.056
12 months	6.61 (0.29) [6.03;7.20]	7.58 (0.28) [7.03;8.13]	0.96 (0.40) [0.16;1.76]	0.019
18 months	6.46 (0.27) [5.93;7.00]	7.67 (0.26) [7.16;8.18]	1.21 (0.37) [0.46;1.95]	0.002
24 months	7.07 (0.37) [6.34;7.80]	7.56 (0.33) [6.91;8.21]	0.49 (0.49) [-0.49;1.47]	0.323

*Mean ± SD at baseline. Baseline adjusted least squares means (95% CI) at other visits.

CI, confidence interval; MMRM, mixed models for repeated measurements; SD, standard deviation; SEM, standard error of the mean.

Supplementary box 1. Palindromic rheumatism criteria of Guerne and Weisman.

- Six-month history of brief, sudden onset and recurrent episodes of monoarthritis or, rarely, polyarthritis or soft tissue inflammation
- Direct observation of one attack by a physician
- Three or more joints involved in different attacks
- Absence of erosions on radiographs
- Exclusion of other arthritides

Reference: Guerne, P. A. & Weisman, M. H. Palindromic rheumatism: part of or apart from the spectrum of rheumatoid arthritis. Am. J. Med. 93, 451–460 (1992).
