# Supplementary material for: Abatacept versus hydroxychloroquine for prevention of rheumatoid arthritis in individuals with palindromic rheumatism: a randomized open-label trial
Source: Nat Med. 2026 May 14;32(7):2610–8. doi: 10.1038/s41591-026-04395-6 (PMC13375639; doi:10.1038/s41591-026-04395-6)
Supplement: Supplementary file 4 — Study Protocol [file 41591_2026_4395_MOESM4_ESM.pdf]

1. TITLE OF THE STUDY

EFFECTS OF ABATACEPT ON THE PROGRESSION TO RHEUMATOID ARTHRITIS IN PATIENTS WITH PALINDROMIC RHEUMATISM (PALABA).

Version 3.0 , protocol date: 14-03-2022

EudraCTnº: 2017-004543-20

Short title/Acronym: PALABA

Sponsor: Fundació Clínic per a la Recerca Biomèdica

Chief Investigator: Raimon Sanmarti, Arthritis Unit. Rheumatology Department. Hospital Clínic de Barcelona. Barcelona, Spain

Co-Investigator: Isabel Haro. Peptide Synthesis and Biomedical Applications Unit, Institute of Advanced Chemistry (IQAC-CSIC) of Catalonia, Barcelona, Spain.

SITEs OF STUDY

Spain Study Multicentre Site (See protocol appendix sites of study)

CRO-
Clinical Trials Unit (CTU)
Hospital Clínic Barcelona
Project manager Contact acruceta@clinic.ub.es
Tel + 93 227 5400 ext 9838

Monitor contact:
Eva bonfill. Mail: bonfill@clinic.cat
Tel 932275400 ext 4198
Claudia Martell . Mail: martelli@clinic.cat Tel 932275400 ext 2336

STATISTICIAN
Data Management and Statistics
Medical Statistics core facility
IDIBAPS - Hospital Clinic Barcelona
183, Mallorca Street. Floor -1. Office #60. 08036 Barcelona
http://www.idibaps.org/core-facilities/11/medical-statistics
Bioestadistica@idibaps.org
Phone: 34932275400;4197 / +34 932 279 328

Project Statistician:
José Rios: jrios@clinic.cat
Gema Domenech: gdomene1@clinic.cat

Project Data manager:
Sandra Serrano
saserran@clinic.cat

Immunoassays (ELISA tests)
IQAC-CSIC
Dra. Isabel Haro
Contact: isabel.haro@iqac.csic.es

2- STUDY SUMMARY SYNOPSIS

TITLE	**EFFECTS OF ABATACEPT ON THE PROGRESSION TO RHEUMATOID ARTHRITIS IN PATIENTS WITH PALINDROMIC RHEUMATISM.**
SHORT TITLE	PALABA
PROTOCOL VERSION/DATE	Protocol V2.0. date 22-12-2020
METHODOLOGY	Type of study: multicenter open-label randomized controlled clinical trial
DURATION	65 months (enrollment period: 41 months; open randomized period: 24 months)
OBJECTIVES	The main objective of this trial is to test the hypothesis that abatacept can reduce the progression of rheumatoid arthritis in ACPA+ or RF+ patients with palindromic rheumatism compared with patients treated with hydroxychloroquine. This effect may be attributed to a modification of the maturation of the B cell response to citrullinated peptides in patients with PR.
PHASE of THE TRIAL	Phase IV study
NUMBER OF PATIENTS	70 patients (35 treated with abatacept and 35 with hydroxychloroquine)
SITE /CENTERS	Multicentre. Hospital sites in Spain
MAIN INCLUSSION INCLUSION CRITERIA	patients with PR according to Guerne and Weissman modified criteria (18) and with:<br>- Disease evolution > 3 months and < 36 months<br>- ACPA positivity proven by ELISA test or chemiluminescence (CCP2) and/or Rheumatoid factor positivity (ELISA, nephelometry or chemiluminescence).<br>- Greater than 18 years of age
EXCLUSSION CRITERIA	- Persistent arthritis: (involvement in one or more joints > 1 week)<br>- Criteria of other rheumatic diseases (RA, SLE, etc.).<br>- Evidence of radiographic damage (join erosions).<br>- Absence of ACPA or RF<br>- Contraindication or intolerance to study drugs (abatacept or hydroxychloroquine)<br>- Steroid treatment one month before study entry<br>- Previous antitrheumatic therapy with synthetic DMARDS (methotrexate, leflunomide, sulfasalazine, cyclosporine, antimalarials..) or biological DMARDs.

	- Pregnant women or who want to be pregnant during the study
MAIN OUTCOME MEASURE	Achievement of classification criteria of rheumatoid arthritis (EULAR/ACR 2010) at any time during the follow-up (up to 24 months)
SECONDARY OUCOMES	Number and intensity of joint attacks<br>Adverse events<br>Effects on serum ACPA and anti-CarP antibodies
STATISTICS	Modified Full Analysis Set (mFAS) and Per Protocol Population (PP)

3. GLOSSARY OF TERMS AND ABBREVIATIONS

AE	Adverse Event
ALT	Alanine Transaminase
ANC	Absolute Neutrophil Count
AR	Adverse Reaction
ASR	Annual Safety Report
AST	Aspartate Aminotransferase
CA	Competent Authority
CDAI	Clinical disease activity index
CF	Consent Form
CI	Chief Investigator
CIOMS	Council for International Organizations of Medical Sciences
CRF	Case Report Form
CRO	Contract Research Organisation
CTA	Clinical Trial Authorisation
CTIMP	Clinical Trial of Investigational Medicinal Product
DMEC	Data Monitoring Committee
EC	European Commission
EMEA	European Medicines Agency
EMR	Experimental Medicine and Rheumatology
ESR	Erythrocyte Sedimentation Rate
EU	European Union
EUCTD	European Clinical Trials Directive
EudraCT	European Union Drug Regulating Authorities Clinical Trials
EudraVIGILANCE	European Union Drug Regulating Authorities Pharmacovigilance
FACIT	Functional Assessment of Chronic Illness Therapy
FBC	Full Blood Count
GCP	Good Clinical Practice
GH	General Health
GMP	Good Manufacturing Practice
IB	Investigator Brochure
IMP	Investigational Medicinal Product
IMPD	Investigational Medicinal Product Dossier
ISRCTN	International Standard Randomised Controlled Trial Number
JRO	Joint Research and Development Office
LFT	Liver Function Tests
MA	Marketing Authorisation
Main REC	Main Research Ethics Committee
MHRA	Medicines and Healthcare products Regulatory Agency
mm^3	Cubic Millimeter
MS	Member State
MTX	Methotrexate
NIHR	National Institute of Health Research
NRES	National Research Ethics Service
OMERACT	Outcome Measures in Rheumatoid Arthritis Clinical Trials
Participant	An individual who takes part in a clinical trial
p.o.	Per Os (by mouth)
PI	Principle Investigator
PIS	Patient Information Sheet

QC	Quality Control
QMUL	Queen Mary University of London
QP	Qualified Person for release of trial drug
RA	Rheumatoid Arthritis
RCT	Randomised Controlled Trial
REC	Research Ethics Committee
RF	Rheumatoid Factor
RTX	Rituximab
SAE	Serious Adverse Event
SAR	Serious Adverse Reaction
SC	Subcutaneous
SDV	Source Document Verification
SmPC	Summary of Product Characteristics
SOP	Standard Operating Procedure
SSA	Site Specific Assessment
SUSAR	Suspected Unexpected Serious Adverse Reaction
TJC	Tender Joint Count
TMG	Trial Management Group
TSC	Trial Steering Committee

4. INDEX

1. TITLE OF THE STUDY **1**
2- STUDY SUMMARY SYNOPSIS **3**
3. GLOSSARY OF TERMS AND ABBREVIATIONS **5**
4. INDEX **7**
5. INTRODUCTION **8**
5.1 BACKGROUND: 8
5.2 INVESTIGATIONAL MEDICINAL PRODUCT 9
6.TRIAL OBJECTIVES AND DESIGN **12**
6.1.TRIAL OBJECTIVE 12
6.2 ENDPOINTS 12
6.2.1 Main outcome measure: *12*
6.2.2 Secondary outcomes: *12*
6.3 TRIAL DESIGN: 13
6.4 STUDY SCHEME DIAGRAM 14
7. SUBJECT SELECTION **14**
7.1 NUMBER OF SUBJECTS. 14
7.2 INCLUSION CRITERIA: 14
7.3 EXCLUSION CRITERIA 15
7.4. CRITERIA FOR EARLY WITHDRAWAL 16
8.STUDY PROCEDURES **16**
8.1 INFORMED CONSENT PROCEDURES 16
8.2.SCREENING (VISIT 1) 17
8.3 BASELINE VISIT (VISIT NUMBER 2) AND FOLLOW-UP 17
8.4 VISIT NUMBERS (VISIT 3) 18
8.5 VISITS NUMBERS (VISIT 4, 8, AND EARLY WITHDRAWAL/STOP TREATMENT) 18
8.6 VISITS NUMBERS (VISIT5,7, 9) 19
8.7 VISITS NUMBERS (VISIT6 AND 10) 19
8.8 STUDY VISIT SCHEDULE 20
8.9 END OF STUDY TRIAL DEFINITION 21
9.STATISTICAL ANALYSIS: **21**
10. ADVERSE EVENT REPORTING **28**
11. DATA HANDLING AND RECORD KEEPING **37**
11.1 CONFIDENTIALITY 37
11.2 CASE REPORD FORM 37
11.3 COMPLIANCE 37
11.4 SERIOUS BREACHES IN GPC OR THE TRIAL PROTOCOL 37
11.5 PUBLICATION POLICY 38
11.6 FUNDING 38
THE SPONSOR HAS RECEIVED A GRANT FROM BMS FOR THE DEVELOPMENT OF THE STUDY. **38**
12. REFERENCES **38**

5. INTRODUCTION

5.1 BACKGROUND:

Palindromic rheumatism (PR) is a clinical entity characterized by intermittent arthritis lasting a few days that tends to recur and, in up to 50-60% of patients, progresses to rheumatoid arthritis (RA)(1). Approximately 15% of RA patients initially had PR (2). This high risk of RA progression is observed during the first years of evolution of PR (3). Several prognostic factors for RA in these patients have been identified, such as gender (female), involvement of the small joints of the hands, and serum autoantibodies such as rheumatoid factor (RF) or anticitrullinated peptide antibodies (ACPA)(4-5). After our first description of the high prevalence of ACPA in PR (6), this association has been confirmed in different populations (5,7,8). ACPA have been described as a biomarker of RA progression in RP patients (5), although a significant proportion of ACPA+ PR patients, even when titres are high, do not develop RA in the long term (9). Why some patients with PR progress to RA and others do not is unclear. Recently, our group demonstrated that the ACPA repertoire is restricted, with less citrullinated peptide specificities and less isotype usage than found in established RA (10), as occurs in the preclinical phase of RA (11) or in family members of patients with RA (12). It is unclear whether PR patients with a less restricted ACPA repertoire in the early phases of the disease are less prone to progress to RA.

There is no specific treatment for PR that prevents progression to persistent arthritis or RA. Gold salts and antimalarial drugs have shown efficacy in clinical symptoms, with a lower frequency of attacks, and even remission, in patients with PR treated with these antirheumatic drugs (1,13). Most rheumatologists use antimalarials (hydroxychloroquine) as the first option to treat PR. No studies have been made using

methotrexate, leflunomide or sulfasalazine (1). In a Canadian study, antimalarials increased the time to progression to RA in patients with PR (14).

5.2 INVESTIGATIONAL MEDICINAL PRODUCT

Abatacept is a biological drug approved for RA (15). Its efficacy and safety in different RA populations (methotrexate naïve, methotrexate failure and TNF blocker failure has been well documented in both clinical trials and observational studies (15). Recent findings strongly suggest it is more effective in ACPA+ RA patients (16) and, according to the mechanism of action, can reverse the ACPA repertoire, with less citrullinated peptide reactivities in ACPA+ RA patients with RA, a phenomenon that might be associated with a better clinical response (17).

TREATMENT ARMS:

A) Arm1: abatacept monotherapy (subcutaneous route).: 125 mg solution for injection in pre-filled syringe or pre-filled pen ClickJect (which are interchangeable and bioequivalent).

In the first year (0-12 months) at a a dose of 125 mg per week (full dose) and in the second year (12-24 months) at a dose of 125 mg every other week (q2w) (optimized dose)

B) Arm 2: oral hydroxychloroquine (5 mg/Kg/day) monotherapy for 2 years (0-48 months)

ABATACEPT (Orencia®)

Pharmacotherapeutic group: selective immunosupressants

ATC code: LOAA24

Store in a refrigerator (2°C - 8°C). Do not freeze.

Contraindications: Hypersensitivity to the active substance or to any of the excipients and

Severe and uncontrolled infections such as sepsis and opportunistic infections:

Adverse Reactions:

- very common: Upper respiratory tract infection (including tracheitis, nasopharyngitis, and sinusitis)

-common : Lower respiratory tract infection (including bronchitis), urinary tract infection, herpes infections (including herpes simplex, oral herpes, and herpes zoster), pneumonia, influenza. Hedache, rasch dizziness, hypertension,cough, hepatobiliar disorders

Doses up to 50 mg/kg have been administered without apparent toxic effect. In case of overdose, it is recommended that the patient be monitored for any signs or symptoms of adverse reactions and appropriate symptomatic treatment instituted.

Ther are complet IMPD In the Investigator file to consult

Abatacept will be distributed to each Hospital Pharmacy Service labeled according to the current legislation by Logista Pharma Company

ORAL HYDROXICHLOROQUINE

Pharmacotherapeutic group: antimalarial and treatment for auto-immune diseases

Store at room temperature up to 86 degrees F (30 degrees C)

Contraindications: Hypersensivity to the active substance or to other aminoquinolones

Adverse Reactions:

- very common: Nausea, stomach cramps, loss of appetite, diarrhoea, dizziness, or headache

- Comon: arm/leg/back pain, slow/fast/irregular heartbeat, symptoms of heart failure, hair loss/color change, mental/mood changes, dark urine, yellowing eyes/skin

Symptoms of overdose may include fainting, slow/fast/irregular heartbeat, extreme excitability, slow/shallow breathing, seizures, loss of consciousness.

There are complet IMPD In the Investigator file to consult

Hydroxicloroquine drug is administered by the usual assistential route and the researchers themselves will be responsible for maintaining the traceability of this drug.

6.TRIAL OBJECTIVES AND DESIGN

6.1.TRIAL OBJECTIVE

The main objective of this trial is to test the hypothesis that abatacept can reduce the progression of RA in ACPA+ patients PR compared with patients treated with hydroxychloroquine. This effect may be attributed to a modification of the maturation of the B cell response to citrullinated peptides in patients with PR.

Other secondary objectives are the clinical efficacy: frequency and intensity of joint attacks. Adverse events and Effects on ACPA titres

6.2 ENDPOINTS

6.2.1 Main outcome measure:

development of persistent arthritis fulfilling criteria of RA according to EULAR/ACR 2010 classification criteria at any time during follow-up. Patients who develop persistent arthritis (RA criteria) will be classified as non-responders.

6.2.2 Secondary outcomes:

- Clinical efficacy: frequency and intensity of joint attacks
- Adverse events
- Effects on ACPA titres, fine specificities and isotypes: Citrullinated peptides derived from fibrin, vimentin and alpha-enolase and IgG, IgM and IgA will be used. ACPA fine specificities will be measured by home-made ELISA tests according to the procedure recently described by our research group (Cabrera-Villalba S et al. Arthritis Res Ther, 2017; 19(1):141). All tests will be carried out

at baseline, and at 3, 12 and 24 months of follow-up. Serial measurements of serum RF are not considered

- Effects on anti-CarP antibodies (home-made ELISA): chemically carbamylated fetal calf serum or fibrinogen proteins will be prepared and used as antigens according to the procedures described by Shi et al. *PNAS USA* 2011, 108: 17372-7 and Scinocca et al .*J. Rheumatol.* 2014, 41: 270-9). Doubly modified (homocitrullinated and citrullinated) peptides derived from several proteins present and the inflamed synovium, recently developed in our group (unpublished results), will also be used as antígens.Anti-CarP will be measured at baseline and, in antiCarP positive patients, at 3, 12 and 24 months of follow-up.

6.3 TRIAL DESIGN:

This is a multi-centre , Phase IV ,open –label randomized controlled clinical trial comparing the efficacy of abatacept versus hydroxychloroquine on clinical symptoms and progression to RA in FR+ and/or ACPA+ PR patients.

Patients will be recruited in different hospitals in Spain.(multicentre) .Patients who meet the inclusion/exclussion criteria will be randomized assigned to one of the two arms

-Arm 1: abatacept monotherapy (subcutaneous route). In the first year (0-12 months) at a a dose of 125 mg per week (full dose) and in the second year (12-24 months) at a dose of 125 mg every other week (q2w) (optimized dose)

-Arm 2: oral hydroxychloroquine (5 mg/Kg/day) monotherapy for 2 years (0-48 months)

Study duration:65 months

.

6.4 STUDY SCHEME DIAGRAM

7. SUBJECT SELECTION

7.1 NUMBER OF SUBJECTS.

70 patients with Palindromic Rheumatism criteria of Guerne- Weisman (35 treated with abatacept and 35 with hydroxychloroquine)

7.2 INCLUSION CRITERIA:

Patients with PR according to Guerne and Weissman modified criteria (18) and with:

- Disease evolution > 3 months and < 36 months
- ACPA positivity proven by ELISA test or chemiluminescence (CCP2) and/or Rheumatoid factor positivity (ELISA, nephelometry or chemiluminescence).
- Greater than 18 years of age

Palindromic Rheumatism criteria of Guerne & Weisman (1992) (18)

ƒ. Six-month history of brief-sudden-onset and recurrent episodes of monoarthritis or, rarely, polyarthritis or of soft tissue inflammation

ƒ. Direct observation of one attack by a physician

ƒ. Three or more joints involved in different attacks

ƒ. Absence of erosions on radiographs

ƒ. Exclusion of other arthritides

We use modified Palindromic Rheumatism criteria of Guerne and Weissman for this study:

- Disease evolution less than 6 month is permitted (but not less than 3 months).
- All patients included should be seropositive for RF and/or ACPA.

7.3 EXCLUSION CRITERIA

- Persistent arthritis: (involvement in one or more joints > 1 week)
- Criteria of other rheumatic diseases (RA, SLE, etc.).
- Evidence of radiographic damage (join erosions).
- Absence of ACPA or RF
- Contraindication or intolerance to study drugs (abatacept or hydroxychloroquine)
- Steroid treatment one month before study entry
- Previous antitrheumatic therapy with synthetic DMARDS (methotrexate, leflunomide, sulfasalazine, cyclosporine, antimalarials..) or biological DMARDs
- pregnant women, who wish to be pregnant during the study or who do not take birth control precautions during the study
-

7.4. CRITERIA FOR EARLY WITHDRAWAL

Participants may be withdrawn from trial treatment if they are intolerant to the therapeutic product, experience toxicity related side-effects or inter-current illness necessitating cessation of the therapy at any time throughout the trial at the physician's discretion.

Subjects may withdraw consent for any reason at any time without prejudice to their normal care. Patients withdrawing from the study will continue to be monitored and managed within their routine clinic by their named consultant. Withdrawn trial subjects will not be replaced. Withdrawal of consent may be regarded as a withdrawal from trial medication or any other components which form part of the Informed Consent Form signed by the participant.

At the time of withdrawal, a full efficacy and safety evaluation should be performed if patient consents. Treatment cessation and reason for discontinuation should be documented on the applicable CRF and medical records.

8.STUDY PROCEDURES

8.1 INFORMED CONSENT PROCEDURES

Written informed consent will be obtained from each patient by the Principal Investigator or designee. Informed consent will be prepared according to NRES and study sponsor requirements for informed consents.

Patients who are candidates for the study will receive a Patient Information Sheet (PIS) which explains the purpose of the trial and highlights the benefits and risks of participation in the trial. Patients must be given adequate time (minimum 24 hours) to review the information and must have the opportunity to ask the Principal Investigator or designee any questions relating to the trial. Following this, the patient must sign an informed Consent Form (CF) in the presence of the Principal Investigator or designee who must then countersign the CF.

Written consent must be obtained prior to any study-specific procedures being performed, including any study specific screening procedures prior to randomisation. At the time of consent, participants must be informed that they have the right to withdraw their participation in the trial at any stage and that doing so will not prejudice their future clinical management and care.

8.2.SCREENING (VISIT 1)

Visit schedule screening will entail evaluation of:

- Inclusion and exclusion criteria
- Demographic data including age, gender and medical history
- Characteristics of PR
    - o Number of joint attacks (per month)
    - o Intensity of attack (analogue scale 0-10 cm)
    - o Presence of inflammation during atacks (swelling, eritema)
- Clinical examination
- Concomitant drugs
- Joint asssement
- Routine blood test (include at least CRP, ESR Hb, WBC, platelets, cholesterol, ALT/AST, creatinine)
- Rheumatoid Factor
- Anti-CCP.

Hand and feet radiographs (: unless an X-ray was performed < 6 months)

-

8.3 BASELINE VISIT (VISIT NUMBER 2) AND FOLLOW-UP

- Clinical examination

- Concomitant drugs

Joint asssement

Randomisation

ACR/EULAR criteria for RA

Specific blood tests (include determination of serum ACPA ans anti-CarP

Autoantibodies

Adverse events

8.4 VISIT NUMBERS (VISIT 3)

- Clinical examination
- Concomitant drugs

Joint asssement

ACR/EULAR criteria for RA

Specific blood tests (include determination of serum ACPA ans anti-CarP)

Autoantibodies

Adverse events

8.5 VISITS NUMBERS (VISIT 4, 8, AND EARLY WITHDRAWAL/STOP TREATMENT)

- Clinical examination
- Concomitant drugs

Joint asssement

ACR/EULAR criteria for RA

Routine blood tests

Adverse events

Specific blood tests (include determination of serum ACPA ans anti-CarP)(in Early withdrawal /stop treatment visits)

8.6 VISITS NUMBERS (VISIT5,7, 9)

- Clinical examination
- Concomitant drugs

Joint asssement

ACR/EULAR criteria for RA

Adverse events

8.7 VISITS NUMBERS (VISIT6 AND 10)

- Clinical examination
- Concomitant drugs

Joint asssement

ACR/EULAR criteria for RA

Routine blood test

Specific blood tests

Adverse Events

8.8 STUDY VISIT SCHEDULE

Visit number	1	2	3	4	5	6	7	8	9	10	Treatement cessation/early withdrawal
Timeline (months)		0	3	6	9	12	15	18	21	24	
Informed Consent	x										
Inclusion/ Exclusion criteria	x										
Demographics	x										
Medical history	x										
Concomitant drugs	x	x	x	x	x	x	x	x	x	x	x
Clinical exam	x	x	x	x	x	x	x	x	x	x	x
Joint assessment	x	x	x	x	x	x	x	x	x	x	x
Routine blood test	x			x		x		x		x	x
Hand/feet X-ray*	x										
Randomisation		x									
ACR/EULAR criteria for RA	x	x	x	x	x	x	x	x	x	x	x
Specific blood tests**		x	x			x				x	x
Adverse events		x	x	x	x	x	x	x	x	x	x

RF: rheumatoid factor CCP: cyclic citrullinated peptide.

Routine blood test include at least CRP, ESR Hb, WBC,platelets, cholesterol, ALT/AST creatinine

*: unless an X-ray was performed < 6 months

**: specific blood test include the determination of serum ACPA and anti-CarP autoantibodies

All patients included after the screening will be followed until month 24. Visits will be performed every three months according to standard clinical practice. In each visits the achievement of the RA classification criteria will be analysed. The

frequency, duration and intensity (in a visual analogue scale) of the joint attacks will be analysed together with the presence of joint swelling or erythema. Concomitant therapy will be recorded . Systemic steroids (oral or parenteral) in an equivalent dose higher than 7.5 mg/day of prednisone are not permitted during the study.

8.9 END OF STUDY TRIAL DEFINITION

The end of the study trial will be triggered 6 months after the last recruited patient completed final study visit (Last Patient Last Visit LPLV). This additional months will allow to monitoriting and statistical analysis .

Those patients who achieve the classification criteria of RA at any time during the 24 month of the trial are considered non responders and will discontinued the study protocol and treated according to physician criteria.

Whenever possible, patients will be followed by entering their data in the CRd until the end of 10 visits

9.STATISTICAL ANALYSIS:

General Remarks

The statistical analysis will be carried out in accordance with the principles specified in the International Conference on Harmonization (ICH) Topic E9 (CPMP / ICH / 363/96)[1]. A detailed Statistical Analysis Plan (SAP) agreed upon before the data base closure and the break of the randomisation codes. This SAP will follow the general regulatory recommendations given in the ICHE9[2] guidance, as well as other specific

1 CPMP/ICH/363/96. ICH E9 Statistical Principles for Clinical Trials. URL: http://www.ema.europa.eu/ema/pages/includes/document/open_document.jsp?webContentId=WC500002928, last access: 30-Oct-2017.

2 CPMP/ICH/363/96. ICH E9 Statistical Principles for Clinical Trials. URL: http://www.ema.europa.eu/ema/pages/includes/document/open_document.jsp?webContentId=WC500002928, last access: 30-Oct-2017.

guidance on methodological and statistical issues[3]. Also, it will stick to the recommendations given by the consensus documents of the scientific journals[4,5] to improve reliability and value of medical research literature by promoting transparent and accurate reporting of clinical research studies.

The SAS System[6] (Release 9.4, or an upgraded version), or equivalent validated statistical software, will be the statistical software used to analyse the data sets.

A summary of the overall approach to statistical analysis is presented hereafter.

Sample size calculation

The sample size will be calculated according to the primary endpoint, (progression to RA at 24 months). In the primary analysis, we will report the primary endpoint in each group which will be compared using the Fisher's exact test.

Previous studies found a 2-year progression rate of approximately 40-50%. Assuming a 2-year progression rate of at least 42% in the control group according to previous data and 11% in the experimental group, a sample size of 35 patients per group (total 70) will be needed to reach a power of 80% at a nominal level of a two-sided alpha of 0.05, according to nQuery validated software [7,8,9,10]

The study will be performed in 20 sites (Rheumatology Services or Units) from hospitals) with a median recruitment of 5 patients per centre. The study enrolment period will be 41 months

3 EMEA Scientific Guidelines for Human Medicinal Products, Clinical Efficacy and Safety Guidelines, General Guidelines. URL: http://www.ema.europa.eu/ema/index.jsp?curl=pages/regulation/general/general_content_000602.jsp&mid=WC0b01ac05807d91a4, last access: 30-Oct-2017.

4 Schulz KF, Altman GD, Moher D for the CONSORT Group*. CONSORT 2010 Statement: Updated Guidelines for Reporting Parallel Group Randomized Trials. Ann Intern Med. 2010;152:726-732.

5 EQUATOR-network (Enhancing the Quality and Transparency of Health Research). URL: http://www.equator-network.org/resource-centre/library-of-health-research-reporting/, last access 09-Dec-2016

6 SAS version 9.4 software, SAS Institute Inc., Cary, NC, URL: http://www.sas.com/, last access, 30-Oct-2017.

7 Elashoff JD. nQuery Version 7.0 Advisor User's Guide. Statistical Solutions Ltd, Los Angeles, CA 2007.

8 Fleiss, J.L. Statistical Methods for Rates and Proportions. 2nd Edition John Wiley & Sons Inc., New York (1981) pp. 24-26

9 Chernick, M.R., Liu, C. Y. "The saw-toothed behavior of power versus sample size and software solutions: single binomial proportion using exact methods." The American Statistician 56(2002) pp. 149-155

10 R.G. Thomas and M. Conlon "Sample size determination based on Fisher's exact test for use in 2 x 2 comparative trials with low event rates" Controlled Clinical Trials 13(1992) pp. 134-147

Analysis populations

There will the following analysis populations for this study:

1) Modified Full Analysis Set (mFAS): All patients who are randomized into the study and who have received the investigational product (any of the 2 arms treatment) will be included in the mFAS population. The accepted exclusions, as per the ICHE9, are predefined as follows:
    - Participants who fail to satisfy an entry criterion may be excluded from the analysis without the possibility of introducing bias only under the following circumstances:

i) The entry criterion was measured prior to randomization;

ii) The detection of the relevant eligibility violations can be made completely objectively;

iii) All participants receive equal scrutiny for eligibility violations;

iv) All detected violations of the particular entry criterion are excluded.

2) Per Protocol Population: Per protocol (PP) patient sets will be defined as those patients included in the mFAS set without major protocol deviations that might impact the study's main assessments. These deviations will be assessed during the data review prior to database lock.
3) The Safety population is defined as all randomized participants who received the investigational product (any of the tree arm treatment). In this study the Safety population will have the same definition than the mFAS subset and thus, all safety analysis will be conducted on the mFAS population.

The primary analysis set for the main analyses will follow the '*ICH Topic E9 - Statistical Principles for Clinical Trials' (CPMP/ICH/363/96)*' and will be based on the modified full analysis set (i.e. ITT or mFAS; see beginning of section 0, above). However, the main outcome will be also analysed using the PP set for sensitivity purposes.

The precise reasons for excluding participants from each population will be fully defined and documented independently of the randomization codes during the data blinder review and before data lock.

Subgroup analysis

The subgroups will be prospectively identified in the SAP. In case of any post-hoc subgroup analysis, they will be justified and identified as data-driven and, they will follow the principles and regulatory recommendations[11].

Adjusted analysis

The **main planned analysis** will be unadjusted and any deviation from this plan will be traced and identified in the SAP and/or the final report.

Statistical tests

The Fisher's exact test used for categorical variables, the t-test or the one-way ANOVA for Gaussian distributed variables (for 2 or more than two groups, respectively) and for non-Gaussian continuous variables, non-parametric methods (Mann- Whitney test for two groups or Kruskal-Wallis for more than two groups). A detailed description of tables and tests for each variable will be documented upfront in the SAP.

Interim Analysis and multiplicity adjustments

11 EMA/CHMP/EWP/117211/10. Guideline on the investigation of subgroups in confirmatory clinical trials (Draft). URL: http://www.ema.europa.eu/ema/index.jsp?curl=pages/regulation/general/general_content_001215.jsp&mid=WC0b01ac05807d91a4, last access: 30-Oct-2017.

The purpose of this interim analysis is not to stop the study due to efficacy, which is assessed at 24-month follow-up. It is a descriptive evaluation of the early response, at 12 months of follow-up. There is only one primary outcome in this trial.Therefore, no multiplicity adjustment will be implemented to perform this analysis and p-values will be considered descriptive at their nominal value.

The analysis will follow the principles specified in the ICHE9[12] and the CPMP/EWP/908/99[13] Points to Consider on Multiplicity issues in Clinical Trials guidelines. .

Procedures to account for missing or spurious data

The handling of missing data will follow the principles specified in the ICH-E9[32] and the CPMP/EWP/1776/99 Rev1. Guideline on Missing Data in confirmatory trials Guidelines[14]. Patients with missing data for the primary outcome will be considered as failures irrespectively to the drop-out reason. A detailed more detailed description will be included in the SAP.

DATA HANDLING

An Electronic Case Report Form (eCRF), designed to record all of the required information from the protocol to be reported to the sponsor on each trial participant, will be implemented in this trial. The eCRF Design task includes adapting technology and processes that manage clinical data to produce a high quality, clean and analyzable database. A correct design of an eCRF will impact in the process of collecting, entering,

12 CPMP/ICH/363/96. ICH E9 Statistical Principles for Clinical Trials. URL: http://www.ema.europa.eu/ema/index.jsp?curl=pages/regulation/general/general_content_001228.jsp&mid=WC0b01ac05807d91a4, last access: 30-Oct-2017.

13 CPMP/EWP/908/99. Points to Consider on Multiplicity issues in Clinical Trials. URL: http://www.ema.europa.eu/ema/index.jsp?curl=pages/regulation/general/general_content_001220.jsp&mid=WC0b01ac05807d91a4, last access: 30-Oct-2017.

14 CPMP/EWP/1776/99 Rev1. Guideline on Missing Data in confirmatory trials. URL: http://www.ema.europa.eu/ema/index.jsp?curl=pages/regulation/general/general_content_001221.jsp&mid=WC0b01ac05807d91a4, last access: 30-Oct-2017.

cleaning, and reporting on data recorded during clinical trials. The eCRF design includes:

- Input into the design of protocols, which define what data are to be collected and at what times.
- Design and approval of case report forms, on which participants' data are collected.
- Database design for the study, ensuring it meets requirements for data entry and reporting.

Data generated and/or collected will be registered in participating centres using an electronic data management system with remote data entry. Completeness and plausibility checks will ensure the collection of high quality data. Data management services, in addition to the necessary hard‐ and software, will be provided by the Data Management and Biostatistics Program of the Spanish Clinical Research Network (SCReN). An electronic Case Report Form (eCRF) will be designed, validated and implemented with MACRO™ (Elsevier B.V.) and will provide electronic data capture functionality (EDC) to the investigators. The system complies with the relevant international standards and provides the capability to perform all major data management activities within a consistent, auditable and integrated electronic environment (query management, data entry, data validation, report generation). MACRO™ has been designed to support compliance with the requirements of relevant Competent Authorities of Europe and the rest of the world, including the internationally recognized ICH Good Clinical Practice and FDA 21 CFR Part 11. Any data transfer will be done using secure SSL connection with encryption. Export for archiving of the clinical database including audit trails in hard- and software independent storage

formats will be provided by the Medical Statistics core facility of IDIBAPS-Hospital Clinic.

Additional services will include submission protocols, user manuals and online control for configuration and incidences.

Palindromic Rheumatism criteria of Guerne & Weisman (1992) (18)

ƒ. Six-month history of brief-sudden-onset and recurrent episodes of monoarthritis or, rarely, polyarthritis or of soft tissue inflammation

ƒ. Direct observation of one attack by a physician

ƒ. Three or more joints involved in different attacks

ƒ. Absence of erosions on radiographs

ƒ. Exclusion of other arthritides

We use modified Palindromic Rheumatism criteria of Guerne and Weissman for this study:

- Disease evolution less than 6 month is permitted (but not less than 3 months).
- All patients included should be seropositive for RF and/or ACPA.

10. ADVERSE EVENT REPORTING

A **S*erious Adverse Event (SAE)*** is any untoward medical occurrence that at any dose:

- results in death
- is life-threatening (defined as an event in which the participant was at risk of death at the time of the event; it does not refer to an event which hypothetically might have caused death if it were more severe)
- requires inpatient hospitalization or causes prolongation of existing hospitalization (see **NOTE** below)
- results in persistent or significant disability/incapacity
- is a congenital anomaly/birth defect
- is an important medical event (defined as a medical event(s) that may not be immediately life-threatening or result in death or hospitalization but, based upon appropriate medical and scientific judgment, may jeopardize the subject or may require intervention [eg, medical, surgical] to prevent one of the other serious outcomes listed in the definition above.) Examples of such events include, but are not limited to, intensive treatment in an emergency room or at home for allergic bronchospasm; blood dyscrasias or convulsions that do not result in hospitalization.)
- Suspected transmission of an infectious agent (eg, pathogenic or nonpathogenic) via the study drug is an SAE.

Although pregnancy, overdose, potential drug-induced liver injury (DILI), and cancer are not always serious by regulatory definition, these events must be handled as SAEs. Any component of a study endpoint that is considered related to study therapy should be reported as an SAE (eg, death is an endpoint, if death occurred due to anaphylaxis, anaphylaxis must be reported).

NOTE: (PI determines if this information regarding hospitalizations are considered SAEs and should be included in the protocol. This is supplemental information that is included in BMS-sponsored trials)

The following hospitalizations are not considered SAEs in BMS clinical studies:

- a visit to the emergency room or other hospital department < 24 hours, that does not result in admission (unless considered an important medical or life-threatening event)
- elective surgery, planned prior to signing consent
- admissions as per protocol for a planned medical/surgical procedure
- routine health assessment requiring admission for baseline/trending of health status (eg, routine colonoscopy)
- Medical/surgical admission other than to remedy ill health and planned prior to entry into the study. Appropriate documentation is required in these cases.
- Admission encountered for another life circumstance that carries no bearing on health status and requires no medical/surgical intervention (eg, lack of housing, economic inadequacy, caregiver respite, family circumstances, administrative reason).
- Admission for administration of anticancer therapy in the absence of any other SAEs (applies to oncology protocols)

The researcher must send the completed SAEs of the study immediately and within 24 hours of the site becoming aware of the event. by mail in the name of the study monitor or by fax to 932279877.

ADVERSE EVENTS

An Adverse Event (AE) is defined as any new untoward medical occurrence or worsening of a preexisting medical condition in a clinical investigation participant administered study drug and that does not necessarily have a causal relationship with this treatment. An AE can therefore be any unfavorable and unintended sign (such as an abnormal laboratory finding), symptom, or disease temporally associated with the use of investigational product, whether or not considered related to the investigational product.

The causal relationship to study drug is determined by a physician and should be used to assess all adverse events (AE). The casual relationship can be one of the following:

Related: There is a reasonable causal relationship between study drug administration and the AE.

Not related: There is not a reasonable causal relationship between study drug administration and the AE.

The term "reasonable causal relationship" means there is evidence to suggest a causal relationship.

Adverse events can be spontaneously reported or elicited during open-ended questioning, examination, or evaluation of a subject. (In order to prevent reporting bias, subjects should not be questioned regarding the specific occurrence of one or more AEs.)

NONSERIOUS ADVERSE EVENT

- Non-serious Adverse Events (AE) are to be provided to BMS in aggregate via interim or final study reports as specified in the agreement or, if a regulatory requirement [eg, IND US trial] as part of an annual reporting requirement.
- Non-serious AE information should also be collected from the start of a placebo lead-in period or other observational period intended to establish a baseline status for the subjects.

A ***non-serious adverse event*** is an AE not classified as serious.

Non-serious Adverse Event Collection and Reporting

The collection of non-serious AE information should begin at initiation of study drug. All non-serious adverse events (not only those deemed to be treatment-related) should be collected continuously during the treatment period and for a minimum of 30 days following the last dose of study treatment.

Non-serious AEs should be followed to resolution or stabilization, or reported as SAEs if they become serious. Follow-up is also required for non-serious AEs that cause interruption or discontinuation of study drug and for those present at the end of study treatment as appropriate.

Laboratory Test Abnormalities

All laboratory test results captured as part of the study should be recorded following institutional procedures. Test results that constitute SAEs should be documented and reported to BMS as such.The following laboratory abnormalities should be documented and reported appropriately: any laboratory test result that is clinically significant or meets the definition of an SAE any laboratory abnormality that required the participant to have study drug discontinued or interrupted any laboratory abnormality that required the subject to receive specific corrective therapy.

It is expected that wherever possible, the clinical rather than laboratory term would be used by the reporting investigator (eg, anemia versus low hemoglobin value).

Potential Drug Induced Liver Injury (DILI)

Wherever possible, timely confirmation of initial liver-related laboratory abnormalities should occur prior to the reporting of a potential DILI event. All occurrences of potential DILIs, meeting the defined criteria, must be reported as SAEs.

Potential drug induced liver injury is defined as:

1) AT (ALT or AST) elevation > 3 times upper limit of normal (ULN)

AND

2) Total bilirubin > 2 times ULN, without initial findings of cholestasis (elevated serum alkaline phosphatase)

AND

3) No other immediately apparent possible causes of AT elevation and hyperbilirubinemia, including, but not limited to, viral hepatitis, pre-existing chronic or acute liver disease, or the administration of other drug(s) known to be hepatotoxic.

Pregnancy

If, following initiation of the investigational product, it is subsequently discovered that a study participant is pregnant or may have been pregnant at the time of investigational product exposure, including during at least 5 half-lives after product administration, the investigational product will be permanently discontinued in an appropriate manner (eg, dose tapering if necessary for participant).

The investigator must immediately notify Worldwide.Safety@bms.com of this event via the Pregnancy Surveillance Form in accordance with SAE reporting procedures.

Follow-up information regarding the course of the pregnancy, including perinatal and neonatal outcome and, where applicable, offspring information must be reported on the Pregnancy Surveillance Form [provided upon request from BMS]

Any pregnancy that occurs in a female partner of a male study participant should be reported to BMS. Information on this pregnancy will be collected on the Pregnancy Surveillance Form. In order for Sponsor or designee to collect any pregnancy surveillance information from the female partner, the female partner must sign an informed consent form for disclosure of this information.

Overdose

An overdose is defined as the accidental or intentional administration of any dose of a product that is considered both excessive and medically important. All occurrences of overdose must be reported as an SAE.

Other Safety Considerations

Any significant worsening noted during interim or final physical examinations, electrocardiograms, X-rays, and any other potential safety assessments, whether or not these procedures are required by the protocol, should also be recorded as a non-serious or serious AE, as appropriate, and reported accordingly.

Serious Adverse Event Collecting and Reporting

- All Serious Adverse Events (SAEs) that occur following the subject's written consent to participate in the study through 30* days of discontinuation of dosing must be reported to BMS Worldwide Safety, whether related or not related to study drug. If applicable, SAEs must be collected that relate to any later protocol-specified procedure (eg, a follow-up skin biopsy).
- Following the subject's written consent to participate in the study, all SAEs, whether related or not related to study drug, are collected, including those thought to be associated with protocol-specified procedures. The investigator should report any SAE occurring after these aforementioned time periods, which is believed to be related to study drug or protocol-specified procedure.
- An SAE report should be completed for any event where doubt exists regarding its seriousness;
- If the investigator believes that an SAE is not related to study drug, but is potentially related to the conditions of the study (such as withdrawal of previous

therapy or a complication of a study procedure), the relationship should be specified in the narrative section of the SAE Report Form.

- If the BMS safety address is not included in the protocol document (eg, multicenter studies where events are reported centrally), the procedure for safety reporting must be reviewed/approved by the BMS Protocol Manager. Procedures for such reporting must be reviewed and approved by BMS prior to study activation.
- The BMS SAE form should be used to report SAEs. If the BMS form cannot be used, another acceptable form (ie, CIOMS or Medwatch) must be reviewed and approved by BMS. The BMS protocol ID number must be included on whatever form is submitted by the Sponsor/Investigator. The CIOMS form is available at: http://www.cioms.ch/index.php/cioms-form-i.
- Worldwide.Safety@bmsaepbusinessprocess@bms.com
- In accordance with local regulations, BMS will notify investigators of all reported SAEs that are suspected (related to the investigational product) and unexpected (ie, not previously described in the IB). An event meeting these criteria is termed a Suspected, Unexpected Serious Adverse Reaction (SUSAR). Investigator notification of these events will be in the form of a SUSAR Report.
    - Other important findings which may be reported by BMS as an Expedited Safety Report (ESR) include: increased frequency of a clinically significant expected SAE, an SAE considered associated with study procedures that could modify the conduct of the study, lack of efficacy that poses significant hazard to study subjects, clinically significant safety finding from a nonclinical (eg, animal) study, important safety recommendations from a study data monitoring

committee, or sponsor decision to end or temporarily halt a clinical study for safety reasons.

- Upon receiving an ESR from BMS, the investigator must review and retain the ESR with the IB. Where required by local regulations or when there is a central IRB/IEC for the study, the sponsor will submit the ESR to the appropriate IRB/IEC. The investigator and IRB/IEC will determine if the informed consent requires revision. The investigator should also comply with the IRB/IEC procedures for reporting any other safety information.
- In addition to the Sponsor Investigator's responsibility to report events to their local HA, suspected serious adverse reactions (whether expected or unexpected) shall be reported by BMS to the relevant competent health authorities in all concerned countries according to local regulations (either as expedited and/or in aggregate reports).

SAEs, whether related or not related to study drug, and pregnancies must be reported to BMS within 24 hours. SAEs must be recorded on BMS or an approved form; pregnancies must be reported on a Pregnancy Surveillance Form.

SAE Email Address: ebonfill@clinic.cat cc martelli@clinic.cat cc acruceta@clinic.cat and also Worldwide.Safety@BMS.com

SAE Facsimile Number: + 93 2279877 and also +1 609-818-3804

If only limited information is initially available, follow-up reports are required. (Note: Follow-up SAE reports should include the same investigator term(s) initially reported.) If an ongoing SAE changes in its intensity or relationship to study drug or if new information becomes available, a follow-up SAE report should be sent within 24 hours

to BMS (or designee) using the same procedure used for transmitting the initial SAE report.

All SAEs should be followed to resolution or stabilization.

SAEs should be reported on MedWatch Form 3500A, which can be accessed at:

http://www.accessdata.fda.gov/scripts/medwatch/

11. DATA HANDLING AND RECORD KEEPING

11.1 CONFIDENTIALITY

This study complies with the section on Protection of personal data in the information sheet to the subject (HIP / CI) regarding the Regulation (EU) No. 2016/679 General Data Protection , in force in Spain since May 25, 2018

11.2 CASE REPORD FORM

Data collection will be in the form of completing electronic CRFs via the trial database to record all the required assessments at each study visit.

11.3 COMPLIANCE

This trial will be conducted in accordance with the principles of Good Clinical Practice (GCP) as laid out in the EU directive and The Medicines for Human Use (Clinical Trials) Regulation 2004, and its amendments.

The study will be sent and approved by the Committee of Ethics in Research (CEIm / REC) as well as by the Spanish Agency on Medicines and Medical devices (AEMPS) in accordance with the current Legislation. Royal Decree 1090/2015 of December 4 and European Regulation 536/2014 of April 16.

Changes in the protocol that may increase risk exposure or present risks to the patient, or may adversely affect the validity of the study, must be approved in writing by the sponsor and then by the REC and AEMPS before implementing the change. These changes are usually presented in the form of an amendment.

In addition, internal auditors and Competent Authority inspectors will be allowed access to CRFs, source documents and other trial files to evaluate the trial. Audit reports will be kept confidential.

11.4 SERIOUS BREACHES IN GPC OR THE TRIAL PROTOCOL

All investigators participating in the trial will promptly notify the Chief Investigator or Sponsor of a serious breach, as soon as they become aware of. The CI is then responsible for notifying the CRO within 24 hours of becoming aware of a serious breach.

The Sponsor is responsible for notifying the licensing authority in writing of any serious breach of:

(a) The conditions and principles of GCP in connection with that trial; or

(b) The protocol relating to that trial, within 7 days of becoming aware of that breach.

A "serious breach" is a breach which is likely to effect to a significant degree:-

The safety or physical or mental integrity of the subjects of the trial; or the scientific value of the trial.

Participating centers should contact the CTU -hospital Clinic office or monitors for further information.

11.5 PUBLICATION POLICY

The sponsor is the final responsible for the publication.

Authorship of the final manuscript (s), interim publications, or abstracts will be decided according to active participation in the study design, trial management and accrual group of patients. Contributing centers (and participating Investigators) will be acknowledged in the final manuscript. Representatives for the Sponsor will be added, as appropriate, as co-authors. No participant may present data from his / her center separately from the rest of the trial results unless approved by the Sponsor management group.

11.6 FUNDING

The sponsor has received a grant from BMS for the development of the study.

12. REFERENCES

1. Sanmarti R, Canete JD, Salvador G. Palindromic rheumatism and other relapsing arthritis. Best Pract Res Clin Rheumatol 2004;18:647-61.

2. Corominas H, Narváez J, Díaz-Torné C, Salvador G, Gomez-Caballero ME, de la Fuente D et al. Diagnostic and therapeutic delay of rheumatoid arthritis and its relationship with health care devices in Catalonia. The AUDIT study. Reumatol Clin. 2016 May-Jun;12(3):146-50.

3. Koskinen E, Hannonen P, Sokka T. Palindromic rheumatism: longterm outcomes of 60 patients diagnosed in 1967-84. J Rheumatol. 2009 Sep;36(9):1873-5.

4. Gonzalez-Lopez L, Gamez-Nava JI, Jhangri GS, Ramos-Remus C, Russell AS, Suarez-Almazor ME. Prognostic factors for the development of rheumatoid arthritis and other connective tissue diseases in patients with palindromic rheumatism. J Rheumatol 1999;26:540-5.

5. Russell AS, Devani A, Maksymowych WP. The role of anti-cyclic citrullinated peptide antibodies in predicting progression of palindromic rheumatism to rheumatoid arthritis. J Rheumatol 2006;33:1240-2.

6. Salvador G, Gomez A, Vinas O, Ercilla G, Canete JD, Munoz-Gomez J, et al. Prevalence and clinical significance of anti-cyclic citrullinated peptide and antikeratin antibodies in palindromic rheumatism. An abortive form of rheumatoid arthritis? Rheumatology (Oxford) 2003;42:972-5.

7. Tamai M, Kawakami A, Iwamoto N, Arima K, Aoyagi K, Eguchi K. Contribution of anti-CCP antibodies, proximal interphalangeal joint involvement, HLA-DRB1 shared epitope, and PADI4 as risk factors for the development of rheumatoid arthritis in palindromic rheumatism. Scand J Rheumatol 2010;39:287-91.

8. Khabbazi A, Hajialiloo M, Kolahi S, Soroosh M, Esalatmanesh K, Sharif S. A multicenter study of clinical and laboratory findings of palindromic rheumatism in Iran. Int J Rheum Dis 2012;15:427-30.

9. Sanmarti R, Cabrera-Villalba S, Gomez-Puerta JA, Ruiz-Esquide V, Hernandez MV, Salvador G, et al. Palindromic rheumatism with positive anticitrullinated peptide/protein antibodies is not synonymous with rheumatoid arthritis. A longterm followup study. J Rheumatol 2012;39:1929-33.

10. Cabrera-Villalba S, Gomara MJ, Cañete JD, Ramírez J, Salvador G, Ruiz-Esquide V, Hernández MV, Inciarte-Mundo J, Haro I, Sanmartí R. Differing specificities and isotypes of anti-citrullinated peptide/protein antibodies in palindromic rheumatism and rheumatoid arthritis. Arthritis Res Ther. 2017 Jun 15;19(1):141.

11. Ioan-Facsinay A, Willemze A, Robinson DB, Peschken CA, Markland J, van der Woude D, et al. Marked differences in fine specificity and isotype usage of the anti-citrullinated protein antibody in health and disease. Arthritis Rheum 2008;58:3000-8.

12. Barra L, Scinocca M, Saunders S, Bhayana R, Rohekar S, Racape M, et al. Anti-citrullinated protein antibodies in unaffected first-degree relatives of rheumatoid arthritis patients. Arthritis Rheum 2013;65:1439-47.

13. Youssef W, Yan A, Russell AS. Palindromic rheumatism: a response to chloroquine. J Rheumatol. 1991 ;18:35-7.

14. Gonzalez-Lopez L, Gamez-Nava JI, Jhangri G, Russell AS, Suarez-Almazor ME. Decreased progression to rheumatoid arthritis or other connective tissue diseases in patients with palindromic rheumatism treated with antimalarials. J Rheumatol 2000;27:41-6.

15. Keating GM. Abatacept: a review of its use in the management of rheumatoid arthritis. Drugs. 2013 l;73:1095-119.

16. Gottenberg JE, Ravaud P, Cantagrel A, Combe B, Flipo RM, Schaeverbeke T et al. Positivity for anti-cyclic citrullinated peptide is associated with a better response to abatacept: data from the 'Orencia and Rheumatoid Arthritis' registry. Ann Rheum Dis. 2012;71:1815-9.
17.Huizinga TWJ, Connolly SE, Johnsen A, Zhu J, Furst DE, BykerkVP. et al. effect of anti-cyclic citrullinated peptide 2 immunoglobulin M serostatus on efficacy outcomes following treatment with abatacept plus methotrexate in the AVERT trial. Ann RheumDis 2015;74(Suppl2): 234.
18. Guerne PA, Weisman MH. Palindromic rheumatism: part of or apart from the spectrum of rheumatoid arthritis. Am J Med. 1992;93:451-60.
