# Supplementary material for: Abatacept versus hydroxychloroquine for prevention of rheumatoid arthritis in individuals with palindromic rheumatism: a randomized open-label trial
Source: Nat Med. 2026 May 14;32(7):2610–8. doi: 10.1038/s41591-026-04395-6 (PMC13375639; doi:10.1038/s41591-026-04395-6)
Supplement: Supplementary file 3 — Peer Review File [file 41591_2026_4395_MOESM3_ESM.pdf]

nature portfolio

Peer Review File

ABATACEPT FOR PREVENTION OF RHEUMATOID ARTHRITIS IN INDIVIDUALS WITH PALINDROMIC RHEUMATISM: A RANDOMIZED TRIAL

Corresponding Author: Dr Raimon Sanmarti

This file contains all reviewer reports in order by version, followed by all author rebuttals in order by version.

Version 0:

Reviewer comments:

Reviewer #1

(Remarks to the Author)
This paper reports a randomised trial comparing abatacept with hydroxychloroquine for preventing individuals developing rheumatoid arthritis. The results demonstrate abatacept is significantly better in terms of proportion developing RA and time until RA occurs. I had some minor comments on the paper below, predominantly from a statistical point of view.

1. Abstract: For the primary endpoint results, I would recommend that the estimated difference between the two arms is given together with a 95% confidence interval.

2. Abstract: the clinicaltrials.gov registration page mentions a secondary endpoint was serum APCA. As a non-expert, it wasn't clear to me if this was one of the endpoints in the abstract (joint attacks or symptom remission) but if not I would recommend it is reported.

3. Introduction: I would have welcomed more information about why HCQ was used as the comparator group in this trial. Is it currently used to treat PR?

4. Figure 2: I would recommend that the n in both analyses is either included in the plot legend or just in the caption, to avoid confusion.

5. Page 8: "The results in the per-protocol population showed a trend similar to that obtained in the mFAS population" – I would rewrite this as saying that the results were also statistically significant in the per-protocol population.

6. Methods, randomisation: can more information be given about the randomisation technique? Was it complete randomisation, or stratified, or something else?

7. Methods, outcomes: as per comment on the abstract, the secondary endpoints do not appear to correspond to the ones on the clinicaltrials.gov page. Could this be clarified?

Reviewer #2

(Remarks to the Author)
A: The results are clinically relevant for patients and health professionals.
B: The results are novel and as mentioned clinically relevant, also for the society in regards of health oeconomics, since the cost of rheumatoid arthritis is rather high.
C: The study is presented in agreement with the EQUATOR group's CONSORT guidelines for randomized trials. Power calculations is obviously difficult to perform since this intervention has not been evaluated before, but appears to be sufficient in this case. The comparotor "plaquenil" is relevant since it is the most frequent and currently best validated treatment option for this condition.

D: Yes, and performed as presented in the pre-specified sigend statistical analysis plan, that also follw the CONSORT guideline proposed by the EQUATOR group.
E: The conclusions are hunble and relvant and in agreement with the presented results.
F: No suggestions for improvement.
G: No further references.
H: Clear abstract introduction and also visually well presented results, no further suggestions.

Reviewer #3

(Remarks to the Author)
A. Summary of key results
This randomized, open-label, multicenter phase IV trial (PALABA, NCT03669367, EudraCT 2017-004543-20) evaluated whether two years of abatacept (SC, 125 mg weekly then biweekly) could prevent progression from seropositive palindromic rheumatism (PR) to rheumatoid arthritis (RA), compared with oral hydroxychloroquine (HCQ, 5 mg/kg/day).
Among 70 patients (34 abatacept, 36 HCQ) included in the modified full analysis set (mFAS), 20.6% of abatacept-treated and 50.0% of HCQ-treated patients developed RA at 24 months (p=0.010; HR 0.27, 95% CI 0.07–0.96). Abatacept was also associated with higher remission (55.9% vs. 22.9%, p=0.007) and lower attack intensity. Both drugs were well tolerated; no unexpected safety issues emerged.

B.Originality and significance
The trial addresses a clinically relevant and underexplored population: individuals with seropositive PR, a recognized pre-RA phenotype. Prior prevention trials (PRAIRI, APIPPRA) demonstrated that biologics (rituximab, abatacept) could delay or prevent RA onset in ACPA-positive arthralgia without clinical arthritis. Extending this concept to PR is innovative.
However, the originality is partial, as abatacept's preventive effect in at-risk populations was already shown (e.g., Emery et al., Ann Rheum Dis 2022; Gerlag et al., Lancet 2019). The key novelty lies in targeting the PR subset, yet the sample size and open-label design substantially limit external validity.

C. Data & methodology
The study is a phase IV, multicenter, open-label, randomized controlled trial with 1:1 allocation, stratified by site (PROC PLAN in SAS, block randomization). Inclusion criteria (PR by Guerne & Weissman modified criteria, ACPA+ and/or RF+) are appropriate. Exclusion of seronegative PR was justified mechanistically.
Major methodological weaknesses:
-Open-label design without blinded assessment of the primary endpoint (transition to RA) introduces detection and expectation bias.
- Sample size (n=70) was below the prespecified target (35 per arm achieved, not exceeded), leaving limited power for secondary analyses or adjustment for confounders.
- Outcome ascertainment relied on treating clinicians' judgment using ACR/EULAR 2010 criteria, which is valid but prone to variability. No central adjudication was reported.
- Heterogeneity in baseline ACPA positivity (97% in HCQ vs. 82% in abatacept) might confound results, particularly as ACPA status strongly predicts RA progression.
- Duration of attacks ≥72h was more frequent in HCQ arm (30.8% vs. 11.5%), suggesting possible baseline imbalance toward more active disease in the control group.
- The study lacked imaging (e.g., US/MRI) assessment of subclinical synovitis, a key predictor of progression.
The CONSORT checklist and protocol confirm correct ethical conduct, prospective registration, and pre-specified outcomes, but methodological rigor is weakened by open-label design, small sample, and lack of central outcome validation.

D. Appropriate use of statistics and treatment of uncertainties
The Statistical Analysis Plan (SAP, 25 June 2024) follows ICH E9 and ICHE91 recommendations, using SAS 9.4. Analyses included: mFAS and per-protocol (PP) populations; failure imputation and "available data only" (ADO) approaches; chi-square or Fisher's exact test for binary endpoints; Kaplan-Meier/log-rank for time-to-RA progression.
While the statistical framework is technically sound, several issues arise:
- Multiplicity and interim analyses: No correction for multiple comparisons or exploratory analyses (p-values nominal).
- Confidence intervals are sometimes missing or reported only for hazard ratios.
- No modeling of baseline imbalances (ACPA levels, attack duration).
- No predefined subgroup analyses despite clear biological heterogeneity.
- No analysis of ACPA titres—although listed in SAP and protocol—was presented in the manuscript, suggesting a deviation from the pre-specified plan.
- Absence of power reassessment or interim futility check, although sample was slightly under target.
Thus, the statistical analysis is adequate but minimal, not fully exploiting available data nor addressing key uncertainties.

E. Conclusions: robustness, validity, reliability
The conclusion that abatacept reduces progression from PR to RA is directionally plausible and supported by numerical differences;
However:
- The study's open-label nature and small sample compromise robustness.
- The absolute event count (7 vs. 18 progressors) is low, with wide CIs.
- Potential selection and ascertainment bias (clinician-assessed RA diagnosis) likely inflate apparent effect size.
- Lack of biomarker correlation (ACPA titres, T-cell or B-cell modulation) weakens mechanistic inference.
Thus, while findings are hypothesis-generating, they do not provide definitive evidence of abatacept's preventive efficacy in

PR.

F. Suggested improvements
For potential future trials:
- Blinded adjudication of primary endpoint (RA diagnosis by independent panel).
- Larger, placebo-controlled design or double-dummy comparison with blinded evaluation.
- Mandatory baseline imaging (US or MRI) to stratify subclinical synovitis.
- Quantitative ACPA titres longitudinal analysis, as per SAP.
- Standardization of attack diaries and validated patient-reported outcomes.
- Longer post-treatment follow-up to assess durability of preventive effect.
- Centralized statistical analysis with covariate adjustment (e.g., mixed logistic or Cox models).

G. References and credit to previous work
The authors cited major relevant trials (PRAIRI, APIPPRA) and background studies on PR and ACPA, but key mechanistic or biomarker references (e.g., T-cell exhaustion under abatacept, preclinical autoimmunity networks) could be expanded.

H. Clarity and context
- The abstract and introduction correctly described rationale and results.
However:
- The abstract overstates the robustness of conclusions given design limitations.
- The methods section lacks detail on randomization concealment and handling of missing data.
- Tables and supplementary materials are clear, but figure legends should specify the analysis population and statistical test used.
- The discussion could better contextualize findings within the broader "RA prevention" field, acknowledging the exploratory nature.

Reviewer #4

(Remarks to the Author)
This is an interesting and well-written paper. It is a randomized clinical trial comparing abatacept with hydroxychloroquine for the prevention of progression to rheumatoid arthritis in patients with palindromic rheumatism.

Although a randomized trial, the sample size is relatively small. However, PR is a rare disease and has been poorly studied with no previous randomized trials. As such this is an important study. However there are a number of major limitations:

1. Unlike recent RA prevention trials, abatacept has been administered for two years rather than one. There is no observational period after therapy. Instead, in the second year the dose is reduced to alternate weekly - there is no explanation for this rationale. Why did the authors decide to do this? If abatacept were to be used in clinical practice, what regimen would be proposed?

2. The groups are not balanced for ACPA. This is potentially important as there us a higher proportion of ACPA+ patients in the HCQ arm. Given ACPA is a major risk factor for progression to RA, how did the authors adjust for this?

3. A major issue with this trial is the recording of symptoms (i.e. flares). Patients were seen periodically only. How were flares recorded? Were patients given diaries to fill in or was it recorded on an app? Recounting flares is notoriously difficult when done retrospectively and patients can forget the number of flares they have had. Similarly how was intensity measured? Was this an average intensity? Flares can vary in intensity and if patients have multiple flares between visits how did they report intensity which may have been variable?

4. What was flare intensity designed to measure? Flares are multi-faceted and can include pain, swelling, redness etc. How are each of these features recorded? e.g. if a flare was associated with lots of swelling but minimal pain, would that be recorded differently to a flare with pain but no swelling? This is an important area to consider but there is minimal information provided.

5. Importantly there was no difference in median number of flares between the two arms. This could be interpreted as no difference in the effect of abatacept vs hydroxychloroquine on the treatment of PR as patients are often most concerned about how often their flares occur.

6. Another major concern is permitted use of low dose steroid, colchicine and NSAID, all of which can reduce flare intensity and potentially even the frequency if used regularly. How did use compare between the arms?

7. What was median time to progression to RA between the two groups?

8. primary outcome was progression to RA. If patients had swelling at one of the study visits, were they recalled to be reassessed? How was this done? What if there was persistent synovitis but the patient did not meet ACR EULAR criteria for RA?

9. Table 1 should include p values to evaluate between-group diffrences. Number of joints involved in a flare should be included. Concomitant steroid, colchicine and NSAID use should be compared formally.

10. How do the authors interpret cholesterol and lymphocye count changes in the ABT arm?

Version 1:

Reviewer comments:

Reviewer #1

(Remarks to the Author)
Thank you to the authors for addressing my previous comments well. I have no further issues to raise.

Reviewer #2

(Remarks to the Author)
No further comments.

Reviewer #3

(Remarks to the Author)

The revised manuscript reports a randomized, open-label phase IV trial showing reduced 24-month progression to RA with abatacept versus hydroxychloroquine (20.6% vs 50.0%; risk difference 29.4%, 95% CI 8.2–50.7; HR 0.27). Sensitivity analyses adjusting for baseline imbalances are now included and remain consistent with the primary findings.

- The study remains clinically relevant as the first randomized trial conducted specifically in palindromic rheumatism (PR). Although RA-prevention strategies with abatacept have been tested in at-risk arthralgia populations (APIPPRA, ARIAA), extension to PR is novel and addresses an unmet need.

- The authors have adequately clarified the randomization procedure, acknowledged the lack of blinding and central adjudication, and explicitly discussed sample size limitations. The addition of post-hoc adjusted analyses meaningfully strengthens the robustness of the primary outcome.

- Inclusion of the previously omitted AMPA (autoantibody) analyses improves transparency and alignment with the protocol. The exploratory results show limited modulation and are appropriately interpreted cautiously.

- Statistical reporting has improved: confidence intervals are now provided for primary effect sizes, and the manuscript clearly states that only one confirmatory primary endpoint exists, with all other p-values considered descriptive.

- The absence of multiplicity correction for secondary endpoints is now transparent and methodologically acceptable given the single primary endpoint structure.

- The conclusions are generally balanced and acknowledge key limitations (open-label design, no imaging, no central adjudication, modest sample size). The statement regarding potential clinical use of abatacept could remain cautious but is reasonably framed.

-,Minor suggestion: consider briefly mentioning exploratory autoantibody findings in the abstract for completeness and clarifying in the Discussion that secondary analyses are descriptive.

Overall, the authors have satisfactorily addressed the main methodological and statistical concerns raised in the initial review. The revised manuscript is substantially improved.

Reviewer #4

(Remarks to the Author)
Some of the points have been addressed. However there are still major concerns, particularly related to two areas:

1/ The authors acknowledge that participants were not offered flare diaries or apps to record their flares between visits. In my view this is a major limitation as it brings into question the validity of assessment of symptom improvement in the trial. They say that participants were instructed that various factors about their flares (frequency, duration, location ,intensity etc etc) would be recorded at study visits but there was no assistance or guidance given to patients or investigators about how to do this in a standardized way. This is very likely to have led to inconsistent data capture across the multiple centers involved. They mention a low median frequency of flares in each arm, but the range is quite wide and some patients were having one or two attacks per month and so would have potentially been affected.

2/ The authors acknowledge that flare 'intensity' was not defined and is subject to interpretation. As such it is likely to have led to inconsistent data capture across the trial. They have added a sentence in the methods that is mis-leading as the investigators were not told to do this in any standardised way.

3/ The question about assessment of the primary outcome has not been answered is a satisfactory way. What if an ACPA+ PR patient presented at the 3 month assessment with three swollen small joints? How would the investigator know if this patient was in a flare or whether they had now developed RA? Either way theuy would classify as ACR-EULAR RA on the day of assessment. The authors need to clarify the instructions provided to investigators to be able to distinguish PR flare from RA development.

Open Access This Peer Review File is licensed under a Creative Commons Attribution 4.0 International License, which permits use, sharing, adaptation, distribution and reproduction in any medium or format, as long as you give appropriate credit to the original author(s) and the source, provide a link to the Creative Commons license, and indicate if changes were made.
In cases where reviewers are anonymous, credit should be given to 'Anonymous Referee' and the source.
The images or other third party material in this Peer Review File are included in the article’s Creative Commons license, unless indicated otherwise in a credit line to the material. If material is not included in the article’s Creative Commons license and your intended use is not permitted by statutory regulation or exceeds the permitted use, you will need to obtain permission directly from the copyright holder.
To view a copy of this license, visit https://creativecommons.org/licenses/by/4.0/

Abatacept for prevention of rheumatoid arthritis in individuals with palindromic rheumatism: a randomized trial " NMED-A145314

Response to referees

Reviewer #1 (Remarks to the Author):

This paper reports a randomised trial comparing abatacept with hydroxychloroquine for preventing individuals developing rheumatoid arthritis. The results demonstrate abatacept is significantly better in terms of proportion developing RA and time until RA occurs. I had some minor comments on the paper below, predominantly from a statistical point of view.

1. Abstract: For the primary endpoint results, I would recommend that the estimated difference between the two arms is given together with a 95% confidence interval.

As suggested, we have added the risk difference and 95% CI for the primary endpoint. Thus, the sentence currently reads as follows:

"In the primary analysis, in the modified full analysis set with failure imputation, 7 (20.6%) of the 34 participants treated with abatacept and 18 (50.0%) of the 36 participants treated with hydroxychloroquine developed RA during the 24 months of follow-up (p=0.010; risk difference 29.4% [95% confidence interval 8.2 to 50.7]), meeting the primary endpoint".

2. Abstract: the clinicaltrials.gov registration page mentions a secondary endpoint was serum APCA. As a non-expert, it wasn't clear to me if this was one of the endpoints in the abstract (joint attacks or symptom remission) but if not I would recommend it is reported.

The reviewer is right. As mentioned in the study procedures, serum biomarkers were tested at baseline and at 3, 12, and 24 months. According to the protocol, effect of treatment on AMPA titers was a secondary objective. This endpoint was intended to serve the purpose of hypothesis generation (i.e., an exploratory endpoint) and was not included in the manuscript. However, following the reviewer's suggestion, we have included detailed information on this endpoint in the Methods and Results sections and a brief comment has been added in the Discussion section. Owing to its exploratory nature, we do not consider it necessary to include any mention in the abstract.

In Methods we have included the following information:

"For evaluating the effect of study treatments on Anti-Modified Peptide/protein Antibodies (AMPAs) we determined by means of appropriate ELISA methods seven antigens (Supplementary Material) with different posttranslational modifications: 1) peptides bearing one posttranslational modification which included citrullinated peptides derived from vimentin (Vim-P55), α-enolase (CEP-1) and fibrin/filaggrin (Chimeric Fibrin Filaggrin Citrullinated Peptide, CFFCP), and homocitrulinated peptide (Chimeric Fibrin Filaggrin Homocitrullinated Peptide [CFFHP]); 2) peptides bearing multiple posttranslational modifications which included Chimeric Fibrin Filaggrin Citrullinated Homocitrullinated Peptide (CFFCHP) and Chimeric Fibrin Filaggrin Citrullinated Homocitrullinated Acetylated Peptide (CFFCHAP); and 3) the protein antigen Carbamylated fetal calf serum (FCS-CarP). We used a peptide with unmodified basal structure, Chimeric Fibrin Filaggrin Peptide (CFFP) as a control peptide. For each

antigen specificity, we determined three isotypes: IgG, IgA, and IgM. The effects of abatacept and hydroxychloroquine on autoantibody levels, specificities, and isotypes were evaluated at baseline and at 3, 12, and 24 months of follow-up for each patient included in the study.

For the analysis of the effect on AMPAs, each antigen-antibody pair was represented as a continuous variable, the Δ optical density. The Δ optical density was calculated by subtracting the optical density of each antigen-antibody pair without posttranslational modifications from the corresponding antigen-antibody pair with posttranslational modifications. Δ optical density measurements for each antigen-antibody pair at 0, 3, 12, and 24 months were analyzed using a parametric approach (see details below) on previously log-transformed data (Geometric Mean [GM]). Between-group and within-group comparisons were evaluated using the GM ratio and Geometric Mean Fold Rise (GMFR). The parametric approach consisted of a restricted maximum likelihood (REML)-based repeated measures approach (Mixed Models for Repeated Measurements [MMRM]). Analyses included the fixed, categorical effects for treatment, visit and the treatment-by-visit interaction. A common unstructured (co)variance structure was used to model within-patient errors. If the initial model failed to converge, the following correlation structures were tested in subsequent order until model convergence was achieved: autoregressive model of order 1 (AR[1]), Toeplitz, or CS (Compound Symmetry). The Kenward-Roger approximation was used to estimate the denominator degrees of freedom. GM, GM ratio, and GMFR estimates for each treatment are presented with the associated two-sided 95% Cis"

In Results, the following information has been added:

"With respect to IgG autoantibodies, there were no significant differences between the two study groups, except for CFFHP-IgG at month 24, which showed a lower GM of Δ optical density values in participants treated with abatacept than in those treated with hydroxychloroquine (GM ratio 0.18, 95% CI 0.05 to 0.59; p=0.0058). (Supplementary table 9).

There were significant differences between the two study groups in the CEP 1-IgA at month 3, which showed a lower GM of Δ optical density values in participants treated with abatacept compared with those treated with hydroxychloroquine (GM ratio 0.72, 95% CI 0.51 to 1.00; p=0.0471), and for the CFFHP-IgA at 24 months, which showed a lower GM of Δ optical density values in participants treated with abatacept compared with those treated with hydroxychloroquine (GM ratio 0.28, 95% CI 0.08 to 0.93; p=0.0374). (Supplementary table 10).

Regarding IgM autoantibodies, there were no significant differences between the two study groups in the Δ optical density values at any time point throughout the study. (Supplementary table 11)".

In Discussion, the following information has been added:

"In our exploratory analysis of the effects of abatacept on monotherapy on AMPA response in PR, we did not observe relevant differences in comparison with hydroxychloroquine".

3. Introduction: I would have welcomed more information about why HCQ was used as the comparator group in this trial. Is it currently used to treat PR?

This is implicitly mentioned in the introduction. However, we have expanded the information provided in the introduction, which currently reads as follows:

"In individuals with PR, antimalarials, such as hydroxychloroquine and chloroquine, are considered the best-studied therapeutic option, although there are no randomized clinical trials supporting their efficacy and safety[9]. In addition to its beneficial clinical effects in controlling palindromic symptoms, an observational study conducted in individuals with PR showed that those treated with antimalarials showed a significantly longer time to develop RA than those who were not treated with antimalarials. (Gonzalez-Lopez et al. J. Rheumatol. 27, 41–46 (2000)".

4. Figure 2: I would recommend that the n in both analyses is either included in the plot legend or just in the caption, to avoid confusion.

As suggested, we have modified the plot legend and caption. Currently, the number of patients in both analyses is included only in the caption.

5. Page 8: "The results in the per-protocol population showed a trend similar to that obtained in the mFAS population" – I would rewrite this as saying that the results were also statistically significant in the per-protocol population.

As suggested we have rewritten the sentence. Now, it reads as follows:

"Abatacept was also significantly superior to hydroxychloroquine in reducing the risk of developing RA during the first 12 months in the per protocol population".

6. Methods, randomisation: can more information be given about the randomisation technique? Was it complete randomisation, or stratified, or something else?

The section on randomization within Methods has been amended to include more information on the procedure. Currently, it reads as follows:

"Randomization codes were assigned centrally using electronic Case Report Form at the time of patient inclusion. These randomized codes were produced by PROC PLAN of the SAS system, with a 1:1 ratio of assignment between both arms, in blocks multiple of 2 elements and stratified by center. The center was included as a logistic stratification factor in the randomization procedure but was not included as a covariate in the main analysis following regulatory recommendations regarding the use of covariates (EMA/CHMP/295050/2013 Guideline on adjustment for baseline covariates in clinical trials. https://www.ema.europa.eu/en/adjustment-baseline-covariates-clinical-trials-scientific-guideline)".

7. Methods, outcomes: as per comment on the abstract, the secondary endpoints do not appear to correspond to the ones on the clinicaltrials.gov page. Could this be

clarified?

As we have mentioned in our response above, according to the protocol, effect of treatment on AMPA titers was a secondary objective. This endpoint was intended to serve the purpose of hypothesis generation (i.e., an exploratory endpoint) and was not included in the manuscript. However, following the reviewer's suggestion, we have included detailed information on this endpoint in the Methods and Results sections and a brief comment has been added in the Discussion section. Owing to its exploratory nature, we do not consider it necessary to include any mention in the abstract.

Please, see details in our response above.

Reviewer #2 **(Remarks to the Author):**

A: The results are clinically relevant for patients and health professionals.

B: The results are novel and as mentioned clinically relevant, also for the society in regards of health economics, since the cost of rheumatoid arthritis is rather high.

C: The study is presented in agreement with the EQUATOR group's CONSORT guidelines for randomized trials. Power calculations is obviously difficult to perform since this intervention has not been evaluated before, but appears to be sufficient in this case. The comparotor "plaquenil" is relevant since it is the most frequent and currently best validated treatment option for this condition.
D: Yes, and performed as presented in the pre-specified sigend statistical analysis plan, that also follw the CONSORT guideline proposed by the EQUATOR group.
E: The conclusions are hunble and relvant and in agreement with the presented results.
F: No suggestions for improvement.
G: No further references.
H: Clear abstract introduction and also visually well presented results, no further suggestions.

We thank the reviewer for the review and comments.

Reviewer #3 (Remarks to the Author):

A. Summary of key results
This randomized, open-label, multicenter phase IV trial (PALABA, NCT03669367, EudraCT 2017-004543-20) evaluated whether two years of abatacept (SC, 125 mg weekly then biweekly) could prevent progression from seropositive palindromic rheumatism (PR) to rheumatoid arthritis (RA), compared with oral hydroxychloroquine (HCQ, 5 mg/kg/day).
Among 70 patients (34 abatacept, 36 HCQ) included in the modified full analysis set (mFAS), 20.6% of abatacept-treated and 50.0% of HCQ-treated patients developed RA at 24 months (p=0.010; HR 0.27, 95% CI 0.07–0.96). Abatacept was also associated with higher remission (55.9% vs. 22.9%, p=0.007) and lower attack intensity. Both drugs were well tolerated; no unexpected safety issues emerged.

B.Originality and significance
The trial addresses a clinically relevant and underexplored population: individuals with seropositive PR, a recognized pre-RA phenotype. Prior prevention trials (PRAIRI, APIPPRA) demonstrated that biologics (rituximab, abatacept) could delay or prevent RA onset in ACPA-positive arthralgia without clinical arthritis. Extending this concept to PR is innovative.
However, the originality is partial, as abatacept's preventive effect in at-risk populations was already shown (e.g., Emery et al., Ann Rheum Dis 2022; Gerlag et al., Lancet 2019). The key novelty lies in targeting the PR subset, yet the sample size and open-label design substantially limit external validity.

Palindromic rheumatism is a clinical entity recognized by the WHO in the International Classification of Diseases (ICD)-10 under arthropathies and with the code M12.3. Finding an effective treatment for palindromic rheumatism, especially for preventing the development of RA, is an unmet medical need (Mankia and Emery. Nat Rev Rheumatol. 2019;15(11):687-695; Corradini et al. Semin Arthritis Rheum. 2021;51(1):266-277). Our study is the first randomized therapeutic trial conducted in this population. Therefore, in our view, our trial is a novelty and contributes to filling this gap.

C. Data & methodology
The study is a phase IV, multicenter, open-label, randomized controlled trial with 1:1 allocation, stratified by site (PROC PLAN in SAS, block randomization). Inclusion criteria (PR by Guerne & Weissman modified criteria, ACPA+ and/or RF+) are appropriate. Exclusion of seronegative PR was justified mechanistically.
Major methodological weaknesses:
-Open-label design without blinded assessment of the primary endpoint (transition to RA) introduces detection and expectation bias.

We agree with the reviewer on this point. This limitation has already been recognized in the manuscript, and its implications have been discussed. To emphasize the relevance of this limitation, we have slightly modified the text (underlined). Currently, it reads as follows:

“The lack of blinding is the **main** limitation of our study. Although blinding is a necessary methodological safeguard of a clinical trial, for some authors, blinding could have less of an impact on trials using an active comparator and those aimed at determining an intention-to-treat effect[30], such as in our trial. However, overestimation of the treatment effect and/or underestimation of treatment harm owing to the lack of blinding could be present in this trial”.

- Sample size (n=70) was below the prespecified target (35 per arm achieved, not exceeded), leaving limited power for secondary analyses or adjustment for confounders.

The small sample size is already recognized as a limitation in the manuscript: “The small sample size led to imprecision in the estimates, limiting the strength of the evidence provided by our study”. The issue of adjustment for confounders is commented below

- Outcome ascertainment relied on treating clinicians’ judgment using ACR/EULAR 2010 criteria, which is valid but prone to variability. No central adjudication was reported.

Consistent with the reviewer’s comment, we have added the following limitation to the corresponding paragraph of the Discussion:

“No central adjudication of the clinical endpoint was performed, which would have led to a more consistent evaluation and allowed a blinded evaluation”.

- Heterogeneity in baseline ACPA positivity (97% in HCQ vs. 82% in abatacept) might confound results, particularly as ACPA status strongly predicts RA progression.

As described in the Results: “A greater percentage of participants in the hydroxychloroquine group had a duration of attacks ≥72 hours within six months prior to inclusion (30.8% vs. 11.5%) and a higher frequency of ACPA positivity (97.2% vs. 82.4%)”.

Although proper randomization prevents selection bias and avoids systematic differences between treatment groups with respect to measured and unmeasured baseline characteristics that could influence the outcome of interest, it does not guarantee similarity between groups at baseline. However, if properly randomized, any differences in baseline characteristics are the result of chance rather than bias. It is important to note that an imbalance can exist in other unmeasured or unknown prognostic factors that could have a similar or opposite theoretical impact on the treatment effect.

Overall, the key issue is whether randomization has been properly generated and implemented, which we believe is the case in our trial.

Once an imbalance is detected, the issue is whether to perform an adjusted analysis. There is general agreement that adjustment for covariates should be prespecified in the protocol and/or the statistical analysis plan and that an imbalance in a baseline covariate “should not be considered an appropriate reason to include this baseline measure as a covariate in the primary analysis”.

References:

European Medicines Agency Guideline on adjustment for baseline covariates in clinical trials. Available at: https://www.ema.europa.eu/en/documents/scientific-guideline/guideline-adjustment-baseline-covariates-clinical-trials_en.pdf.

However, bearing in mind the comments of this reviewer and the suggestions from another reviewer, we performed sensitivity analyses of the primary outcome in two ways: adjusting for each single covariate and adjusting for the two covariates. The unadjusted OR for the occurrence of RA with abatacept vs. hydroxychloroquine was 0.259 (95%CI, 0.09 to 0.746). The adjusted ORs were 0.285 (95%CI, 0.097 to 0.839) when adjusting for ACPA positivity, 0.202 (95%CI, 0.065 to 0.628) when adjusting for duration of attacks ≥72 hours, and 0.220 (95%CI, 0.069 to 0.706) when adjusting for both covariates. Therefore, the results of the sensitivity analyses were consistent with those of the primary analysis.

We have included these results as supplementary information and the following information in the manuscript’s body, in the results section:

“A post-hoc analysis accounting for the imbalance in the two variables mentioned above (i.e., duration of attacks ≥72 h within six months prior to inclusion and frequency of ACPA positivity) was performed using logistic regression analysis in two ways: adjusting for each single covariate and adjusting for the two covariates. The results of the sensitivity analysis were consistent with those of the primary analysis (Supplementary Table 2-5)”.

- Duration of attacks ≥72h was more frequent in HCQ arm (30.8% vs. 11.5%), suggesting possible baseline imbalance toward more active disease in the control group.

The duration of flares >72 hours was longer in the hydroxychloroquine group than in the abatacept-treated group, as noted by the reviewer, although in all patients, the duration was less than one week, in accordance with the inclusion criteria. We do not know whether flare duration in this context is a risk factor for greater progression to RA. However, in anti-CCP–positive PR (slightly more frequent in the HCQ group), the characteristic flares are usually very short (<72 h), a clinical feature that distinguishes them from antiCCP negative individuals (Cabrera-Villalba et al. J Rheumatol 2014:41: 1650-5; Khabazzi et al Int J Rheum Dis 2012;15:427-430

However, as mentioned above, we performed a sensitivity logistic regression analysis including that covariate as a single covariate and adjusting for the two covariates that showed baseline imbalances. The unadjusted OR for the occurrence of RA with abatacept vs. hydroxychloroquine was 0.259 (95%CI, 0.09 to 0.746). The adjusted ORs were 0.202 (95%CI, 0.065 to 0.628) when adjusting for duration of attacks ≥72 hours, and 0.220 (95%CI, 0.069 to 0.706) when adjusting for ACPA positivity and duration of attacks ≥72 hours in the model. Therefore, the sensitivity analysis results were consistent with those of the primary analysis.

- The study lacked imaging (e.g., US/MRI) assessment of subclinical synovitis, a key predictor of progression.

In this trial, no imaging tests (US or MRI) were performed. The presence of subclinical synovitis (especially tenosynovitis) in patients with undifferentiated arthritis or anti-cyclic citrullinated peptide (anti-CCP)–positive arthralgia is a risk factor for progression

to rheumatoid arthritis (RA); however, this has been scarcely studied in palindromic rheumatism (PR). In fact, in PR, the presence of subclinical synovitis in the intercritical phase (outside of flares) is very uncommon (Cabrera-Villalba S et al. J Rheumatol 2014:41: 1650-5 ; Mankia et al. Ann Rheum Dis. 2019 Jan;78(1):43-50.), whereas during the acute phase, extracapsular involvement predominates. In a small number of patients with recent-onset PR who progressed to RA, Mankia et al. demonstrated the presence of synovitis during the flare phase [Mankia et al.et al. Ann Rheum Dis. 2019 Jan;78(1):43-50.]. However, in a study conducted by our group in patients with long-standing PR, the presence of subclinical synovitis in the intercritical phase was not associated with a higher risk of progression to RA (Sanmarti et al. Ann Rheum Dis. 2020 Mar;79(3):e30). Therefore, it remains unclear whether the presence of subclinical tenosynovitis/synovitis is a risk factor for RA progression in PR. We have added a comment on this issue when discussing the study limitations that reads as follows.

"In addition, due to this lack of imaging assessment, we did not evaluate the presence of subclinical synovitis, which is a potential prognostic factor. However, the scant literature on this topic provides inconsistent results on whether the presence of subclinical tenosynovitis/synovitis is a risk factor for RA progression in PR (Mankia et al. Ann Rheum Dis. 2019 Jan;78(1):43-50; Sanmarti et al. Ann Rheum Dis. 2020 Mar;79(3):e30).

The CONSORT checklist and protocol confirm correct ethical conduct, prospective registration, and pre-specified outcomes, but methodological rigor is weakened by open-label design, small sample, and lack of central outcome validation.

D. Appropriate use of statistics and treatment of uncertainties
The Statistical Analysis Plan (SAP, 25 June 2024) follows ICH E9 and ICHE91 recommendations, using SAS 9.4. Analyses included: mFAS and per-protocol (PP) populations; failure imputation and "available data only" (ADO) approaches; chi-square or Fisher's exact test for binary endpoints; Kaplan-Meier/log-rank for time-to-RA progression. While the statistical framework is technically sound, several issues arise:
- Multiplicity and interim analyses: No correction for multiple comparisons or exploratory analyses (p-values nominal).

There is only one variable and time-point defined as a primary endpoint (proportion of patients who develop persistent arthritis fulfilling the criteria of RA according to the EULAR/ACR 2010 classification criteria at any time during the 24 months follow-up); therefore, there is only one confirmatory p-value in the trial, and the rest of the p-values are considered descriptive at their nominal value.

We already mention the lack of multiplicity correction in the manuscript. However, we have added the following clarification to the Methods section:

"There is only one variable and time-point defined as a primary endpoint; thus, there is only one confirmatory p-value in the trial, and the rest of the p-values are considered descriptive at their nominal value".

- Confidence intervals are sometimes missing or reported only for hazard ratios.

The confidence intervals were included only for the effect sizes (i.e. risk differences and hazard ratios) for the analyses of the development of persistent arthritis during the 24 months of follow-up, either in the primary efficacy analysis (i.e., using the modified full analysis set with failure imputation) or the supportive analyses (i.e., per protocol analysis, analysis using the available data only, and survival analysis).

- No modeling of baseline imbalances (ACPA levels, attack duration).

As we mentioned above, following the reviewers' comments, we performed post-hoc sensitivity analyses in two ways: adjusting for each single covariate and adjusting for the two covariates. The unadjusted OR for the occurrence of RA with abatacept vs. hydroxychloroquine was 0.259 (95%CI, 0.09 to 0.746). The adjusted ORs were 0.285 (95%CI, 0.097 to 0.839) when adjusting for ACPA positivity, 0.202 (95%CI, 0.065 to 0.628) when adjusting for duration of attacks ≥72 hours, and 0.220 (95%CI, 0.069 to 0.706) when adjusting for both covariates. Therefore, the results of the sensitivity analysis were consistent with those of the primary analysis.

We have included these results as supplementary information and the following information in the manuscript's body, in the results section:

"A post-hoc analysis accounting for the imbalance in the two variables mentioned above (i.e., duration of attacks ≥72 h within six months prior to inclusion and frequency of ACPA positivity) was performed using logistic regression analysis in two ways: adjusting for each single covariate and adjusting for the two covariates. The results of the sensitivity analysis were consistent with those of the primary analysis (Supplementary Table 2-5).".

- No predefined subgroup analyses despite clear biological heterogeneity.

In most cases, subgroup analyses are exploratory. Due to the small sample size, it was not considered informative to predefine any subgroup analysis in the protocol.

- No analysis of ACPA titres—although listed in SAP and protocol—was presented in the manuscript, suggesting a deviation from the pre-specified plan.

The reviewer is right. As mentioned in the study procedures, serum biomarkers were tested at baseline and at 3, 12, and 24 months. According to the protocol, effect of treatment on AMPA titers was a secondary objective. This endpoint was intended to serve the purpose of hypothesis generation (i.e., an exploratory endpoint) and was not included in the manuscript. However, following the reviewer's suggestion, we have included detailed information on this endpoint in the Methods and Results sections. Owing to its exploratory nature, we do not consider it necessary to include any mention in the abstract.

In Methods we have included the following information:

"For evaluating the effect of study treatments on Anti-Modified Peptide/protein Antibodies (AMPAs) we determined by means of appropriate ELISA methods seven antigens (Supplementary Material) with different posttranslational modifications: 1) peptides bearing one posttranslational modification which included citrullinated peptides derived from vimentin (Vim-P55), α-enolase (CEP-1) and fibrin/filaggrin

(Chimeric Fibrin Filaggrin Citrullinated Peptide, CFFCP), and homocitrulinated peptide (Chimeric Fibrin Filaggrin Homocitrullinated Peptide [CFFHP]); 2) peptides bearing multiple posttranslational modifications which included Chimeric Fibrin Filaggrin Citrullinated Homocitrullinated Peptide (CFFCHP) and Chimeric Fibrin Filaggrin Citrullinated Homocitrullinated Acetylated Peptide (CFFCHAP); and 3) the protein antigen Carbamylated fetal calf serum (FCS-CarP). We used a peptide with unmodified basal structure, Chimeric Fibrin Filaggrin Peptide (CFFP) as a control peptide. For each antigen specificity, we determined three isotypes: IgG, IgA, and IgM. The effects of abatacept and hydroxychloroquine on autoantibody levels, specificities, and isotypes were evaluated at baseline and at 3, 12, and 24 months of follow-up for each patient included in the study.

For the analysis of the effect on AMPAs, each antigen-antibody pair was represented as a continuous variable, the Δ optical density. The Δ optical density was calculated by subtracting the optical density of each antigen-antibody pair without posttranslational modifications from the corresponding antigen-antibody pair with posttranslational modifications. Δ optical density measurements for each antigen-antibody pair at 0, 3, 12, and 24 months were analyzed using a parametric approach (see details below) on previously log-transformed data (Geometric Mean [GM]). Between-group and within-group comparisons were evaluated using the GM ratio and Geometric Mean Fold Rise (GMFR). The parametric approach consisted of a restricted maximum likelihood (REML)-based repeated measures approach (Mixed Models for Repeated Measurements [MMRM]). Analyses included the fixed, categorical effects for treatment, visit and the treatment-by-visit interaction. A common unstructured (co)variance structure was used to model within-patient errors. If the initial model failed to converge, the following correlation structures were tested in subsequent order until model convergence was achieved: autoregressive model of order 1 (AR[1]), Toeplitz, or CS (Compound Symmetry). The Kenward-Roger approximation was used to estimate the denominator degrees of freedom10. GM, GM ratio, and GMFR estimates for each treatment are presented with the associated two-sided 95% Cis"

In Results, the following information has been added:

"With respect to IgG autoantibodies, there were no significant differences between the two study groups, except for CFFHP-IgG at month 24, which showed a lower GM of Δ optical density values in participants treated with abatacept than in those treated with hydroxychloroquine (GM ratio 0.18, 95% CI 0.05 to 0.59; p=0.0058). (Supplementary table 9).

There were significant differences between the two study groups in the CEP 1-IgA at month 3, which showed a lower GM of Δ optical density values in participants treated with abatacept compared with those treated with hydroxychloroquine (GM ratio 0.72, 95% CI 0.51 to 1.00; p=0.0471), and for the CFFHP-IgA at 24 months, which showed a lower GM of Δ optical density values in participants treated with abatacept compared with those treated with hydroxychloroquine (GM ratio 0.28, 95% CI 0.08 to 0.93; p=0.0374). (Supplementary table 10).

Regarding IgM autoantibodies, there were no significant differences between the two study groups in the Δ optical density values at any time point throughout the study. (Supplementary table 11).

In Discussion, the following information has been added:

“In our exploratory analysis of the effects of abatacept on monotherapy on AMPA response in PR, we did not observe relevant differences in comparison with hydroxychloroquine”.**- Absence of power reassessment or interim futility check, although sample was slightly under target.**

Post-hoc power reassessment is not recommended because it is not helpful for interpreting the results and even could be misleading (Hoenig and Heisey. The American Statistician. 2001;55(1):19–24; Hopewell et al. BMJ. 2025 Apr 14;389:e081124). Thus, for instance, the ITEM 16a of CONSORT 2025 indicates that "There is no value in conducting and reporting a post hoc calculation of statistical power using the results of a trial, for example, as a pretext to explain non-significant findings; this may even mislead and confuse readers”

Incorporating a futility assessment can increase the efficiency of the trial, allowing trials that are unlikely to meet their objectives to stop early ultimately reducing costs, preserving resources, and limiting patient burden. However, it is not always necessary to perform a futility analysis, and there are circumstances in which it is better not to perform it. Other than efficiency reasons, two common reasons to perform it are when you are planning a large phase 3 trial with limited information from the phase 2, or in trials in patients with diseases associated with irreversible morbidity and mortality in which multiple products are under development and participation in a trial precludes the participation in another trial with perhaps a more promising product (Higgins et al. Stat Med. 2025 May;44(10-12):e70117). We believed that our trial did not correspond to any of those categories. More importantly, it should be borne in mind that this was a preventive trial in persons with an infrequent disease with a slow progression to rheumatoid arthritis, with no evidence-based treatments available and no other drugs under investigation. In such circumstances, an interim analysis at an early time point or put more weight on data at earlier time points than in a final analysis could pose an opportunity loss to see an important effect.

Thus, the statistical analysis is adequate but minimal, not fully exploiting available data nor addressing key uncertainties.

We have tried to address specific comments above. Overall, after including the analysis of AMPA titres in the revised manuscript, we believe the statistical analyses and results presented in the manuscript are consistent with those planned in the protocol and SAP.

E. Conclusions: robustness, validity, reliability
The conclusion that abatacept reduces progression from PR to RA is directionally plausible and supported by numerical differences;
However:
- The study’s open-label nature and small sample compromise robustness.
- The absolute event count (7 vs. 18 progressors) is low, with wide CIs.
- Potential selection and ascertainment bias (clinician-assessed RA diagnosis) likely inflate apparent effect size.
- Lack of biomarker correlation (ACPA titres, T-cell or B-cell modulation) weakens mechanistic inference.

Thus, while findings are hypothesis-generating, they do not provide definitive evidence of abatacept's preventive efficacy in PR.

The first three issues have been discussed in previous answers and recognized as study limitations in the manuscript.

Regarding the fourth issue, we believe that the presence of biomarker correlation would have reinforced our positive results. However, in our view, the lack of correlation does not necessarily diminish the value of our findings.

We agree that the study's limitations reduce to some extent the quality of the evidence. Although difficult to perform in this orphan indication, further randomized clinical trials that overcome the limitations of our study are desirable to confirm our results. However, we believe that our findings are more than hypothesis-generating and could be considered in clinical practice under certain circumstances.

F. Suggested improvements
For potential future trials:
- Blinded adjudication of primary endpoint (RA diagnosis by independent panel).
- Larger, placebo-controlled design or double-dummy comparison with blinded evaluation.
- Mandatory baseline imaging (US or MRI) to stratify subclinical synovitis.
- Quantitative ACPA titres longitudinal analysis, as per SAP.
- Standardization of attack diaries and validated patient-reported outcomes.
- Longer post-treatment follow-up to assess durability of preventive effect.
- Centralized statistical analysis with covariate adjustment (e.g., mixed logistic or Cox models).

With certain nuances (namely, stratify subclinical synovitis and covariate adjustment), which are further explained in other responses to the reviewers, we agree with most recommendations for potential future trials. Moreover, all of them are currently discussed in the paragraph devoted to study limitations. Therefore, the reader is informed of the knowledge gaps for future trials.

G. References and credit to previous work
The authors cited major relevant trials (PRAIRI, APIPPRA) and background studies on PR and ACPA, but key mechanistic or biomarker references (e.g., T-cell exhaustion under abatacept, preclinical autoimmunity networks) could be expanded.

According to the reviewer's comment, we have added to the Discussion, and based on recent articles, we have added relevant information from a mechanistic perspective on the preclinical phase of RA and its possible modulation with abatacept treatment to prevent the development of RA.

"Recently, studies applying multi-omic methodologies in people at risk of RA who progress to RA have shed light on the immunobiology of this transition stage (He, Z. et al. Progression to rheumatoid arthritis in at-risk individuals is defined by systemic inflammation and by T and B cell dysregulation. Sci. Transl. Med. 17, eadt7214 (2025)..

ACPA+ individuals at risk of RA were characterized by progressive systemic inflammation and dysregulation of B and T cell subset populations during the preclinical phase, which was associated with RA progression;the authors alsocompared their results with those from clinical trials in which RA patients were treated with abatacept or, a tumour necrosis factor inhibitor (TNFi). The genetic signatures of naive and central memory CD4 T cells found during progression to clinical RA were significantly modulated by treatment in abatacept responders, but not with TNF inhibitors. Furthermore, abatacept-induced changes were observed in genes previously implicated in RA-like diseases (CD8a, CD99, CDK4, CXCR3, FLT3LG, GZMM, LTB, and TNFRSF14) and in those related to the Th17 pathway. Importantly, the progression to RA signatures of naive and central memory CD4 T cells are 20 times more likely to be reversed with abatacept treatment compared to TNFi. These data suggest that T cell mechanisms relevant to clinical RA may contribute to disease pathogenesis in the preclinical phase and may provide mechanistic evidence supporting the role of abatacept in delaying the onset of clinical RA (Hitchon CA, El-Gabalawy HS. Advances in understanding preclinical rheumatoid
arthritis and prospects for prevention. Nat Rev Rheumatol. 2025 Dec 15. doi: 10.1038/s41584-025-01342-6"

H. Clarity and context
- The abstract and introduction correctly described rationale and results. However:
- The abstract overstates the robustness of conclusions given design limitations.

We have modified the sentence by using the past tense, thus making our conclusion more descriptive. It currently reads:

"In patients with seropositive PR, compared with hydroxychloroquine, abatacept given for 2 years reduced the risk of progression to RA and improved symptoms".

- The methods section lacks detail on randomization concealment and handling of missing data.

The section on randomization within Methods has been amended to include more information on the procedure. Currently, it reads as follows:

"Randomization codes were assigned centrally using electronic Case Report Form at the time of patient inclusion. These randomized codes were produced by PROC PLAN of the SAS system, with a 1:1 ratio of assignment between both arms, in blocks multiple of 2 elements and stratified by center. The center was included as a logistic stratification factor in the randomization procedure but was not included as a covariate in the main analysis following regulatory recommendations regarding the use of covariates".

The methods for handling missing data are already described in the manuscript. It reads as follows: "Missing data on progression to RA were imputed as treatment failure (failure imputation) for primary analysis, and sensitivity analysis was performed using the available data only (ADO) approach".

- Tables and supplementary materials are clear, but figure legends should specify the analysis population and statistical test used.

As suggested by the reviewer, all figure legends currently contain the analysis populations and statistical tests used.

- The discussion could better contextualize findings within the broader “RA prevention” field, acknowledging the exploratory nature.

As mentioned above, palindromic rheumatism is a clinical entity recognized by the WHO in the International Classification of Diseases (ICD)-10 under arthropathies. We believe that the discussion should first contextualize our results within the available evidence for that clinical entity. We also discussed the results in the context of RA prevention.

Our study was not designed as an exploratory trial. We agree that the study’s limitations reduce to some extent the quality of the evidence. However, we believe that our findings are more than hypothesis-generating and could be considered in clinical practice under certain circumstances.

Reviewer #4 (Remarks to the Author):

This is an interesting and well-written paper. It is a randomized clinical trial comparing abatacept with hydroxychloroquine for the prevention of progression to rheumatoid arthritis in patients with palindromic rheumatism.

Although a randomized trial, the sample size is relatively small. However, PR is a rare disease and has been poorly studied with no previous randomized trials. As such this is an important study. However there are a number of major limitations:

1. Unlike recent RA prevention trials, abatacept has been administered for two years rather than one. There is no observational period after therapy. Instead, in the second year the dose is reduced to alternate weekly - there is no explanation for this rationale. Why did the authors decide to do this? If abatacept were to be used in clinical practice, what regimen would be proposed?

Thank you for bringing this issue to our attention. This was one of the questions considered when designing this study. The goal was to compare the two treatments (unlike other prevention trials, there was no placebo arm but rather an active treatment). The rate of progression to rheumatoid arthritis in palindromic rheumatism is higher during the first 2–3 years after symptom onset, which is why we decided to maintain abatacept treatment throughout the entire study period (2 years). The dose used was the one approved for rheumatoid arthritis (weekly dosing), but we do not know whether in this condition—at this earlier phase with a lower inflammatory burden such as in PR—a longer dosing interval (as it is done when optimizing biologic therapies in arthritis once remission is achieved), for example, every 2 weeks, could also be effective. This could potentially reduce the rate of adverse events, lower economic costs, and possibly lead to greater patient acceptance.

It is difficult to recommend a dose for clinical practice based on these study results. However, because dosing every 2 weeks was used during the second phase of the study (12–24 months), and some patients developed RA during that period, this could suggest that weekly dosing could be more effective. As the reviewer rightly points out, it is important to understand what happens once treatment is discontinued. A 5-year follow-up of the study is planned in this regard, as mentioned in the Discussion section.

To clarify the first issue we have added the following information on the Methods section:

“The reason for maintaining the dose for two years was that the progression to RA in individuals with PR is higher during the first 2–3 years (Koskinen E at al J Rheumatol 2009;36: 1873-1875) after the onset of symptoms. Because in the early phase of PR the inflammatory burden is low, following one of the strategies in patients with RA for optimizing biologic therapies once remission is achieved, we decided to reduce the dose of abatacept for the second year of treatment (i.e., every two weeks administration)”.

2. The groups are not balanced for ACPA. This is potentially important as there us a higher proportion of ACPA+ patients in the HCQ arm. Given ACPA is a major risk factor for progression to RA, how did the authors adjust for this?

As described in the Results: “A greater percentage of participants in the hydroxychloroquine group had a duration of attacks ≥72 hours within six months prior to inclusion (30.8% vs. 11.5%) and a higher frequency of ACPA positivity (97.2% vs. 82.4%)”.

Although proper randomization prevents selection bias and avoids systematic differences between treatment groups with respect to measured and unmeasured baseline characteristics that could influence the outcome of interest, it does not guarantee similarity between groups at baseline. However, if properly randomized, any differences in baseline characteristics are the result of chance rather than bias. It is important to note that an imbalance can exist in other unmeasured or unknown prognostic factors that could have a similar or opposite theoretical impact on the treatment effect.

Overall, the key issue is whether randomization has been properly generated and implemented, which we believe is the case in our trial.

Once an imbalance is detected, the issue is whether to perform an adjusted analysis. There is general agreement that adjustment for covariates should be prespecified in the protocol and/or the statistical analysis plan and that an imbalance in a baseline covariate “should not be considered an appropriate reason to include this baseline measure as a covariate in the primary analysis”.

References:

European Medicines Agency Guideline on adjustment for baseline covariates in clinical trials. Available at: https://www.ema.europa.eu/en/documents/scientific-guideline/guideline-adjustment-baseline-covariates-clinical-trials_en.pdf.

However, bearing in mind the comments of this reviewer and the suggestions from another reviewer, we performed sensitivity analyses of the primary outcome in two ways: adjusting for each single covariate and adjusting for the two covariates. The unadjusted OR for the occurrence of RA with abatacept vs. hydroxychloroquine was 0.259 (95%CI, 0.09 to 0.746). The adjusted ORs were 0.285 (95%CI, 0.097 to 0.839) when adjusting for ACPA positivity, 0.202 (95%CI, 0.065 to 0.628) when adjusting for duration of attacks ≥72 hours, and 0.220 (95%CI, 0.069 to 0.706) when adjusting for both covariates. Therefore, the results of the sensitivity analyses were consistent with those of the primary analysis.

We have included these results as supplementary information and the following information in the manuscript’s body, in the results section:

“A post-hoc analysis accounting for the imbalance in the two variables mentioned above (i.e., duration of attacks ≥72 h within six months prior to inclusion and frequency of ACPA positivity) was performed using logistic regression analysis in two ways: adjusting for each single covariate and adjusting for the two covariates. The results of the sensitivity analysis were consistent with those of the primary analysis (Supplementary Table 2-5).”.

3. A major issue with this trial is the recording of symptoms (i.e. flares). Patients were seen periodically only. How were flares recorded? Were patients given diaries to fill in or was it recorded on an app? Recounting flares is notoriously difficult when done retrospectively and patients can forget the number of flares they have had. Similarly how was intensity measured? Was this an average intensity? Flares can vary in intensity and if patients have multiple flares between visits how did they report intensity which may have been variable?

Thank you for this interesting observation. We agree with the reviewer that collecting disease flare data in a fully reliable manner can be challenging. Flares were not recorded via a mobile application, and, although several patients documented their flares, no specific patient diary was designed for this purpose. Instead, data were collected by the treating physicians at each follow-up visit.

At the baseline visit, all patients were instructed that at each subsequent visit, the number of flares, their anatomical location, duration, intensity, and the presence of inflammatory signs would be recorded, as well as whether the flares were monoarticular or involved more than one joint simultaneously. The intensity and duration of each flare and affected location were recorded separately.

As pointed out by the reviewer, we cannot completely rule out the possibility that some flares were not captured in some patients. However, we consider this highly unlikely, as patients were seen regularly every three months and the overall number of flares in both treatment arms was low (median of 0.3 flares per month).

4. What was flare intensity designed to measure? Flares are multi-faceted and can include pain, swelling, redness etc. How are each of these features recorded? e.g. if a flare was associated with lots of swelling but minimal pain, would that be recorded differently to a flare with pain but no swelling? This is an important area to consider but there is minimal information provided.

The patient was specifically asked how intense the flare had been (regardless of the presence or absence of perceived inflammatory signs, its location, or its duration, which were recorded separately. Intensity of each crisis was recorded using a visual analogue scale (0-10). Although this was not explicitly stated in the question, it is understood that 'intensity' essentially referred to the degree of pain and/or functional impairment experienced by the patients

We agree with the reviewer that the manuscript does not contain sufficient information in this regard. Therefore, we have added the following information (underlined) to the Methods section:

"Participants were seen at screening, baseline and every three months thereafter until the end of the study or at the time of treatment discontinuation or early withdrawal. Concomitant medication, general physical examinations, <u>characteristics of the attacks (i.e., number, duration, intensity as evaluated using a visual analog scale, perceived inflammatory joint signs, and joints involved),</u> joint assessments, and assessments of the American College of Rheumatology/European League Against Rheumatism (ACR/EULAR) criteria for RA were recorded at each study visit".

5. Importantly there was no difference in median number of flares between the two arms. This could be interpreted as no difference in the effect of abatacept vs hydroxychloroquine on the treatment of PR as patients are often most concerned about how often their flares occur.

This is an interesting observation. When the number of flares was analyzed in the two treatment arms, no significant differences were observed between the groups in terms of the number of flares (number of attacks per month), although the count was numerically

lower in the abatacept group. However, when we analyzed the frequency of remission, defined as no or a single palindromic attack during at least 12 months, the difference was large and statistically significant in favor of abatacept compared to HCQ (55.9% vs. 22.9%). It is likely that the low number of flares at baseline in both groups (fewer than one flare per month) and during the follow-up made difficult to detect any further decrease and thus significant differences in symptom improvement. In addition, we believe that persistent remission is likely to be more relevant from the patient's perspective than a reduction in the number of flares.

6. Another major concern is permitted use of low dose steroid, colchicine and NSAID, all of which can reduce flare intensity and potentially even the frequency if used regularly. How did use compare between the arms?

Thank you for this observation. All concomitant pharmacological treatments were analyzed for each patient and at every follow-up visit, with special attention to the use of NSAIDs and glucocorticoids (according to the protocol, the use of oral glucocorticoids was allowed at a maximum dose of 7.5 mg/day of prednisone and for the shortest possible duration). A supplementary table has been added specifying the percentage of patients who used colchicine, NSAIDs, and glucocorticoids regardless of their indication throughout the study period. Overall, the use of anti-inflammatory drugs, especially glucocorticoids was less frequent among abatacept-treated individuals.

Since this information was not a secondary outcome, we have included the following information in a descriptive manner in the Results section, and no comments have been included in the Discussion section. The following paragraph has been added to the Results section:

"The use of allowed anti-inflammatory therapy (i.e., colchicine, oral corticosteroid, and nonsteroidal anti-inflammatory drugs) throughout the study period was less frequent among individuals treated with abatacept than among those treated with hydroxychloroquine, except for colchicine, with one patient treated in the abatacept group compared to none in the hydroxychloroquine group (Supplementary Table 1). Regardless of the indication, less participants received oral corticosteroid among those treated with abatacept (n=7, 20.6%) than among those treated with hydroxichlorquine (n=19, 52.8%) (Supplementary Table 1).

Supplementary table 1

Use of anti-inflammatory therapy throughout the study period (mFAS population)

Treatment	Abatacept (N=34) Subjects (%)	Hydroxychloroquine (N=36) Subjects (%)
Colchicine	1 (2.9%)	0 (0.0%)
Oral glucocorticoids	7 (20.6%)	19 (52.8%)
NSAIDs	21 (61.8%)	25 (69.4%)

NSAIDs, nonsteroidal anti-inflammatory drugs

7. What was median time to progression to RA between the two groups?

The median time to progression to RA was not reached in any study group. In the manuscript we provided the hazard ratio as effect measure. In addition, we have

calculated the restricted mean survival time in a post-hoc analysis. The restricted mean survival time was 19.7 months with hydroxychloroquine and 23.8 months with abatacept, for a difference in restricted mean survival between groups of 4.0 months (95% CI 1.4 to 6.6; p=0.0025) at 24 months.

Because we are providing the hazard ratio in the manuscript (and the proportional hazards assumption was met), we believed that it is not necessary to include this information in the manuscript.

8. primary outcome was progression to RA. If patients had swelling at one of the study visits, were they recalled to be reassessed? How was this done? What if there was persistent synovitis but the patient did not meet ACR EULAR criteria for RA?

Thank you for the comment. As described in the study protocol, patients with palindromic rheumatism who had an attack lasting more than one week were excluded from the study (an exclusion criterion at enrollment); however, this did not apply if it occurred during follow-up. Three patients (one treated with HCQ and two with abatacept) developed a flare lasting more than one week but did not meet the ACR/EULAR criteria during follow-up. Only in one of these three cases, who was on abatacept therapy, did the arthritis last for more than 3 months, affecting only the second metatarsophalangeal joint. MRI was performed, which showed intermetatarsal bursitis rather than joint synovitis, and this finding persisted until the end of follow-up.

9. Table 1 should include p values to evaluate between-group differences. Number of joints involved in a flare should be included. Concomitant steroid, colchicine and NSAID use should be compared formally.

Although commonly performed, as the CONSORT statement indicates, significance testing of baseline differences is not recommended and should not be reported (Reference: Hopewell et al. BMJ 2025;389:e081124).

As suggested, the number of joints involved has been included in Table 1, categorizing them as monoarticular and oligo/ polyarticular, which was similar in both treatment groups.

Likewise, the number of patients previously treated with NSAIDs, or oral glucocorticoids has been also specified in Table 1.;

10. How do the authors interpret cholesterol and lymphocyte count changes in the ABT arm?
In our opinion, the magnitude of the changes observed in the abatacept arm on cholesterol levels or lymphocyte count was not relevant. A slight increase in cholesterol levels has been reported in patients with RA after abatacept treatment, but with a conserved atherogenic index (slight increase in HDL levels and decrease in LDL levels). In our study, a complete lipid profile was not obtained. In previous trials with abatacept, no significant associations with the modification of leukocyte or lymphocyte populations have been described. The slight increase in lymphocyte count (median 1.87 to 2.07) could be a chance finding.

Abatacept for prevention of rheumatoid arthritis in individuals with palindromic rheumatism: a randomized trial " NMED-A145314

Response to referees

Response to Editor

Thank you for your revised manuscript, as you can see the reviewers have largely signed off on the manuscript. We would however ask you provide responses to the outstanding reviewer comments below.
Of particular concern is the third comment from reviewer #4. The manner in which the primary outcome was assessed – how were authors (or investigators) able to distinguish between a flare and transition to RA? Please note, if there is uncertainty in how patients' symptoms were classified we may be unable to proceed with this manuscript.

We believe that our detailed response to reviewer #4 shows that, as stated in the manuscript, participants who were classified as cases of rheumatoid arthritis met two criteria: the criterion for persistent arthritis (i.e., lasting at least one week in the same joint) and the 2010 ACR/EULAR criteria for rheumatoid arthritis (i.e., the criteria established in the protocol), confirming the occurrence of rheumatoid arthritis. It should be noted that palindromic flares are typically monoarticular and very short-lived, and it is exceptional for them to last more than one week in the same joint. In addition, actual data show that, at the time of classifying the participant as case of rheumatoid arthritis, all patients experienced persistent arthritis lasting at least two weeks and met the ACR/EULAR criteria for rheumatoid arthritis. Therefore, the diagnosis of rheumatoid arthritis was robust. Moreover, all those participants classified as showing rheumatoid arthritis initiated treatment for rheumatoid arthritis. Finally, it is worth mentioning that at the time of preparing this response to the reviewers, the diagnosis of rheumatoid arthritis was maintained by the rheumatologists in all 13 patients. Please see our detailed response to the reviewer.

In addition to this point, and the other outstanding reviewer reports, we have some additional formatting points

1. In the abstract, please either list all secondary endpoints or none at all. We appreciate there is a desire to show secondary endpoints in the abstract but, to prevent concerns of cherry-picking, we ask authors are not selective in what secondary endpoints are shown. The word limit of the abstract may preclude mention of all secondary endpoints in the abstract – if this is the case, only mention primary endpoints here.

As stated in the manuscript, "secondary outcomes included the frequency, intensity and duration of joint attacks and the proportion of patients in remission, defined as no or a single palindromic attack during at least 4 consecutive visits (i.e., 12 months). We also evaluated the frequency of adverse events. As an exploratory outcome, we evaluated the effect of treatment on AMPA titers".

We have added the results of the single secondary outcome that was not included in the abstract: the frequency of flares. In addition, as suggested by reviewer #3, we have also included the results of the effect on autoantibodies. Thus, the sentences in the abstract are now as follows:

"Abatacept was also associated with a reduced intensity of joint attacks and a higher frequency of symptom remission; however, there were no differences in the frequency of attacks between

the two study drugs. No relevant differences in the evolution of AMPA antibody titers were observed between the two treatment arms.

2. Similar, for the manuscript – all secondary endpoints must be mentioned (or again, none of them). We appreciate the authors have included additional endpoints that were not present in a previous version. This point here is included for clarity in light of the first editor point.

To the best of our knowledge, in addition to post hoc analyses of the primary outcome, there were only two amendments to the reporting of outcomes in the previous revised version:

-To explicitly include the results of the per-protocol analysis of the primary outcome. In the previous version, they were included but referred the reader to the supplementary material (It read: “The results in the per-protocol population showed a trend similar to that obtained in the mFAS population (Supplementary table 1)”).

-To include the results of the exploratory outcome regarding the effect on autoantibodies.

Both amendments were requested by the reviewers. All secondary outcomes have been reported in the manuscript.

3. Thank you for including the post-hoc analysis and labelling it as such.

Reviewer #3 (Remarks to the Author):

The revised manuscript reports a randomized, open-label phase IV trial showing reduced 24-month progression to RA with abatacept versus hydroxychloroquine (20.6% vs 50.0%; risk difference 29.4%, 95% CI 8.2–50.7; HR 0.27). Sensitivity analyses adjusting for baseline imbalances are now included and remain consistent with the primary findings.

- The study remains clinically relevant as the first randomized trial conducted specifically in palindromic rheumatism (PR). Although RA-prevention strategies with abatacept have been tested in at-risk arthralgia populations (APIPPRA, ARIAA), extension to PR is novel and addresses an unmet need.

- The authors have adequately clarified the randomization procedure, acknowledged the lack of blinding and central adjudication, and explicitly discussed sample size limitations. The addition of post-hoc adjusted analyses meaningfully strengthens the robustness of the primary outcome.

- Inclusion of the previously omitted AMPA (autoantibody) analyses improves transparency and alignment with the protocol. The exploratory results show limited modulation and are appropriately interpreted cautiously.

- Statistical reporting has improved: confidence intervals are now provided for primary effect sizes, and the manuscript clearly states that only one confirmatory primary endpoint exists, with all other p-values considered descriptive.

- The absence of multiplicity correction for secondary endpoints is now transparent and methodologically acceptable given the single primary endpoint structure.

- The conclusions are generally balanced and acknowledge key limitations (open-label

design, no imaging, no central adjudication, modest sample size). The statement regarding potential clinical use of abatacept could remain cautious but is reasonably framed.

-Minor suggestion: consider briefly mentioning exploratory autoantibody findings in the abstract for completeness and clarifying in the Discussion that secondary analyses are descriptive.

In response to the suggestion of the reviewer, we have added the following information to the abstract:

"No relevant differences in the evolution of AMPA antibody titers were observed between the two treatment arms"

Regarding the discussion of secondary analyses, as suggested, the two sentences on secondary endpoints have been modified as follows:

-First paragraph of the discussion

This sentence read: "The results of this open-label, randomized clinical trial revealed that compared with hydroxychloroquine, abatacept reduced the risk of developing RA in patients with PR at risk of disease occurrence. This benefit extended to a significantly greater improvement in PR symptoms among abatacept-treated individuals. Both drugs were well tolerated over a period of two years"

Currently reads: "The results of this open-label, randomized clinical trial revealed that compared with hydroxychloroquine, abatacept reduced the risk of developing RA in patients with PR at risk of disease occurrence. This benefit appeared to extend to a greater improvement in PR symptoms among abatacept-treated individuals. Both drugs were well tolerated over a period of two years."

Fourth paragraph of the discussion (lines 20-24):

It read: "In this regard, it is important to note that in our trial, the benefits of abatacept extended to short-term outcomes, such as a significant improvement in the severity of the flares and a higher frequency of remission than hydroxychloroquine did".

Currently it reads: "In this regard, it is important to note that in our trial, the benefits of abatacept appeared to extend to short-term outcomes, such as greater improvement in the severity of flares and a higher frequency of remission than hydroxychloroquine did.".

Overall, the authors have satisfactorily addressed the main methodological and statistical concerns raised in the initial review. The revised manuscript is substantially improved.

Reviewer #4 (Remarks to the Author):

Some of the points have been addressed. However, there are still major concerns, particularly related to two areas:

1/ The authors acknowledge that participants were not offered flare diaries or apps to record their flares between visits. In my view this is a major limitation as it brings into question the validity of assessment of symptom improvement in the trial. They say that participants were instructed that various factors about their flares (frequency, duration, location, intensity etc) would be recorded at study visits but there was no assistance or guidance given to patients or investigators about how to do this in a standardized way.

This is very likely to have led to inconsistent data capture across the multiple centers involved. They mention a low median frequency of flares in each arm, but the range is quite wide and some patients were having one or two attacks per month and so would have potentially been affected.

As mentioned in the next response, the characteristics of flares were recorded in a standardized manner by the investigators in the electronic case report form. However, we agree that the way in which the patients reported the number and characteristics of flares, despite being instructed during the baseline visit, could lead to somewhat heterogeneous reporting of this secondary endpoint. In contrast, we believe that the impact on other closely related secondary endpoint, namely, remission, was minimal. Remission was defined as “no or a single palindromic attack during at least 4 consecutive visits (i.e., 12 months)”. In the worst-case scenario, this means that for at least three visits, the patient reported no flares in two visits and one flare in a 3-month period between two visits. We believe that reporting no flares or only one flare is fairly reliable and likely to be consistent across patients and centers. In addition, remission is an endpoint more relevant for patients because to some extent it reflects absence of disease activity.

2/ The authors acknowledge that flare 'intensity' was not defined and is subject to interpretation. As such it is likely to have led to inconsistent data capture across the trial. They have added a sentence in the methods that is mis-leading as the investigators were not told to do this in any standardised way.

The characteristics of flares were recorded in a standardized manner by the investigators in the electronic case report form. We have modified that sentence to make it clear that the information was derived from the clinical interview and to explain the meaning of ‘intensity’. The paragraph now reads as follows:

“Participants were seen at screening, baseline and every three months thereafter until the end of the study or at the time of treatment discontinuation or early withdrawal. Concomitant medication, general physical examinations, characteristics of the attacks as elicited from the anamnesis (i.e., number, duration, intensity as evaluated using a visual analog scale, presence and perceived inflammatory joint signs, and joints involved), joint assessments, and assessments of the American College of Rheumatology/European League Against Rheumatism (ACR/EULAR) criteria for RA were recorded at each study visit; although it was not explicitly stated in the question, it was understood that ‘intensity’ essentially referred to the degree of pain and/or functional impairment experienced by the patients”.

It is important to note that information on the characteristics of attacks was recorded by the investigators in a standardized manner in the electronic case report form. All such information was explicitly recorded in the electronic case report form.

3/ The question about assessment of the primary outcome has not been answered is a satisfactory way. What if an ACPA+ PR patient presented at the 3 month assessment with three swollen small joints? How would the investigator know if this patient was in a flare or whether they had now developed RA? Either way theuy would classify as ACR-EULAR RA on the day of assessment. The authors need to clarify the instructions provided to investigators to be able to distinguish PR flare from RA development.

In the response we provided in the previous revision, we focused mainly on discussing what happened in those patients with persistent arthritis who did not meet the ACR/EULAR 2010 RA classification criteria during follow-up, as we believed that was the reviewer’s question. However, the reviewer is correct that we did not address in detail the first part of the issue that has now been raised; that is, what happens if, at a follow-up visit, a patient had one or more

swollen joints and, although they fulfilled rheumatoid arthritis criteria, how could it be determined whether they were experiencing a flare of palindromic rheumatism at that specific visit rather than persistent arthritis, while still meeting the ACR/EULAR criteria.

The protocol specified that patients were classified as having rheumatoid arthritis if they met two criteria: having persistent arthritis and fulfilling ACR/EULAR criteria for rheumatoid arthritis. This is also mentioned in the manuscript when defining the primary outcome (i.e. the primary outcome was the development of persistent arthritis that fulfilled the 2010 RA classification criteria of the ACR/EULAR as evaluated by the participant clinicians") and as such, the two criteria were explicitly recorded in the case report form. We have added the specification of the duration to be defined as persistent arthritis (i.e. lasting more than one week in the same joint) when defining the outcome; it was defined previously in the manuscript and explicitly stated in the eCRF, but we believe it is clearer if we specify this issue in the outcome. Palindromic flares are typically monoarticular and very short-lived; it is exceptional for them to last more than one week in the same joint. In addition to meeting this criterion, all investigators were instructed to consider this aspect and to specifically assess, at the time of evaluation, the exact duration of arthritis (joint with inflammatory signs) up to the visit. The duration of arthritis was, therefore, considered an essential criterion for classifying the patient who progressed to RA. In the attached table, we describe for each patient who was diagnosed with rheumatoid arthritis (based on both criteria) the tender and swollen joint counts at that visit, as well as the duration of arthritis (presence of inflammatory signs) in weeks. The number of swollen joints ranged from 1 to 10, and the duration of persistent arthritis was at least 2 weeks (between 2 and 12 weeks) in all cases.

Additionally, to prepare this table, we recorded the subsequent treatment initiated immediately after the withdrawal of study treatment. In all cases, in accordance with clinical practice in the treatment of rheumatoid arthritis, the investigator started treatment with a csDMARD (methotrexate in 12 patients and leflunomide in 1 patient).

**Hospital**	**Date of Dx**	**TJC**	**SJC**	**Duration of arthritis (weeks)**	**Initial Rx after Dx**
1	15/02/22	5	3	4	MTX
1	29/01/21	4	3	8	MTX
1	18/03/22	3	2	2	MTX
1	03/05/23	6	6	6	MTX
2	28/01/20	5	10	4	LFN
3	15/09/21	6	1	6	MTX
3	14/12/22	2	3	7	MTX
4	22/02/22	2	2	8	MTX
4	24/08/21	2	1	6	MTX
5	31/03/22	6	4	6	MTX
6	24/01/23	7	5	2	MTX

6	14/03/23	6	6	4	MTX
7	9/11/22	1	1	12	MTX

Dx, diagnosis; LFN, leflunomide; MTX, methotrexate; Rx, treatment; SJC, swollen joint count; TJC, tender joint count

In summary, we believe that there was no possible confusion between a palindromic flare and transition to rheumatoid arthritis in the patients who ultimately were classified as having rheumatoid arthritis in this study, and the diagnosis of rheumatoid arthritis was robust. Finally, it is worth mentioning that at the time of preparing this response to the reviewers, the diagnosis of rheumatoid arthritis was maintained by the rheumatologists in all 13 patients.
